# Electromyographic activity in the *gluteus medius*, *gluteus maximus*, *biceps femoris*, *vastus lateralis*, *vastus medialis* and *rectus femoris* during the *Monopodal Squat*, *Forward Lunge* and *Lateral Step-Up* exercises

**DOI:** 10.1371/journal.pone.0230841

**Published:** 2020-04-01

**Authors:** José M. Muyor, Isabel Martín-Fuentes, David Rodríguez-Ridao, José A. Antequera-Vique

**Affiliations:** 1 Laboratory of Kinesiology Biomechanics and Ergonomics (KIBIOMER Lab.), Research Central Services, University of Almería, Almería, Spain; 2 Health Research Centre University of Almería, Almería, Spain; Hochschule Trier, GERMANY

## Abstract

The *Monopodal Squat*, *Forward Lunge* and *Lateral Step-Up* exercises are commonly performed with one's own body weight for rehabilitation purposes. However, muscle activity evaluated using surface electromyography has never been analyzed among these three exercises. Therefore, the objectives of the present study were to evaluate the amplitude of the EMG activity of the gluteus medius, gluteus maximus, biceps femoris, vastus lateralis, vastus medialis and rectus femoris muscles in participants performing the *Lateral Step-Up*, *Forward Lunge* and *Monopodal Squat* exercises. A total of 20 physically active participants (10 men and 10 women) performed 5 repetitions at 60% (5 repetition maximum) in each of the evaluated exercises. The EMG amplitude was calculated in percentage of the maximum voluntary contraction. The *Monopodal Squat* exercise showed a higher EMG activity (*p* ≤ 0.001) in relation to the *Lateral Step-Up* and *Forward Lunge* exercises in all of the evaluated muscles (*d* > 0.6) except for the rectus femoris. The three exercises showed significantly higher EMG activity in all of the muscles that were evaluated in the concentric phase in relation to the eccentric one. In the three evaluated exercises, vastus lateralis and vastus medialis showed the highest EMG activity, followed by gluteus medius and gluteus maximus. The *Monopodal Squat*, *Forward Lunge* and *Lateral Step-Up* exercises not only are recommended for their rehabilitation purposes but also should be recommended for performance objectives and strength improvement in the lower limbs.

## Introduction

Counterresistance training constitutes one of the basic pillars of maintaining an optimal physical condition [1], producing increased muscle mass and improvements in muscle strength [2], being of great importance for increasing athlete performance and in the prevention and recovery of possible injuries [3]. In this sense, the *Squat* and *Deadlift* exercises and their variants, such as the *Parallel Back Squat* [4], *Partial Back & Full Back Squat* [5], *Hexagonal Barbell* and *Straight Deadlift* exercises [6], are commonly used in muscle conditioning as basic practices for lower-body strength training [7,8].

Superficial electromyography (sEMG) is the most common technique to evaluate the interaction among the muscle groups that are involved in an exercise [9,10]. From the sEMG signal, we can determine the electromyographic (EMG) activity [9] that occurs in each repetition. Properly employed, sEMG can determine which muscles are active, their degree of activity, and how active the muscle is compared to the subject´s capacity [11]. Based on these results, the best exercises may be selected according to the training objectives [12,13].

Recently, there has been a significant increase in the variability of the execution of these exercises and/or movements in physical conditioning [14], such as those performed that are performed unilaterally in the *Step-Up* or *Lunge* exercises [15,16]. Numerous authors have attempted to demonstrate the need to incorporate unilateral exercises in strength training due to the applicability of these exercises to sports and to daily activity [17–19].

In this sense, several studies have compared EMG activity in participants performing unilateral exercises, such as *Unilateral Squat*, *Unilateral Deadlift* and *Rear Cross Over Lunge* [20], or *Forward Lunge* vs. *Side-Step Lunge*, without finding any significant differences between these practices [21]. Other works have compared exercises such as the *Step Down*, *Unilateral Squat*, *Front Step-Up* and *Forward Lunge* under different conditions: box height [22], external load intensity [23], use of implements [24] or different motion ranges [9]. However, in most of these studies, the exercises were conducted with the body's own weight [25] and without any differentiation between the phases of execution regarding concentric vs. eccentric movement.

In this line, in most studies that have currently been conducted, the sEMG signal has been evaluated by execution cycle of the exercise, that is, by integrating the concentric and eccentric phases in a single unit of analysis [26–28]. However, there is a lack of studies that analyze the EMG activity by differentiating the concentric phase and the eccentric phase to determine at what moment of the exercise a higher level of muscle activation is required [9]. This analysis would allow for more data to be gathered on muscular behavior during exercise to make the best decisions regarding the inclusion of these exercises during training [29].

Nevertheless, more research is still needed to evaluate EMG activity in unilateral lower-body exercises [30,31]. In addition, no studies have been conducted on the performance of the *Monopodal Squat*, *Forward Lunge* and *Lateral Step-Up* exercises to determine which muscle groups have greater EMG activity or to determine their EMG activity in the concentric and the eccentric phases.

Therefore, the objectives of the present study were to evaluate the EMG activity of the gluteus medius, gluteus maximus, biceps femoris, vastus lateralis, vastus medialis and rectus femoris muscles in the *Lateral Step-Up*, *Forward Lunge* and *Monopodal Squat exercises* to: 1) determine which of these three exercises produces greater EMG activity in the evaluated muscle groups; 2) compare the EMG activity of the concentric phase vs. the eccentric phase; and 3) analyze which muscles have the greatest EMG activity in each of the evaluated exercises.

## Materials and methods

### Participants

A total of 20 physically active participants (10 men and 10 women), voluntarily participated in the study. The characteristics of the sample are shown in Table 1.

**Table 1:** Descriptive characteristics of sample. Mean (standard deviation).

	Mean (SD)
Age (years)	24.00 (5.55)
Body mass (kg)	70.40 (16.34)
Height (m)	1.69 (0.10)
BMI (kg·m^-2^)	24.10 (3.22)
5RM Lateral Step-Up (kg)	56.00 (17.96)
5RM Forward Lunge (kg)	58.50 (13.28)
5RM Monopodal Squat (kg)	54.25 (15.58)

The inclusion criteria were a minimum of 6 months of experience in gym training and specifically in performing the exercises that we evaluated in the present study; no musculoskeletal disease or injury in the six months prior to the evaluations, nor any discomfort that prevented or limited the participants in the execution of the exercises to be evaluated. In addition, the participants were instructed not to engage in strenuous physical activity during the 24 hours prior to the evaluation. Those volunteers who, according to the researcher's criteria, performed the exercises with a poor technique were excluded from the study. After explaining the study procedure, all participants signed an informed consent form. This study was approved by the Bioethics Committee of the University of Almería, Spain. The individual in this manuscript has given written informed consent (as outlined in PLOS consent form) to publish these case details.

### Procedures

All participants performed two exercise sessions that were 48 h apart, thus avoiding the effects of muscle fatigue [27]. The first session was focused on familiarization with the exercises and the determination of 5 repetitions maximum (5 RM). In the second session, the EMG activity was evaluated in the *Lateral Step-Up*, *Forward Lunge* and *Monopodal Squat* exercises. These exercises were measured in randomized order.

### Session 1: Familiarization with the *Lateral Step-Up*, *Forward Lunge* and *Monopodal Squat exercises* and the determination of 5 RM

This session began by recording the size of each participant with a Seca height rod (Seca, Hamburg, Germany) and their body weights with a TANITA scale (model BF-350, Tanita, Tokyo, Japan).

Subsequently, each participant performed a warm-up exercise that consisted of 10 minutes of cardiovascular exercise on a treadmill (SALTER RS-30, Salter SA, Barcelona, Spain) at an intensity between 40 and 60% of the maximum reserve heart rate. The subjects were fitted with a chest HR transmitter and wrist monitor recorder, using an individual Polar RS400 (Polar^®^ Vantage NV, Polar Electro Oy, Finland). Maximum HR was predicted from the 220 –age formula [32]. Later, the percentage of heart rate reserve (%HRR) was calculated for each subject. Heart rate reserve (HRR) was determined by predicted maximum HR minus resting HR. The HRR percentage was determined by (exercise HR–resting HR) X 0.4 or 0.6 [32]. Then, several exercises for joint mobility and dynamic-active stretching of lower extremity muscles were performed. After the warm-up there was 3 minutes of recovery. In no case did warm-up cause fatigue in the participants.

Next, each participant performed 3 to 4 sets to reach the final load of 5 repetitions maximum, which is defined as the maximum load that can be lifted only 5 times (5 RM) while maintaining the correct technique for the exercise that is performed [33]. The 5 RM of the *Lateral Step-Up*, *Forward Lunge* and *Monopodal Squat exercises* were randomly calculated for each participant. The resting period between each set and exercise was 3 to 5 minutes to avoid possible muscle fatigue [33,34].

Then, the participants were familiarized with the study procedure for each of the evaluated exercises. For this, the participants performed at least 3 sets between 20% and 40% of 5 of the predetermined RMs until the researchers were satisfied with the technique and the participants felt comfortable and confident with the technique and execution of the exercises. The participants rested from 1.5 to 3 minutes between sets and exercises.

### Session 2. Recording the sEMG data

Participants were required to avoid physical exercise or intense activities for at least 48 hours before this evaluation session [35]. First, an identical warm-up to that of the first session was conducted. The skin was prepared by shaving the adhesion areas of the electrodes and cleaning it with 96% isopropyl alcohol and cotton. Subsequently, we proceeded to place the bipolar sEMG Ag/AgCl electrodes (Medico Lead-Lok, Noida, India), on the dominant side of the participant, following the protocol as described by the European Project "Surface ElectroMyoGraphy for the Non-Invasive Assessment of Muscles" (SENIAM: http://www.seniam.org). Then, we proceeded to the EMG activity evaluation of 6 thigh muscles (gluteus medius -GMed-, gluteus maximus -GMax-, biceps femoris -BF-, vastus lateralis -VL-, vastus medialis -VM- and rectus femoris -RF-) in the *Lateral Step-Up*, *Forward Lunge* and *Monopodal Squat* exercises with a load of 60% of 5 RM.

The electrodes were placed at a distance of 2 cm apart in a longitudinal orientation in relation to the muscle belly fibers. The neutral electrode was placed outside the muscle belly of the evaluated muscle, following the manufacturer's instructions. More specifically, for GMed, the electrodes were placed at a distance of 50% between the iliac crest and the greater trochanter [36]. For the Gmax measurement, the electrodes were placed in the muscular belly at a distance of 50% between the lateral edge of the sacrum and the posterosuperior edge of the greater trochanter [37]. For the BF, the electrodes were placed at 50% of the distance between the line forming the ischial tuberosity and the lateral epicondyle of the tibia [38]. For the VL, the electrodes were placed at two-thirds of the distance between the superior anterior iliac spine and the lateral side of the patella [35]. Regarding the VM, the electrodes were placed at 80% of the distance between the superior anterior iliac spine and the joint space at the anterior border of the medial collateral ligament [35]. Finally, for the RF the electrodes were placed in the middle of the anterior superior iliac spine line and the upper part of the patella [39]. All of the electrodes were covered with an elastic bandage to prevent their possible displacement during exercise.

### Maximum Voluntary Contraction (MVIC)

To normalize and compare the EMG activity of the different muscles among the 3 exercises that were evaluated, the sEMG signal of the maximum voluntary contractions (MVIC) was recorded [40] in the functional actions for the following muscles: for GMed, a hip abduction was performed in the lateral decubitus [37]. For GMax, a hip extension was performed in prone decubitus along with a glute contraction without external resistance [41]. For BF, a knee flexion was performed in prone decubitus and maintained at 45º [42]. For the VL and RF, a knee extension was performed at 45º while sedentary and the column was aligned [23]; finally, for the VM: a knee extension was performed, at 75º while sedentary and with the column aligned [23].

In all cases, the maximum manual resistance was opposed by the researcher in the opposite direction to the muscular motion. Each participant performed 2 MVICs per muscle for 2 repetitions of 5 seconds at the maximum isometric contraction for each repetition [43]. The exercises and the different MVICs were randomly evaluated. The intraclass correlation coefficient (ICC) was higher than 0.97, showing a high reliability in all of the MVICs that were evaluated.

### Experimental trials testing procedure

After a resting period of 10 minutes between the MVIC recordings, we proceeded to randomly perform the following exercises for the recording of the muscle activation in the 6 muscles described above (GMed, GMax, BF, VL, VM and RM) during concentric and eccentric phases: *Lateral Step-Up*, *Forward Lunge* and *Monopodal Squat*. A set of 5 repetitions at 60% of 5 RM was performed for each exercise at a rate of 60 bpm (2 bpm for the concentric phase and 2 bpm for the eccentric phase) using a KORG MA-1 metronome (Keio Electronic Laboratories, Tokyo, Japan). A break of 3–5 minutes was granted between exercises [44]. The ICCs in the sEMG signal between each repetition were higher than 0.94 for all muscles and for all of the exercises that were evaluated.

In all exercises, we used a 20 kg Olympic bar that was 220 cm in length and had a grip diameter of 28 mm (AZAFIT A017-1, Viseu, Portugal), and we used disks that weighed between 1.25 kg and 20 kg (AZAFIT bumper plates, Viseu, Portugal). The bar was placed on the shoulders and trapezium (upper fibers), with a hand grip that was greater than the shoulder width and using the rack as starting support.

#### *Lateral step-up* (Fig 1A)

We instructed the participants to, starting from the standing position on the side of the 40 cm high box (AZAFIT plyo box, Viseu, Portugal), step on the box laterally, always leaving a free space to support both feet while on the box, as well as on the ground for the descent. The knee of the dominant limb should be extended when the nondominant foot is rested on the box.

**Fig 1:**
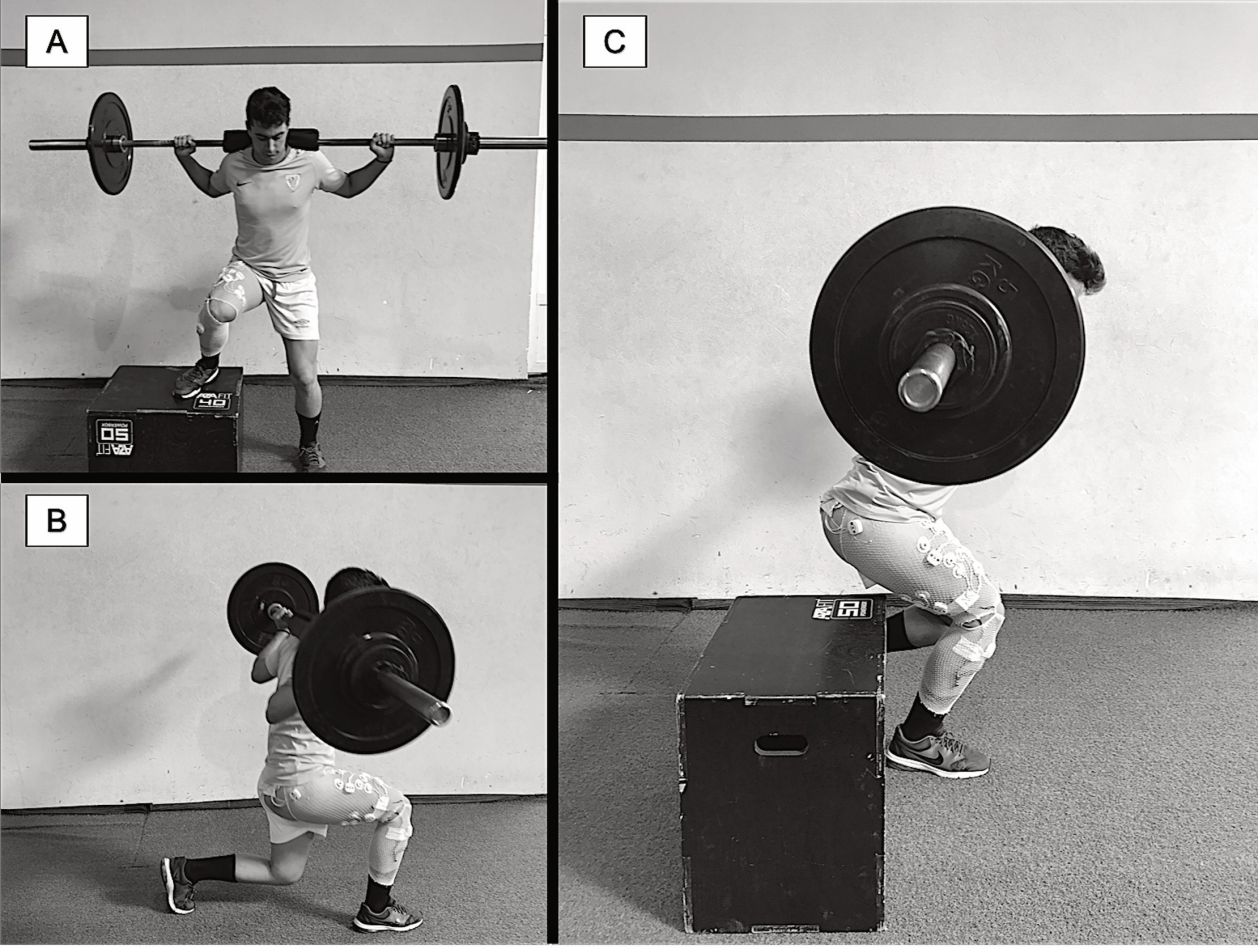
Exercises that were evaluated in the *Lateral Step-Up* (A), *Forward Lunge* (B), and *Monopodal Squat* (C) exercises.

#### *Forward lunge* (Fig 1B)

The exercise consisted of performing a front stride, always advancing with the dominant limb, until the knee was flexed at 90º and preventing the knee of the other (backward) extremity from touching the ground. Then, the initial standing position was reset, and the feet were placed parallel at the final moment of the execution. The stride distance was marked on the ground (as a reference) and corresponded to the natural distance of a step and a half for each participant.

#### *Monopodal squat* (Fig 1C)

The exercise consisted of performing a 75º knee flexion of the dominant limb (in the eccentric phase) with its corresponding extension (in the concentric phase), keeping the nondominant limb aloft without resting it on the ground. The participant flexed his/her knee to rub a box against their buttocks at an individualized height to reach the knee flexion at the predetermined 75° angle. The box was placed behind the participant for safety reasons in the event that they lost their balance. At no time did the participant lean on said box. Once the established knee flexion was reached, the knee of the supporting limb was again extended until the knee was fully extended (avoiding its blockage), without touching the floor with the other limb that was maintained in suspension.

### Superficial electromyography (sEMG)

A WBA 8-channel EMG system (Mega Electronics, Kuopia, Finland) was used for the electromyographic recording. The sEMG signal was recorded through the surface electrodes of the bipolar EMG Ag/AgCl (Medico Lead-Lok, Noida, India) and sent wirelessly at 1000 Hz to the MegaWin software program (Mega Electronics; Kuopio, Finland) for further analysis.

Once the (raw) EMG signal was recorded for each evaluated muscle, it was normalized through root mean square (RMS) transformation for further treatment and calculation of the variables [45]. To determine the MVIC of each muscle, the maximum peak was calculated in microvolts (mV), which were recorded at intervals of 1 second, in the 2 repetitions of the maximum isometric contractions that were performed [46]. To determine and extract the values of the sEMG signal from each repetition per muscle, the knee flexion-extension values of the dominant limb were used as the starting and ending reference for each repetition and were recorded by an electrogoniometer (Biometrics Ltd., Newport, United Kingdom) that was connected and synchronized to the Mega WBA EMG console (Mega Electronics; Kuopia, Finland).

### Statistical analysis

First, the data distribution was analyzed using the Shapiro-Wilk normality test. Since all of the variables followed a normal distribution, the different statistics were evaluated based on parametric tests.

For the analysis of the results, a descriptive statistic of each dependent variable was obtained, and the mean values and standard deviations were extracted. The relative reliability of the measurements was calculated by the ICC with a 95% confidence interval using the one-way random effects model.

Our dependent variable EMG activity data then were analyzed using two separated ANOVAs. A design 3 x 6 ANOVA (exercise*muscle) was applied to determine differences in the EMG activity (% MAVIC) among exercises, and among muscles in each exercise; and a design ANOVA 3 x 6 x 2 (exercise*muscle*contraction type) was applied to determine differences in the EMG activity according to different contraction types (concentric and eccentric) in each exercise. In addition, to assess assumptions of variance, Mauchly’s test of sphericity was performed using all the ANOVA results. A Greenhouse–Geisser correction was performed to adjust the degrees of freedom if an assumption was violated, while pairwise comparisons using a Bonferroni adjustment were employed if a significant main effect was observed. We also calculate effect sizes (ES) for each ANOVA by using partial eta-squared (ηp^2^); 0.2, 0.5, 0.8, and 1.3 were set as lower thresholds for “small”, “medium”, “large”, and “very large” ES, respectively [47].

Statistical analyses were carried out using the IBM SPSS software (v.26), and the level of significance was set at *p* < 0.05.

## Results

**Fig 2:**
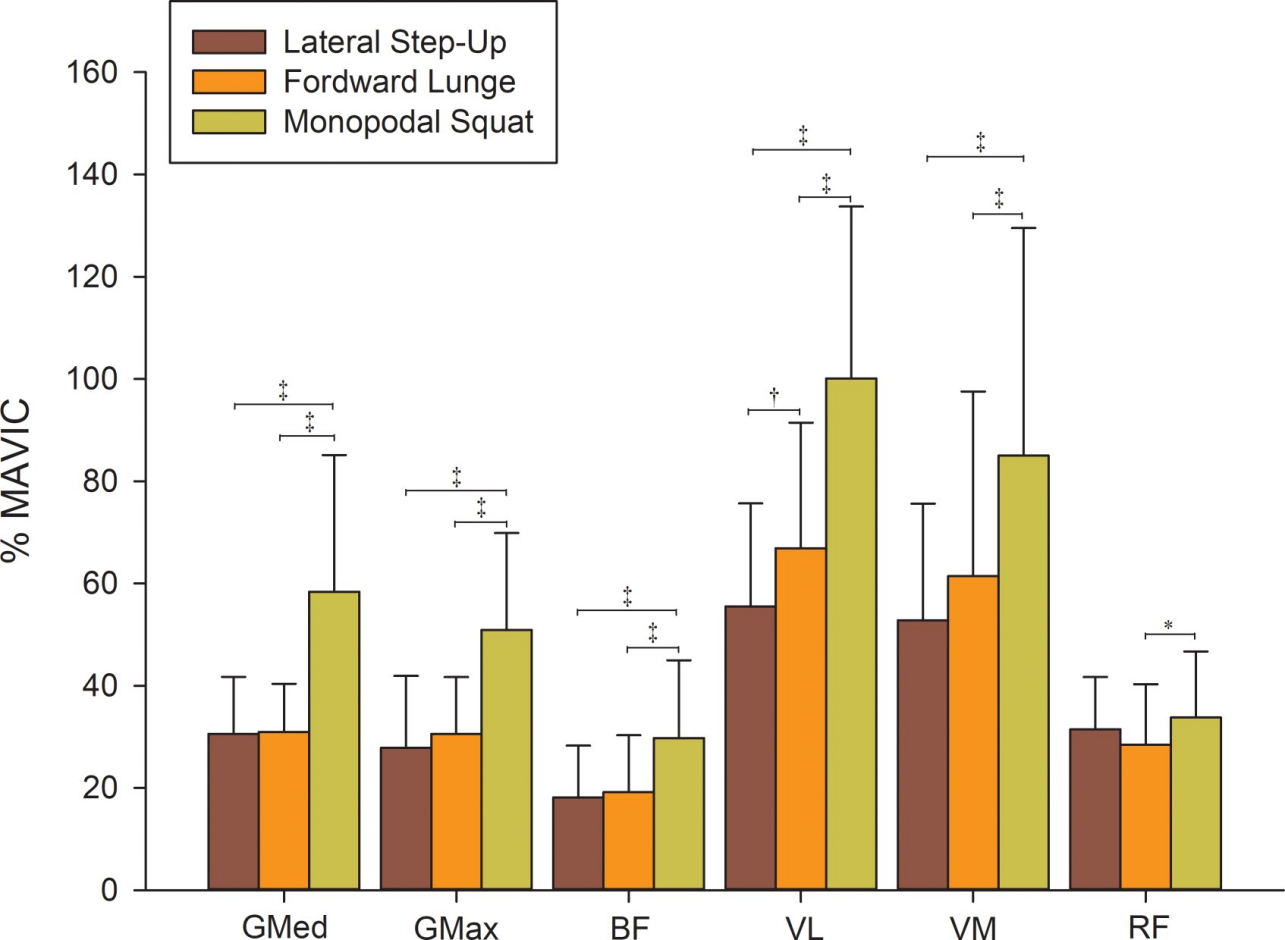
Comparison of the EMG activity between each of the exercises and muscles that were evaluated (expressed in MVIC %). Gluteus medius; gluteus maximus; biceps femoris; vastus lateralis; vastus medialis; rectus femoris. * *p* ≤ 0.05; † *p* ≤ 0.01; ‡ *p* ≤ 0.001.

Statistical analysis revealed that the main effect of exercise on EMG activity were significant with a medium effect size (F_(4.86, 92.35)_ = 13.28, *p* < 0.001, ηp^2^ = 0.41). The *Monopodal Squat* exercise showed a higher EMG activity (*p* ≤ 0.001) relative to the *Lateral Step-Up* and *Forward Lunge* exercises in all of the evaluated muscles except for the rectus femoris, which showed a significantly higher EMG activity only with the *Forward Lunge* exercise (Fig 2).

Tables 2–4 show comparison of the EMG activity (expressed in mV) during the concentric and eccentric phase in the three evaluated exercises (*Lateral Step-Up*, *Forward Lunge*, and *Monopodal Squat*). ANOVA showed significant main effects for exercise (F_(2, 38)_ = 63.95, *p* < 0.001, ηp^2^ = 0.77), muscle (F_(3.03, 57.6)_ = 35.41, *p* < 0.001, ηp^2^ = 0.65), contraction type (F_(1, 19)_ = 67.47, *p* < 0.001, ηp^2^ = 0.78), and for exercise*muscles (F_(4.84, 92.11)_ = 11.61, *p* < 0.001, ηp^2^ = 0.37), exercise*contraction type (F_(2, 38)_ = 25.67, *p* < 0.001, ηp^2^ = 0.57), muscle*contraction type (F_(2.75, 52.39)_ = 4.30, *p* < 0.01, ηp^2^ = 0.18), and exercise*muscle*contraction type (F_(4.17, 79.39)_ = 7.77, *p* < 0.001, ηp^2^ = 0.29). In all exercises (*Lateral Step-Up*, *Forward Lunge*, and *Monopodal Squat)* the EMG activity was significantly higher (*p* < 0.01) in the concentric phase that in the eccentric phase for all evaluated muscles (Gluteus medius and maximus, biceps femoris, vastus lateralis and medialis, and rectus femoris) (Tables 2–4).

**Table 2:** Comparison of the EMG activity (expressed in mV) during the concentric and eccentric phase in the *Lateral Step-Up* exercise.

	Mean ± SD		P-value
	Concentric	Eccentric	
Gluteus medius	117.17 ± 57.11	60.59 ± 22.27	< 0.001
Gluteus maximus	97.61 ± 50.43	42.99 ± 25.89	< 0.001
Biceps femoris	92.17 ± 28.24	51.85 ± 14.91	0.009
Vastus lateralis	197.34 ± 66.55	111.73 ± 32.70	< 0.001
Vastus medialis	216.71 ± 75.22	124.13 ± 34.06	< 0.001
Rectus femoris	198.00 ± 58.86	118.27 ± 35.25	< 0.001

**Table 3:** Comparison of EMG activity (expressed in mV) during the concentric and eccentric phases in the *Forward Lunge* exercise.

	Mean ± SD		P-value
	Concentric	Eccentric	
Gluteus medius	106.25 ± 34.47	71.59 ± 19.22	< 0.001
Gluteus maximus	106.09 ± 51.36	55.02 ± 19.46	< 0.001
Biceps femoris	88.77 ± 29.89	62.92 ± 23.94	< 0.001
Vastus lateralis	208.18 ± 73.09	164.73 ± 48.89	< 0.001
Vastus medialis	206.82 ± 84.51	175.50 ± 62.77	0.012
Rectus femoris	148.39 ± 42.69	128.23 ± 34.44	0.005

**Table 4:** Comparison of the EMG activity (expressed in mV) during the concentric and eccentric phases in the *Monopodal Squat* exercise.

	Mean ± SD		P-value
	Concentric	Eccentric	
Gluteus medius	194.68 ± 90.57	141.07 ± 63.28	< 0.001
Gluteus maximus	168.68 ± 85.45	102.39 ± 39.25	< 0.001
Biceps femoris	138.29 ± 46.57	101.54 ± 29.63	0.007
Vastus lateralis	314.81 ± 122.18	261.87 ± 99.17	< 0.001
Vastus medialis	288.30 ± 118.18	257.90 ± 103.11	0.005
Rectus femoris	178.14 ± 68.67	161.50 ± 57.64	0.019

**Fig 3:**
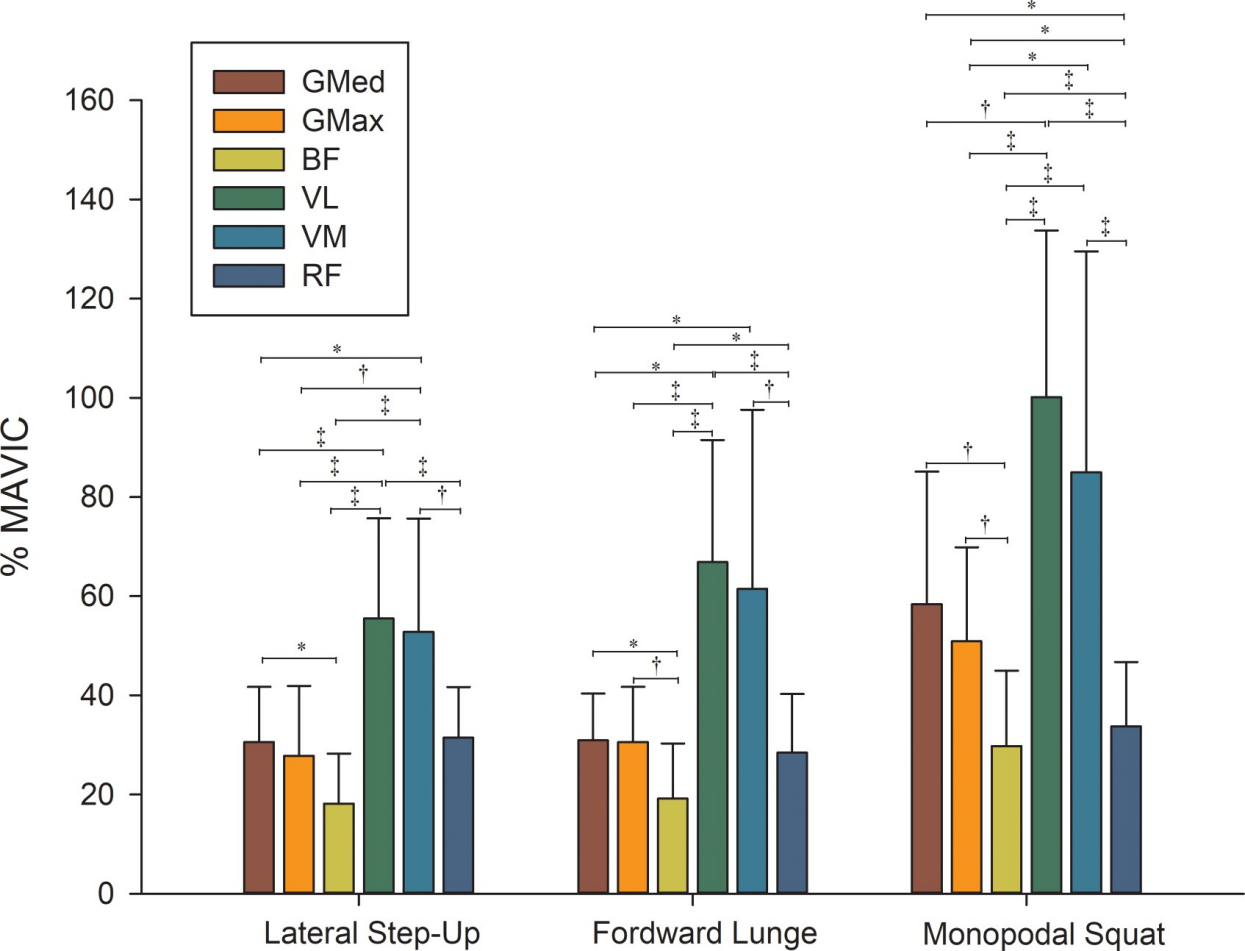
Mean electromyography activity normalized to maximal voluntary isometric contraction (MVIC) during the *Lateral Step-Up*, *Forward Lunge* and *Monopodal Squat* exercise. The rectangular bars represent means, and the error bars represent standard deviations. * *p* < 0.05; † *p* < 0.01; ‡ *p* < 0.001.

Regarding the EMG activity in each exercise, the vastus lateralis and medialis showed the highest muscle activation, followed for gluteus medius and maximus and, finally, for the rectus femoris. These results were consistent in the three evaluated exercises (Fig 3).

## Discussion

sEMG is an established way to quantify muscle activity [25]. In this sense, understanding the relative differences in EMG activity could assist trainers, clinicians and/or physiotherapists in incorporating lower limbs exercises into trainings and/or treatment and prevention programs based on the level of muscle activity regarding to lower limb muscles. Thus, the objectives of the present study were to evaluate the EMG of the GMed, GMax, BF, VL, VM and RF muscles in the *Lateral Step-Up*, *Forward Lunge* and *Monopodal Squat* exercises to: 1) determine which exercise produces the highest EMG activity in the muscles that were evaluated; 2) compare the EMG activity of the concentric phase vs. the eccentric phase; and 3) analyze which muscles have the greatest EMG activity in each exercise.

Regarding the first objective, note that *Monopodal Squat* showed a significantly higher EMG activity in all of the muscles that were evaluated in relation to the *Lateral Step-Up* and *Forward Lunge* exercises. Likewise, Ayotte et al. [22] found a greater EMG activity in the GMax, BF and VM in the *Mini-squat* exercise (one leg) compared to *Lateral Step-Up*. However, the differences in EMG activity that were found by these authors were lower in comparison to those of the present study, possibly because these authors used lower loads (the participants’ body weights) as well as lower boxes (15.24 cm), compared to the present study. Most recently, Haltfield et al. [48] also found greater activation in the VL and VM, followed by the GM, RF and BF when comparing *Single-leg squat* with *Step-down*, *Half step-down* and *Step-up*. This shows that *Squats*, which are performed on one leg, are an adequate exercise primarily for the activation of the extensor and stabilizing muscles of the knee as well as the stabilizers of the hip.

Another finding of the present study was the higher EMG activity that was found in the concentric phase of the execution, compared to the eccentric phase. These results are in accordance with those of previous studies on muscle activity in exercises with motion patterns that were similar to those performed in the present study, such as *Parallel back squats* and *Overhead squats* [49]; *Bilateral squat*, *Rear leg elevated split squat* and *Split squat* [31]; or *traditional Squat* with *chain* and *elastic* bands [44].

In this sense, some authors indicate that the higher EMG activity during the concentric phase may be due to the lower speed of muscle fiber conduction during eccentric actions compared to concentric actions [13]. In addition, normally the angular speed at which the eccentric phase is executed is lower than that of the concentric phase to maintain an adequate execution technique and prevent possible musculotendinous lesions [50]. Therefore, this would result in a lower EMG activity in the eccentric phase of the exercise.

Another objective of the present study was to analyze which muscles have higher EMG activity in each exercise. In this sense, we found a similar pattern in the EMG activity that was recorded for each muscle in the three evaluated exercises. Regarding the *Lateral Step-Up*, we found a significantly higher EMG activity in the VL and VM in relation to the rest of the muscle groups. A similar activation of the GMed and RF was also observed. As in the present study, MacAskill et al. [51]observed greater EMG activity of the GMed compared to the GMax during *Lateral Step-Up*. Although these authors used no external loads for this exercise, they argued that *Step-Up* could be a suitable exercise for strengthening the GMed and GMax muscles if an additional external load was applied, as in the case of the present study.

Regarding *Forward Lunge*, we observed a significantly higher EMG activity in the VL and VM, and this was followed by the GMed and GMax, the RF, and finally the BF. Consistent with our study, Krause et al. [52]found similar results. However, these authors did not evaluate the VL or VM. Stastny et al. [53] compared the EMG activity between *Walking lunge* (WL) and *Split squat* (SSq). Both exercises have similar kinematics, although the biggest difference between them is the dynamic nature of the WL in relation to the static SSq, where both feet are kept resting on the ground, one in front of the other. In this sense, note that the *Forward Lunge* exercise that was evaluated in this study is a hybrid of the two previous (WL and SSQ) as it is performed by alternating both feet without performing an anterior displacement (*walking*). Our results are more in agreement with the pattern of EMG activity that was observed in the SSq exercise of the study by Stastny et al. [53], where the VL and VM had the highest EMG activity, followed by the GMed and BF. In contrast, in the WL exercise, the GMed and VM had the greatest EMG activity in relation to the rest of the muscle groups. According to the authors, this was due to the forces that impact walking that increase the GMed activity. However, despite the differences that were found between both exercises, these authors concluded that the WL and SSq should be used in strengthening programs and that the WL should primarily be used when the GMed and MV are the targets.

Regarding *Monopodal Squat* exercise, in the present study we observed a significantly greater activation of the VL and VM compared to the rest of the muscle groups. In addition, the high activation % of the GMed and GMax (approximately 60% MVIC) were notable. The high activity of the gluteal muscles could possibly be due to pelvic and knee stabilization [54], since it is a monopodial exercise and is therefore more unstable than a bipodal execution. In fact, although *Monopodial Squat*s do not have an external load, they are frequently performed for rehabilitation purposes [55]. Similarly, McCurdy et al. [56] found a significantly higher EMG activity in the GMed when performing *Squats* on one leg than on two. According to the authors, this was due to the control of the knee valgus. As in the present study, these authors also found greater EMG activity in the quadriceps compared to hamstrings [56]. As in the present study, Ayotte et al. [22] found that for *Mini-squats* (one leg), the greatest EMG activity was recorded in the VM, followed by the GMax, GMed and BF. Bolgla et al. [55] recorded similar results when analyzing *Mini-squats* (one leg), finding the highest EMG activity in the VM, followed by GMed and GMax.

One of the main limitations of the present study was the load intensity that we used to record the EMG activity (60% 5 RM). We found no study that specifically used this load, since the literature features a great deal of heterogeneity regarding the loads that are used in the studies of sEMG. Most of the studies on unilateral exercises usually use the participant's own body weight because they are oriented toward rehabilitation purposes [22,48,51,55,57]. Deforest et al. [31] used 85% 1 RM to analyze *Double-leg squats* and 50% of said load for *Single-leg squats*. McCurdy et al. [16] used 85% 3 RM to compare the EMG activity between *2-leg squats* and *Modified single-leg* squats. Stastny et al. [53] used 5 RM to analyze the EMG activity during *Split squats* and *Walking lunges*. The fact that each study uses different load intensities meets a methodological and procedural need to achieve the research objectives in the most effective and efficient possible way while controlling for any variable that could alter the results. In this sense, the load that was selected in the present study was determined to preserve the safety of the participants while maintaining an adequate execution technique; additionally, we considered an sEMG signal threshold that would be sufficiently broad to recommend the selected exercises and loads strength-gaining purposes (between 40–60% MVIC) [22,58,59].

## Conclusions

*Monopodal Squat* produces significantly higher EMG activity in the GMed, GMax, BF, VL, VM and RF muscles compared to the *Lateral Step-Up* and *Forward Lunge*, except for the RF in the *Lateral Step-Up*. In the three evaluated exercises, the concentric phase produces a significantly higher EMG activity in all of the evaluated muscles in relation to the eccentric phase. The VL, VM, were the muscles with the highest EMG activity in all three of the exercises that were evaluated, followed by GMed and GMax. Therefore, *Monopodal Squat*, *Lateral Step-Up* and *Forward Lunge* exercises are recommended not only for rehabilitation purposes but also for performance objectives and strength improvement in the lower limbs.

## Supporting information

S1 File (HTM)

S2 File (HTM)
