# Supplementary material for: Electromyographic activity in the gluteus medius, gluteus maximus, biceps femoris, vastus lateralis, vastus medialis and rectus femoris during the Monopodal Squat, Forward Lunge and Lateral Step-Up exercises
Source: PLoS One. 2020 Apr 1;15(4):e0230841. doi: 10.1371/journal.pone.0230841 (PMC7112217; doi:10.1371/journal.pone.0230841)
Supplement: S1 File — (HTM) [file pone.0230841.s001.htm]

xml version="1.0" encoding="UTF-8"?


OUTPUT


|  |  |  |
| --- | --- | --- |
| IBM SPSS Informe web - ResultadoANOVArep3x6x2.spv     ---   Contenido  Anterior  Siguiente  Ayuda |  | Controles inhabilitados por el sistema     --- |

- Registro

  - Registro
- Modelo lineal general

  - Notas
- Modelo lineal general

  - Notas
  - Advertencias
  - Factores intra-sujetos
  - Estadísticos descriptivos
  - Pruebas multivariante
  - Prueba de esfericidad de Mauchly
  - Pruebas de efectos intra-sujetos
  - Pruebas de contrastes intra-sujetos
  - Pruebas de efectos inter-sujetos
  - Medias marginales estimadas

    - 1. Ejerc

      - Estimaciones
      - Comparaciones por parejas
      - Pruebas multivariante
    - 2. Muscu

      - Estimaciones
      - Comparaciones por parejas
      - Pruebas multivariante
    - 3. act

      - Estimaciones
      - Comparaciones por parejas
      - Pruebas multivariante
    - 4. Ejerc \* Muscu

      - Estimaciones
      - Comparaciones por parejas
      - Pruebas multivariante
      - Comparaciones por parejas
      - Pruebas multivariante
    - 5. Ejerc \* act

      - Estimaciones
      - Comparaciones por parejas
      - Pruebas multivariante
      - Comparaciones por parejas
      - Pruebas multivariante
    - 6. Muscu \* act

      - Estimaciones
      - Comparaciones por parejas
      - Pruebas multivariante
      - Comparaciones por parejas
      - Pruebas multivariante
    - 7. Ejerc \* Muscu \* act

      - Estimaciones
      - Comparaciones por parejas
      - Pruebas multivariante
      - Comparaciones por parejas
      - Pruebas multivariante
      - Comparaciones por parejas
      - Pruebas multivariante

- Suprimir

Registro  
Registro - Registro - febrero 24, 2020

GLM STEP\_UP\_MEDIA5REP\_uV\_GMED\_CONCEN STEP\_UP\_MEDIA5REP\_uV\_GMED\_EXCEN  
    STEP\_UP\_MEDIA5REP\_uV\_GMAX\_CONCEN STEP\_UP\_MEDIA5REP\_uV\_GMAX\_EXCEN STEP\_UP\_MEDIA5REP\_uV\_BF\_CONCEN  
    STEP\_UP\_MEDIA5REP\_uV\_BF\_EXCEN STEP\_UP\_MEDIA5REP\_uV\_VL\_CONCEN STEP\_UP\_MEDIA5REP\_uV\_VL\_EXCEN  
    STEP\_UP\_MEDIA5REP\_uV\_VM\_CONCEN STEP\_UP\_MEDIA5REP\_uV\_VM\_EXCEN STEP\_UP\_MEDIA5REP\_uV\_RF\_CONCEN  
    STEP\_UP\_MEDIA5REP\_uV\_RF\_EXCEN LUNGE\_MEDIA5REP\_uV\_GMED\_CONCEN LUNGE\_MEDIA5REP\_uV\_GMED\_EXCEN  
    LUNGE\_MEDIA5REP\_uV\_GMAX\_CONCEN LUNGE\_MEDIA5REP\_uV\_GMAX\_EXCEN LUNGE\_MEDIA5REP\_uV\_BF\_CONCEN  
    LUNGE\_MEDIA5REP\_uV\_BF\_EXCEN LUNGE\_MEDIA5REP\_uV\_VL\_CONCEN LUNGE\_MEDIA5REP\_uV\_VL\_EXCEN  
    LUNGE\_MEDIA5REP\_uV\_VM\_CONCEN LUNGE\_MEDIA5REP\_uV\_VM\_EXCEN LUNGE\_MEDIA5REP\_uV\_RF\_CONCEN  
    LUNGE\_MEDIA5REP\_uV\_RF\_EXCEN SQ\_MEDIA5REP\_uV\_GMED\_CONCEN SQ\_MEDIA5REP\_uV\_GMED\_EXCEN  
    SQ\_MEDIA5REP\_uV\_GMAX\_CONCEN SQ\_MEDIA5REP\_uV\_GMAX\_EXCEN SQ\_MEDIA5REP\_uV\_BF\_CONCEN  
    SQ\_MEDIA5REP\_uV\_BF\_EXCEN SQ\_MEDIA5REP\_uV\_VL\_CONCEN SQ\_MEDIA5REP\_uV\_VL\_EXCEN  
    SQ\_MEDIA5REP\_uV\_VM\_CONCEN SQ\_MEDIA5REP\_uV\_VM\_EXCEN SQ\_MEDIA5REP\_uV\_RF\_CONCEN  
    SQ\_MEDIA5REP\_uV\_RF\_EXCEN  
  /WSFACTOR=Ejerc 3 Polynomial Muscu 6 Polynomial act 2 Polynomial  
  /METHOD=SSTYPE(3)  
  /EMMEANS=TABLES(Ejerc) COMPARE ADJ(BONFERRONI)  
  /EMMEANS=TABLES(Muscu) COMPARE ADJ(BONFERRONI)  
  /EMMEANS=TABLES(act) COMPARE ADJ(BONFERRONI)  
  /EMMEANS=TABLES(Ejerc\*Muscu) COMPARE(Muscu)ADJ(BONFERRONI)  
  /EMMEANS=TABLES(Ejerc\*act) COMPARE(act)ADJ(BONFERRONI)  
  /EMMEANS=TABLES(Muscu\*act) COMPARE(act)ADJ(BONFERRONI)  
  /EMMEANS=TABLES(Ejerc\*Muscu\*act) COMPARE(Ejerc\*Muscu\*act)ADJ(BONFERRONI)  
  /PRINT=DESCRIPTIVE ETASQ OPOWER HOMOGENEITY  
  /CRITERIA=ALPHA(.05)  
  /WSDESIGN=Ejerc Muscu act Ejerc\*Muscu Ejerc\*act Muscu\*act Ejerc\*Muscu\*act.

Modelo lineal general

NotasNotas, tabla, 0 niveles de cabeceras de columna y 2 niveles de cabeceras de fila, tabla con 3 columnas y 12 filas

|  |  |  |
| --- | --- | --- |
| Salida creada | | 20-FEB-2020 19:16:07 |
| Comentarios | |  |
| Entrada | Datos | C:\Users\User\Desktop\BASE\_DATOS\_TFM\_ISA\_PlosOne.sav |
|  | Conjunto de datos activo | ConjuntoDatos1 |
|  | Filtro | <ninguno> |
|  | Ponderación | <ninguno> |
|  | Segmentar archivo | <ninguno> |
|  | N de filas en el archivo de datos de trabajo | 20 |
| Sintaxis | | GLM STEP\_UP\_MEDIA5REP\_uV\_GMED\_CONCEN STEP\_UP\_MEDIA5REP\_uV\_GMED\_EXCEN  STEP\_UP\_MEDIA5REP\_uV\_GMAX\_CONCEN STEP\_UP\_MEDIA5REP\_uV\_GMAX\_EXCEN STEP\_UP\_MEDIA5REP\_uV\_BF\_CONCEN  STEP\_UP\_MEDIA5REP\_uV\_BF\_EXCEN STEP\_UP\_MEDIA5REP\_uV\_VL\_CONCEN STEP\_UP\_MEDIA5REP\_uV\_VL\_EXCEN  STEP\_UP\_MEDIA5REP\_uV\_VM\_CONCEN STEP\_UP\_MEDIA5REP\_uV\_VM\_EXCEN STEP\_UP\_MEDIA5REP\_uV\_RF\_CONCEN  STEP\_UP\_MEDIA5REP\_uV\_RF\_EXCEN LUNGE\_MEDIA5REP\_uV\_GMED\_CONCEN LUNGE\_MEDIA5REP\_uV\_GMED\_EXCEN  LUNGE\_MEDIA5REP\_uV\_GMAX\_CONCEN LUNGE\_MEDIA5REP\_uV\_GMAX\_EXCEN LUNGE\_MEDIA5REP\_uV\_BF\_CONCEN  LUNGE\_MEDIA5REP\_uV\_BF\_EXCEN LUNGE\_MEDIA5REP\_uV\_VL\_CONCEN LUNGE\_MEDIA5REP\_uV\_VL\_EXCEN  LUNGE\_MEDIA5REP\_uV\_VM\_CONCEN LUNGE\_MEDIA5REP\_uV\_VM\_EXCEN LUNGE\_MEDIA5REP\_uV\_RF\_CONCEN  LUNGE\_MEDIA5REP\_uV\_RF\_EXCEN SQ\_MEDIA5REP\_uV\_GMED\_CONCEN SQ\_MEDIA5REP\_uV\_GMED\_EXCEN  SQ\_MEDIA5REP\_uV\_GMAX\_CONCEN SQ\_MEDIA5REP\_uV\_GMAX\_EXCEN SQ\_MEDIA5REP\_uV\_BF\_CONCEN  SQ\_MEDIA5REP\_uV\_BF\_EXCEN SQ\_MEDIA5REP\_uV\_VL\_CONCEN SQ\_MEDIA5REP\_uV\_VL\_EXCEN  SQ\_MEDIA5REP\_uV\_VM\_CONCEN SQ\_MEDIA5REP\_uV\_VM\_EXCEN SQ\_MEDIA5REP\_uV\_RF\_CONCEN  SQ\_MEDIA5REP\_uV\_RF\_EXCEN  /WSFACTOR=Ejerc 3 Polynomial Muscu 6 Polynomial act 2 Polynomial  /METHOD=SSTYPE(3)  /EMMEANS=TABLES(Ejerc) COMPARE ADJ(BONFERRONI)  /EMMEANS=TABLES(Muscu) COMPARE ADJ(BONFERRONI)  /EMMEANS=TABLES(act) COMPARE ADJ(BONFERRONI)  /EMMEANS=TABLES(Ejerc\*Muscu) COMPARE(Muscu)ADJ(BONFERRONI)  /EMMEANS=TABLES(Ejerc\*act) COMPARE(act)ADJ(BONFERRONI)  /EMMEANS=TABLES(Muscu\*act) COMPARE(act)ADJ(BONFERRONI)  /EMMEANS=TABLES(Ejerc\*Muscu\*act) COMPARE(Ejerc\*Muscu\*act)ADJ(BONFERRONI)  /PRINT=DESCRIPTIVE ETASQ OPOWER HOMOGENEITY  /CRITERIA=ALPHA(.05)  /WSDESIGN=Ejerc Muscu act Ejerc\*Muscu Ejerc\*act Muscu\*act Ejerc\*Muscu\*act. |
| Recursos | Tiempo de procesador | 00:00:00,02 |
|  | Tiempo transcurrido | 00:00:00,01 |
|  |  |  |

Modelo lineal general

NotasNotas, tabla, 0 niveles de cabeceras de columna y 2 niveles de cabeceras de fila, tabla con 3 columnas y 14 filas

|  |  |  |
| --- | --- | --- |
| Salida creada | | 20-FEB-2020 19:16:27 |
| Comentarios | |  |
| Entrada | Datos | C:\Users\User\Desktop\BASE\_DATOS\_TFM\_ISA\_PlosOne.sav |
|  | Conjunto de datos activo | ConjuntoDatos1 |
|  | Filtro | <ninguno> |
|  | Ponderación | <ninguno> |
|  | Segmentar archivo | <ninguno> |
|  | N de filas en el archivo de datos de trabajo | 20 |
| Manejo de valores perdidos | Definición de perdidos | Los valores perdidos definidos por el usuario se tratan como perdidos. |
|  | Casos utilizados | Las estadísticas se basan en todos los casos con datos válidos para todas las variables del modelo. |
| Sintaxis | | GLM STEP\_UP\_MEDIA5REP\_uV\_GMED\_CONCEN STEP\_UP\_MEDIA5REP\_uV\_GMED\_EXCEN  STEP\_UP\_MEDIA5REP\_uV\_GMAX\_CONCEN STEP\_UP\_MEDIA5REP\_uV\_GMAX\_EXCEN STEP\_UP\_MEDIA5REP\_uV\_BF\_CONCEN  STEP\_UP\_MEDIA5REP\_uV\_BF\_EXCEN STEP\_UP\_MEDIA5REP\_uV\_VL\_CONCEN STEP\_UP\_MEDIA5REP\_uV\_VL\_EXCEN  STEP\_UP\_MEDIA5REP\_uV\_VM\_CONCEN STEP\_UP\_MEDIA5REP\_uV\_VM\_EXCEN STEP\_UP\_MEDIA5REP\_uV\_RF\_CONCEN  STEP\_UP\_MEDIA5REP\_uV\_RF\_EXCEN LUNGE\_MEDIA5REP\_uV\_GMED\_CONCEN LUNGE\_MEDIA5REP\_uV\_GMED\_EXCEN  LUNGE\_MEDIA5REP\_uV\_GMAX\_CONCEN LUNGE\_MEDIA5REP\_uV\_GMAX\_EXCEN LUNGE\_MEDIA5REP\_uV\_BF\_CONCEN  LUNGE\_MEDIA5REP\_uV\_BF\_EXCEN LUNGE\_MEDIA5REP\_uV\_VL\_CONCEN LUNGE\_MEDIA5REP\_uV\_VL\_EXCEN  LUNGE\_MEDIA5REP\_uV\_VM\_CONCEN LUNGE\_MEDIA5REP\_uV\_VM\_EXCEN LUNGE\_MEDIA5REP\_uV\_RF\_CONCEN  LUNGE\_MEDIA5REP\_uV\_RF\_EXCEN SQ\_MEDIA5REP\_uV\_GMED\_CONCEN SQ\_MEDIA5REP\_uV\_GMED\_EXCEN  SQ\_MEDIA5REP\_uV\_GMAX\_CONCEN SQ\_MEDIA5REP\_uV\_GMAX\_EXCEN SQ\_MEDIA5REP\_uV\_BF\_CONCEN  SQ\_MEDIA5REP\_uV\_BF\_EXCEN SQ\_MEDIA5REP\_uV\_VL\_CONCEN SQ\_MEDIA5REP\_uV\_VL\_EXCEN  SQ\_MEDIA5REP\_uV\_VM\_CONCEN SQ\_MEDIA5REP\_uV\_VM\_EXCEN SQ\_MEDIA5REP\_uV\_RF\_CONCEN  SQ\_MEDIA5REP\_uV\_RF\_EXCEN  /WSFACTOR=Ejerc 3 Polynomial Muscu 6 Polynomial act 2 Polynomial  /METHOD=SSTYPE(3)  /EMMEANS=TABLES(Ejerc) COMPARE ADJ(BONFERRONI)  /EMMEANS=TABLES(Muscu) COMPARE ADJ(BONFERRONI)  /EMMEANS=TABLES(act) COMPARE ADJ(BONFERRONI)  /EMMEANS=TABLES(Ejerc\*Muscu) COMPARE(Muscu)ADJ(BONFERRONI)  /EMMEANS=TABLES(Ejerc\*act) COMPARE(act)ADJ(BONFERRONI)  /EMMEANS=TABLES(Muscu\*act) COMPARE(act)ADJ(BONFERRONI)  /EMMEANS=TABLES(Ejerc\*Muscu\*act) COMPARE(act)ADJ(BONFERRONI)  /PRINT=DESCRIPTIVE ETASQ OPOWER HOMOGENEITY  /CRITERIA=ALPHA(.05)  /WSDESIGN=Ejerc Muscu act Ejerc\*Muscu Ejerc\*act Muscu\*act Ejerc\*Muscu\*act. |
| Recursos | Tiempo de procesador | 00:00:00,08 |
|  | Tiempo transcurrido | 00:00:00,07 |
|  |  |  |

Modelo lineal general

AdvertenciasAdvertencias, tabla, 0 niveles de cabeceras de columna y 0 niveles de cabeceras de fila, tabla con 1 columnas y 2 filas

|  |
| --- |
| La especificación HOMOGENEITY en el subcomando PRINT se ignorará porque no hay factores inter-sujetos. |
|  |

Modelo lineal general

Factores intra-sujetosFactores intra-sujetos, tabla, Medida, MEASURE\_1, 1 capas, 1 niveles de cabeceras de columna y 3 niveles de cabeceras de fila, tabla con 4 columnas y 39 filas

| |  |  |  | | --- | --- | --- | | Medida: | MEASURE\_1 | MEASURE\_1 | | | | |
|  |  |  |  |
| --- | --- | --- | --- |
| Ejerc | Muscu | act | Variable dependiente |
| 1 | 1 | 1 | STEP\_UP\_MEDIA5REP\_uV\_GMED\_CONCEN |
|  |  | 2 | STEP\_UP\_MEDIA5REP\_uV\_GMED\_EXCEN |
|  | 2 | 1 | STEP\_UP\_MEDIA5REP\_uV\_GMAX\_CONCEN |
|  |  | 2 | STEP\_UP\_MEDIA5REP\_uV\_GMAX\_EXCEN |
|  | 3 | 1 | STEP\_UP\_MEDIA5REP\_uV\_BF\_CONCEN |
|  |  | 2 | STEP\_UP\_MEDIA5REP\_uV\_BF\_EXCEN |
|  | 4 | 1 | STEP\_UP\_MEDIA5REP\_uV\_VL\_CONCEN |
|  |  | 2 | STEP\_UP\_MEDIA5REP\_uV\_VL\_EXCEN |
|  | 5 | 1 | STEP\_UP\_MEDIA5REP\_uV\_VM\_CONCEN |
|  |  | 2 | STEP\_UP\_MEDIA5REP\_uV\_VM\_EXCEN |
|  | 6 | 1 | STEP\_UP\_MEDIA5REP\_uV\_RF\_CONCEN |
|  |  | 2 | STEP\_UP\_MEDIA5REP\_uV\_RF\_EXCEN |
| 2 | 1 | 1 | LUNGE\_MEDIA5REP\_uV\_GMED\_CONCEN |
|  |  | 2 | LUNGE\_MEDIA5REP\_uV\_GMED\_EXCEN |
|  | 2 | 1 | LUNGE\_MEDIA5REP\_uV\_GMAX\_CONCEN |
|  |  | 2 | LUNGE\_MEDIA5REP\_uV\_GMAX\_EXCEN |
|  | 3 | 1 | LUNGE\_MEDIA5REP\_uV\_BF\_CONCEN |
|  |  | 2 | LUNGE\_MEDIA5REP\_uV\_BF\_EXCEN |
|  | 4 | 1 | LUNGE\_MEDIA5REP\_uV\_VL\_CONCEN |
|  |  | 2 | LUNGE\_MEDIA5REP\_uV\_VL\_EXCEN |
|  | 5 | 1 | LUNGE\_MEDIA5REP\_uV\_VM\_CONCEN |
|  |  | 2 | LUNGE\_MEDIA5REP\_uV\_VM\_EXCEN |
|  | 6 | 1 | LUNGE\_MEDIA5REP\_uV\_RF\_CONCEN |
|  |  | 2 | LUNGE\_MEDIA5REP\_uV\_RF\_EXCEN |
| 3 | 1 | 1 | SQ\_MEDIA5REP\_uV\_GMED\_CONCEN |
|  |  | 2 | SQ\_MEDIA5REP\_uV\_GMED\_EXCEN |
|  | 2 | 1 | SQ\_MEDIA5REP\_uV\_GMAX\_CONCEN |
|  |  | 2 | SQ\_MEDIA5REP\_uV\_GMAX\_EXCEN |
|  | 3 | 1 | SQ\_MEDIA5REP\_uV\_BF\_CONCEN |
|  |  | 2 | SQ\_MEDIA5REP\_uV\_BF\_EXCEN |
|  | 4 | 1 | SQ\_MEDIA5REP\_uV\_VL\_CONCEN |
|  |  | 2 | SQ\_MEDIA5REP\_uV\_VL\_EXCEN |
|  | 5 | 1 | SQ\_MEDIA5REP\_uV\_VM\_CONCEN |
|  |  | 2 | SQ\_MEDIA5REP\_uV\_VM\_EXCEN |
|  | 6 | 1 | SQ\_MEDIA5REP\_uV\_RF\_CONCEN |
|  |  | 2 | SQ\_MEDIA5REP\_uV\_RF\_EXCEN |
|  |  |  |  |

Modelo lineal general

Estadísticos descriptivosEstadísticos descriptivos, tabla, 1 niveles de cabeceras de columna y 1 niveles de cabeceras de fila, tabla con 4 columnas y 38 filas

|  |  |  |  |
| --- | --- | --- | --- |
|  | Media | Desv. Desviación | N |
| STEP\_UP\_MEDIA5REP\_uV\_GMED\_CONCEN | 117,17 | 57,111 | 20 |
| STEP\_UP\_MEDIA5REP\_uV\_GMED\_EXCEN | 60,59 | 22,274 | 20 |
| STEP\_UP\_MEDIA5REP\_uV\_GMAX\_CONCEN | 97,61 | 50,436 | 20 |
| STEP\_UP\_MEDIA5REP\_uV\_GMAX\_EXCEN | 42,99 | 25,891 | 20 |
| STEP\_UP\_MEDIA5REP\_uV\_BF\_CONCEN | 92,17 | 28,245 | 20 |
| STEP\_UP\_MEDIA5REP\_uV\_BF\_EXCEN | 51,85 | 14,092 | 20 |
| STEP\_UP\_MEDIA5REP\_uV\_VL\_CONCEN | 197,34 | 66,553 | 20 |
| STEP\_UP\_MEDIA5REP\_uV\_VL\_EXCEN | 111,73 | 32,703 | 20 |
| STEP\_UP\_MEDIA5REP\_uV\_VM\_CONCEN | 216,71 | 75,222 | 20 |
| STEP\_UP\_MEDIA5REP\_uV\_VM\_EXCEN | 124,13 | 34,070 | 20 |
| STEP\_UP\_MEDIA5REP\_uV\_RF\_CONCEN | 198,00 | 58,866 | 20 |
| STEP\_UP\_MEDIA5REP\_uV\_RF\_EXCEN | 118,27 | 35,252 | 20 |
| LUNGE\_MEDIA5REP\_uV\_GMED\_CONCEN | 106,25 | 34,477 | 20 |
| LUNGE\_MEDIA5REP\_uV\_GMED\_EXCEN | 71,59 | 19,223 | 20 |
| LUNGE\_MEDIA5REP\_uV\_GMAX\_CONCEN | 106,09 | 51,361 | 20 |
| LUNGE\_MEDIA5REP\_uV\_GMAX\_EXCEN | 55,02 | 19,466 | 20 |
| LUNGE\_MEDIA5REP\_uV\_BF\_CONCEN | 88,77 | 29,890 | 20 |
| LUNGE\_MEDIA5REP\_uV\_BF\_EXCEN | 62,92 | 23,950 | 20 |
| LUNGE\_MEDIA5REP\_uV\_VL\_CONCEN | 208,18 | 73,095 | 20 |
| LUNGE\_MEDIA5REP\_uV\_VL\_EXCEN | 164,73 | 48,894 | 20 |
| LUNGE\_MEDIA5REP\_uV\_VM\_CONCEN | 206,82 | 84,510 | 20 |
| LUNGE\_MEDIA5REP\_uV\_VM\_EXCEN | 175,50 | 62,777 | 20 |
| LUNGE\_MEDIA5REP\_uV\_RF\_CONCEN | 148,39 | 42,690 | 20 |
| LUNGE\_MEDIA5REP\_uV\_RF\_EXCEN | 128,23 | 34,449 | 20 |
| SQ\_MEDIA5REP\_uV\_GMED\_CONCEN | 194,68 | 90,573 | 20 |
| SQ\_MEDIA5REP\_uV\_GMED\_EXCEN | 141,07 | 63,288 | 20 |
| SQ\_MEDIA5REP\_uV\_GMAX\_CONCEN | 168,68 | 85,451 | 20 |
| SQ\_MEDIA5REP\_uV\_GMAX\_EXCEN | 102,39 | 39,251 | 20 |
| SQ\_MEDIA5REP\_uV\_BF\_CONCEN | 138,29 | 46,574 | 20 |
| SQ\_MEDIA5REP\_uV\_BF\_EXCEN | 101,54 | 29,637 | 20 |
| SQ\_MEDIA5REP\_uV\_VL\_CONCEN | 314,81 | 122,182 | 20 |
| SQ\_MEDIA5REP\_uV\_VL\_EXCEN | 261,87 | 99,179 | 20 |
| SQ\_MEDIA5REP\_uV\_VM\_CONCEN | 288,30 | 118,183 | 20 |
| SQ\_MEDIA5REP\_uV\_VM\_EXCEN | 257,90 | 106,115 | 20 |
| SQ\_MEDIA5REP\_uV\_RF\_CONCEN | 178,14 | 68,675 | 20 |
| SQ\_MEDIA5REP\_uV\_RF\_EXCEN | 161,50 | 57,646 | 20 |
|  |  |  |  |

Modelo lineal general

Pruebas multivarianteaPruebas multivariante, tabla, 1 niveles de cabeceras de columna y 2 niveles de cabeceras de fila, tabla con 10 columnas y 33 filas

|  |  |  |  |  |  |  |  |  |  |
| --- | --- | --- | --- | --- | --- | --- | --- | --- | --- |
| Efecto | | Valor | F | gl de hipótesis | gl de error | Sig. | Eta parcial al cuadrado | Parámetro sin centralidad | Potencia observadac |
| Ejerc | Traza de Pillai | ,820 | 41,099b | 2,000 | 18,000 | ,000 | ,820 | 82,198 | 1,000 |
|  | Lambda de Wilks | ,180 | 41,099b | 2,000 | 18,000 | ,000 | ,820 | 82,198 | 1,000 |
|  | Traza de Hotelling | 4,567 | 41,099b | 2,000 | 18,000 | ,000 | ,820 | 82,198 | 1,000 |
|  | Raíz mayor de Roy | 4,567 | 41,099b | 2,000 | 18,000 | ,000 | ,820 | 82,198 | 1,000 |
| Muscu | Traza de Pillai | ,916 | 32,733b | 5,000 | 15,000 | ,000 | ,916 | 163,666 | 1,000 |
|  | Lambda de Wilks | ,084 | 32,733b | 5,000 | 15,000 | ,000 | ,916 | 163,666 | 1,000 |
|  | Traza de Hotelling | 10,911 | 32,733b | 5,000 | 15,000 | ,000 | ,916 | 163,666 | 1,000 |
|  | Raíz mayor de Roy | 10,911 | 32,733b | 5,000 | 15,000 | ,000 | ,916 | 163,666 | 1,000 |
| act | Traza de Pillai | ,780 | 67,471b | 1,000 | 19,000 | ,000 | ,780 | 67,471 | 1,000 |
|  | Lambda de Wilks | ,220 | 67,471b | 1,000 | 19,000 | ,000 | ,780 | 67,471 | 1,000 |
|  | Traza de Hotelling | 3,551 | 67,471b | 1,000 | 19,000 | ,000 | ,780 | 67,471 | 1,000 |
|  | Raíz mayor de Roy | 3,551 | 67,471b | 1,000 | 19,000 | ,000 | ,780 | 67,471 | 1,000 |
| Ejerc \* Muscu | Traza de Pillai | ,867 | 6,529b | 10,000 | 10,000 | ,003 | ,867 | 65,294 | ,981 |
|  | Lambda de Wilks | ,133 | 6,529b | 10,000 | 10,000 | ,003 | ,867 | 65,294 | ,981 |
|  | Traza de Hotelling | 6,529 | 6,529b | 10,000 | 10,000 | ,003 | ,867 | 65,294 | ,981 |
|  | Raíz mayor de Roy | 6,529 | 6,529b | 10,000 | 10,000 | ,003 | ,867 | 65,294 | ,981 |
| Ejerc \* act | Traza de Pillai | ,685 | 19,535b | 2,000 | 18,000 | ,000 | ,685 | 39,070 | 1,000 |
|  | Lambda de Wilks | ,315 | 19,535b | 2,000 | 18,000 | ,000 | ,685 | 39,070 | 1,000 |
|  | Traza de Hotelling | 2,171 | 19,535b | 2,000 | 18,000 | ,000 | ,685 | 39,070 | 1,000 |
|  | Raíz mayor de Roy | 2,171 | 19,535b | 2,000 | 18,000 | ,000 | ,685 | 39,070 | 1,000 |
| Muscu \* act | Traza de Pillai | ,746 | 8,789b | 5,000 | 15,000 | ,000 | ,746 | 43,945 | ,996 |
|  | Lambda de Wilks | ,254 | 8,789b | 5,000 | 15,000 | ,000 | ,746 | 43,945 | ,996 |
|  | Traza de Hotelling | 2,930 | 8,789b | 5,000 | 15,000 | ,000 | ,746 | 43,945 | ,996 |
|  | Raíz mayor de Roy | 2,930 | 8,789b | 5,000 | 15,000 | ,000 | ,746 | 43,945 | ,996 |
| Ejerc \* Muscu \* act | Traza de Pillai | ,950 | 19,142b | 10,000 | 10,000 | ,000 | ,950 | 191,425 | 1,000 |
|  | Lambda de Wilks | ,050 | 19,142b | 10,000 | 10,000 | ,000 | ,950 | 191,425 | 1,000 |
|  | Traza de Hotelling | 19,142 | 19,142b | 10,000 | 10,000 | ,000 | ,950 | 191,425 | 1,000 |
|  | Raíz mayor de Roy | 19,142 | 19,142b | 10,000 | 10,000 | ,000 | ,950 | 191,425 | 1,000 |
|  |  |  |  |  |  |  |  |  |  |
| --- | --- | --- | --- | --- | --- | --- | --- | --- | --- |
| a. Diseño : Intersección   Diseño intra-sujetos: Ejerc + Muscu + act + Ejerc \* Muscu + Ejerc \* act + Muscu \* act + Ejerc \* Muscu \* act | | | | | | | | | |
| b. Estadístico exacto | | | | | | | | | |
| c. Se ha calculado utilizando alpha = ,05 | | | | | | | | | |
|  |  |  |  |  |  |  |  |  |  |

Modelo lineal general

Prueba de esfericidad de MauchlyaPrueba de esfericidad de Mauchly, tabla, Medida, MEASURE\_1, 1 capas, 2 niveles de cabeceras de columna y 1 niveles de cabeceras de fila, tabla con 8 columnas y 14 filas

| |  |  |  | | --- | --- | --- | | Medida: | MEASURE\_1 | MEASURE\_1 | | | | | | | | |
|  |  |  |  |  |  |  |  |
| --- | --- | --- | --- | --- | --- | --- | --- |
| Efecto intra-sujetos | W de Mauchly | Aprox. Chi-cuadrado | gl | Sig. | Épsilonb | | |
|  |  |  |  |  | Greenhouse-Geisser | Huynh-Feldt | Límite inferior |
| Ejerc | ,775 | 4,587 | 2 | ,101 | ,816 | ,883 | ,500 |
| Muscu | ,204 | 27,212 | 14 | ,019 | ,607 | ,735 | ,200 |
| act | 1,000 | ,000 | 0 | . | 1,000 | 1,000 | 1,000 |
| Ejerc \* Muscu | ,001 | 110,746 | 54 | ,000 | ,485 | ,671 | ,100 |
| Ejerc \* act | ,835 | 3,256 | 2 | ,196 | ,858 | ,935 | ,500 |
| Muscu \* act | ,140 | 33,662 | 14 | ,003 | ,552 | ,654 | ,200 |
| Ejerc \* Muscu \* act | ,000 | 127,177 | 54 | ,000 | ,418 | ,550 | ,100 |
|  |  |  |  |  |  |  |  |
| --- | --- | --- | --- | --- | --- | --- | --- |
| Prueba la hipótesis nula de que la matriz de covarianzas de error de las variables dependientes con transformación ortonormalizada es proporcional a una matriz de identidad. | | | | | | | |
| a. Diseño : Intersección   Diseño intra-sujetos: Ejerc + Muscu + act + Ejerc \* Muscu + Ejerc \* act + Muscu \* act + Ejerc \* Muscu \* act | | | | | | | |
| b. Se puede utilizar para ajustar los grados de libertad para las pruebas promedio de significación. Las pruebas corregidas se visualizan en la tabla de pruebas de efectos intra-sujetos. | | | | | | | |
|  |  |  |  |  |  |  |  |

Modelo lineal general

Pruebas de efectos intra-sujetosPruebas de efectos intra-sujetos, tabla, Medida, MEASURE\_1, 1 capas, 1 niveles de cabeceras de columna y 2 niveles de cabeceras de fila, tabla con 10 columnas y 60 filas

| |  |  |  | | --- | --- | --- | | Medida: | MEASURE\_1 | MEASURE\_1 | | | | | | | | | | |
|  |  |  |  |  |  |  |  |  |  |
| --- | --- | --- | --- | --- | --- | --- | --- | --- | --- |
| Origen | | Tipo III de suma de cuadrados | gl | Media cuadrática | F | Sig. | Eta parcial al cuadrado | Parámetro sin centralidad | Potencia observadaa |
| Ejerc | Esfericidad asumida | 779534,577 | 2 | 389767,289 | 63,956 | ,000 | ,771 | 127,913 | 1,000 |
|  | Greenhouse-Geisser | 779534,577 | 1,633 | 477446,926 | 63,956 | ,000 | ,771 | 104,423 | 1,000 |
|  | Huynh-Feldt | 779534,577 | 1,765 | 441647,756 | 63,956 | ,000 | ,771 | 112,887 | 1,000 |
|  | Límite inferior | 779534,577 | 1,000 | 779534,577 | 63,956 | ,000 | ,771 | 63,956 | 1,000 |
| Error(Ejerc) | Esfericidad asumida | 231581,920 | 38 | 6094,261 |  |  |  |  |  |
|  | Greenhouse-Geisser | 231581,920 | 31,022 | 7465,188 |  |  |  |  |  |
|  | Huynh-Feldt | 231581,920 | 33,536 | 6905,445 |  |  |  |  |  |
|  | Límite inferior | 231581,920 | 19,000 | 12188,522 |  |  |  |  |  |
| Muscu | Esfericidad asumida | 1821014,802 | 5 | 364202,960 | 35,417 | ,000 | ,651 | 177,087 | 1,000 |
|  | Greenhouse-Geisser | 1821014,802 | 3,034 | 600234,897 | 35,417 | ,000 | ,651 | 107,451 | 1,000 |
|  | Huynh-Feldt | 1821014,802 | 3,675 | 495505,031 | 35,417 | ,000 | ,651 | 130,162 | 1,000 |
|  | Límite inferior | 1821014,802 | 1,000 | 1821014,802 | 35,417 | ,000 | ,651 | 35,417 | 1,000 |
| Error(Muscu) | Esfericidad asumida | 976898,029 | 95 | 10283,137 |  |  |  |  |  |
|  | Greenhouse-Geisser | 976898,029 | 57,643 | 16947,412 |  |  |  |  |  |
|  | Huynh-Feldt | 976898,029 | 69,826 | 13990,403 |  |  |  |  |  |
|  | Límite inferior | 976898,029 | 19,000 | 51415,686 |  |  |  |  |  |
| act | Esfericidad asumida | 422997,698 | 1 | 422997,698 | 67,471 | ,000 | ,780 | 67,471 | 1,000 |
|  | Greenhouse-Geisser | 422997,698 | 1,000 | 422997,698 | 67,471 | ,000 | ,780 | 67,471 | 1,000 |
|  | Huynh-Feldt | 422997,698 | 1,000 | 422997,698 | 67,471 | ,000 | ,780 | 67,471 | 1,000 |
|  | Límite inferior | 422997,698 | 1,000 | 422997,698 | 67,471 | ,000 | ,780 | 67,471 | 1,000 |
| Error(act) | Esfericidad asumida | 119116,986 | 19 | 6269,315 |  |  |  |  |  |
|  | Greenhouse-Geisser | 119116,986 | 19,000 | 6269,315 |  |  |  |  |  |
|  | Huynh-Feldt | 119116,986 | 19,000 | 6269,315 |  |  |  |  |  |
|  | Límite inferior | 119116,986 | 19,000 | 6269,315 |  |  |  |  |  |
| Ejerc \* Muscu | Esfericidad asumida | 188766,711 | 10 | 18876,671 | 11,616 | ,000 | ,379 | 116,158 | 1,000 |
|  | Greenhouse-Geisser | 188766,711 | 4,848 | 38934,689 | 11,616 | ,000 | ,379 | 56,317 | 1,000 |
|  | Huynh-Feldt | 188766,711 | 6,711 | 28129,819 | 11,616 | ,000 | ,379 | 77,948 | 1,000 |
|  | Límite inferior | 188766,711 | 1,000 | 188766,711 | 11,616 | ,003 | ,379 | 11,616 | ,898 |
| Error(Ejerc\*Muscu) | Esfericidad asumida | 308767,218 | 190 | 1625,091 |  |  |  |  |  |
|  | Greenhouse-Geisser | 308767,218 | 92,118 | 3351,883 |  |  |  |  |  |
|  | Huynh-Feldt | 308767,218 | 127,501 | 2421,693 |  |  |  |  |  |
|  | Límite inferior | 308767,218 | 19,000 | 16250,906 |  |  |  |  |  |
| Ejerc \* act | Esfericidad asumida | 37246,386 | 2 | 18623,193 | 25,673 | ,000 | ,575 | 51,347 | 1,000 |
|  | Greenhouse-Geisser | 37246,386 | 1,716 | 21705,231 | 25,673 | ,000 | ,575 | 44,056 | 1,000 |
|  | Huynh-Feldt | 37246,386 | 1,870 | 19918,389 | 25,673 | ,000 | ,575 | 48,008 | 1,000 |
|  | Límite inferior | 37246,386 | 1,000 | 37246,386 | 25,673 | ,000 | ,575 | 25,673 | ,998 |
| Error(Ejerc\*act) | Esfericidad asumida | 27564,676 | 38 | 725,386 |  |  |  |  |  |
|  | Greenhouse-Geisser | 27564,676 | 32,604 | 845,434 |  |  |  |  |  |
|  | Huynh-Feldt | 27564,676 | 35,529 | 775,835 |  |  |  |  |  |
|  | Límite inferior | 27564,676 | 19,000 | 1450,772 |  |  |  |  |  |
| Muscu \* act | Esfericidad asumida | 15878,636 | 5 | 3175,727 | 4,304 | ,001 | ,185 | 21,521 | ,955 |
|  | Greenhouse-Geisser | 15878,636 | 2,758 | 5758,138 | 4,304 | ,010 | ,185 | 11,869 | ,817 |
|  | Huynh-Feldt | 15878,636 | 3,272 | 4852,259 | 4,304 | ,007 | ,185 | 14,085 | ,866 |
|  | Límite inferior | 15878,636 | 1,000 | 15878,636 | 4,304 | ,052 | ,185 | 4,304 | ,504 |
| Error(Muscu\*act) | Esfericidad asumida | 70094,426 | 95 | 737,836 |  |  |  |  |  |
|  | Greenhouse-Geisser | 70094,426 | 52,394 | 1337,823 |  |  |  |  |  |
|  | Huynh-Feldt | 70094,426 | 62,176 | 1127,355 |  |  |  |  |  |
|  | Límite inferior | 70094,426 | 19,000 | 3689,180 |  |  |  |  |  |
| Ejerc \* Muscu \* act | Esfericidad asumida | 28306,780 | 10 | 2830,678 | 7,777 | ,000 | ,290 | 77,766 | 1,000 |
|  | Greenhouse-Geisser | 28306,780 | 4,178 | 6774,511 | 7,777 | ,000 | ,290 | 32,494 | ,997 |
|  | Huynh-Feldt | 28306,780 | 5,503 | 5143,544 | 7,777 | ,000 | ,290 | 42,798 | 1,000 |
|  | Límite inferior | 28306,780 | 1,000 | 28306,780 | 7,777 | ,012 | ,290 | 7,777 | ,754 |
| Error(Ejerc\*Muscu\*act) | Esfericidad asumida | 69159,731 | 190 | 363,999 |  |  |  |  |  |
|  | Greenhouse-Geisser | 69159,731 | 79,390 | 871,138 |  |  |  |  |  |
|  | Huynh-Feldt | 69159,731 | 104,564 | 661,411 |  |  |  |  |  |
|  | Límite inferior | 69159,731 | 19,000 | 3639,986 |  |  |  |  |  |
|  |  |  |  |  |  |  |  |  |  |
| --- | --- | --- | --- | --- | --- | --- | --- | --- | --- |
| a. Se ha calculado utilizando alpha = ,05 | | | | | | | | | |
|  |  |  |  |  |  |  |  |  |  |

Modelo lineal general

Pruebas de contrastes intra-sujetosPruebas de contrastes intra-sujetos, tabla, Medida, MEASURE\_1, 1 capas, 1 niveles de cabeceras de columna y 4 niveles de cabeceras de fila, tabla con 12 columnas y 74 filas

| |  |  |  | | --- | --- | --- | | Medida: | MEASURE\_1 | MEASURE\_1 | | | | | | | | | | | | |  |  |
|  |  |  |  |  |  |  |  |  |  |  |  |  |  |
| --- | --- | --- | --- | --- | --- | --- | --- | --- | --- | --- | --- | --- | --- |
| Origen | Ejerc | Muscu | act | Tipo III de suma de cuadrados | gl | Media cuadrática | F | Sig. | Eta parcial al cuadrado | Parámetro sin centralidad | Potencia observadaa |  |  |
| Ejerc | Lineal |  |  | 646228,310 | 1 | 646228,310 | 80,976 | ,000 | ,810 | 80,976 | 1,000 |  |  |
|  | Cuadrático |  |  | 133306,267 | 1 | 133306,267 | 31,679 | ,000 | ,625 | 31,679 | 1,000 |  |  |
| Error(Ejerc) | Lineal |  |  | 151629,785 | 19 | 7980,515 |  |  |  |  |  |  |  |
|  | Cuadrático |  |  | 79952,135 | 19 | 4208,007 |  |  |  |  |  |  |  |
| Muscu |  | Lineal |  | 769068,188 | 1 | 769068,188 | 105,138 | ,000 | ,847 | 105,138 | 1,000 |  |  |
|  |  | Cuadrático |  | 32110,008 | 1 | 32110,008 | 3,841 | ,065 | ,168 | 3,841 | ,460 |  |  |
|  |  | Cúbico |  | 797560,070 | 1 | 797560,070 | 74,665 | ,000 | ,797 | 74,665 | 1,000 |  |  |
|  |  | Orden 4 |  | 11748,088 | 1 | 11748,088 | 1,509 | ,234 | ,074 | 1,509 | ,215 |  |  |
|  |  | Orden 5 |  | 210528,448 | 1 | 210528,448 | 12,188 | ,002 | ,391 | 12,188 | ,911 |  |  |
| Error(Muscu) |  | Lineal |  | 138981,944 | 19 | 7314,839 |  |  |  |  |  |  |  |
|  |  | Cuadrático |  | 158841,630 | 19 | 8360,086 |  |  |  |  |  |  |  |
|  |  | Cúbico |  | 202955,828 | 19 | 10681,886 |  |  |  |  |  |  |  |
|  |  | Orden 4 |  | 147913,145 | 19 | 7784,902 |  |  |  |  |  |  |  |
|  |  | Orden 5 |  | 328205,483 | 19 | 17273,973 |  |  |  |  |  |  |  |
| act |  |  | Lineal | 422997,698 | 1 | 422997,698 | 67,471 | ,000 | ,780 | 67,471 | 1,000 |  |  |
| Error(act) |  |  | Lineal | 119116,986 | 19 | 6269,315 |  |  |  |  |  |  |  |
| Ejerc \* Muscu | Lineal | Lineal |  | 5466,011 | 1 | 5466,011 | 2,776 | ,112 | ,127 | 2,776 | ,353 |  |  |
|  |  | Cuadrático |  | 46379,580 | 1 | 46379,580 | 16,243 | ,001 | ,461 | 16,243 | ,969 |  |  |
|  |  | Cúbico |  | 98651,481 | 1 | 98651,481 | 41,037 | ,000 | ,684 | 41,037 | 1,000 |  |  |
|  |  | Orden 4 |  | 1760,447 | 1 | 1760,447 | ,711 | ,410 | ,036 | ,711 | ,126 |  |  |
|  |  | Orden 5 |  | 28998,249 | 1 | 28998,249 | 11,162 | ,003 | ,370 | 11,162 | ,886 |  |  |
|  | Cuadrático | Lineal |  | 328,664 | 1 | 328,664 | ,246 | ,625 | ,013 | ,246 | ,076 |  |  |
|  |  | Cuadrático |  | 867,101 | 1 | 867,101 | ,833 | ,373 | ,042 | ,833 | ,140 |  |  |
|  |  | Cúbico |  | 5129,330 | 1 | 5129,330 | 7,081 | ,015 | ,272 | 7,081 | ,714 |  |  |
|  |  | Orden 4 |  | 257,325 | 1 | 257,325 | ,501 | ,488 | ,026 | ,501 | ,103 |  |  |
|  |  | Orden 5 |  | 928,522 | 1 | 928,522 | 2,761 | ,113 | ,127 | 2,761 | ,351 |  |  |
| Error(Ejerc\*Muscu) | Lineal | Lineal |  | 37413,414 | 19 | 1969,127 |  |  |  |  |  |  |  |
|  |  | Cuadrático |  | 54250,485 | 19 | 2855,289 |  |  |  |  |  |  |  |
|  |  | Cúbico |  | 45674,803 | 19 | 2403,937 |  |  |  |  |  |  |  |
|  |  | Orden 4 |  | 47040,388 | 19 | 2475,810 |  |  |  |  |  |  |  |
|  |  | Orden 5 |  | 49359,597 | 19 | 2597,874 |  |  |  |  |  |  |  |
|  | Cuadrático | Lineal |  | 25348,350 | 19 | 1334,124 |  |  |  |  |  |  |  |
|  |  | Cuadrático |  | 19769,189 | 19 | 1040,484 |  |  |  |  |  |  |  |
|  |  | Cúbico |  | 13762,683 | 19 | 724,352 |  |  |  |  |  |  |  |
|  |  | Orden 4 |  | 9759,454 | 19 | 513,655 |  |  |  |  |  |  |  |
|  |  | Orden 5 |  | 6388,856 | 19 | 336,256 |  |  |  |  |  |  |  |
| Ejerc \* act | Lineal |  | Lineal | 19459,080 | 1 | 19459,080 | 21,413 | ,000 | ,530 | 21,413 | ,992 |  |  |
|  | Cuadrático |  | Lineal | 17787,306 | 1 | 17787,306 | 32,817 | ,000 | ,633 | 32,817 | 1,000 |  |  |
| Error(Ejerc\*act) | Lineal |  | Lineal | 17266,502 | 19 | 908,763 |  |  |  |  |  |  |  |
|  | Cuadrático |  | Lineal | 10298,174 | 19 | 542,009 |  |  |  |  |  |  |  |
| Muscu \* act |  | Lineal | Lineal | 635,910 | 1 | 635,910 | ,568 | ,460 | ,029 | ,568 | ,111 |  |  |
|  |  | Cuadrático | Lineal | 1003,972 | 1 | 1003,972 | 2,247 | ,150 | ,106 | 2,247 | ,296 |  |  |
|  |  | Cúbico | Lineal | 2067,832 | 1 | 2067,832 | 2,459 | ,133 | ,115 | 2,459 | ,319 |  |  |
|  |  | Orden 4 | Lineal | 2594,246 | 1 | 2594,246 | 4,124 | ,057 | ,178 | 4,124 | ,487 |  |  |
|  |  | Orden 5 | Lineal | 9576,677 | 1 | 9576,677 | 14,662 | ,001 | ,436 | 14,662 | ,952 |  |  |
| Error(Muscu\*act) |  | Lineal | Lineal | 21262,311 | 19 | 1119,069 |  |  |  |  |  |  |  |
|  |  | Cuadrático | Lineal | 8488,569 | 19 | 446,767 |  |  |  |  |  |  |  |
|  |  | Cúbico | Lineal | 15980,795 | 19 | 841,094 |  |  |  |  |  |  |  |
|  |  | Orden 4 | Lineal | 11952,822 | 19 | 629,096 |  |  |  |  |  |  |  |
|  |  | Orden 5 | Lineal | 12409,928 | 19 | 653,154 |  |  |  |  |  |  |  |
| Ejerc \* Muscu \* act | Lineal | Lineal | Lineal | 21705,469 | 1 | 21705,469 | 34,528 | ,000 | ,645 | 34,528 | 1,000 |  |  |
|  |  | Cuadrático | Lineal | 1082,091 | 1 | 1082,091 | 3,134 | ,093 | ,142 | 3,134 | ,390 |  |  |
|  |  | Cúbico | Lineal | 3075,627 | 1 | 3075,627 | 4,066 | ,058 | ,176 | 4,066 | ,482 |  |  |
|  |  | Orden 4 | Lineal | 30,132 | 1 | 30,132 | ,082 | ,778 | ,004 | ,082 | ,059 |  |  |
|  |  | Orden 5 | Lineal | 6,522 | 1 | 6,522 | ,025 | ,875 | ,001 | ,025 | ,053 |  |  |
|  | Cuadrático | Lineal | Lineal | 1225,692 | 1 | 1225,692 | 2,828 | ,109 | ,130 | 2,828 | ,358 |  |  |
|  |  | Cuadrático | Lineal | 188,268 | 1 | 188,268 | ,718 | ,407 | ,036 | ,718 | ,127 |  |  |
|  |  | Cúbico | Lineal | 949,393 | 1 | 949,393 | 4,516 | ,047 | ,192 | 4,516 | ,523 |  |  |
|  |  | Orden 4 | Lineal | 11,058 | 1 | 11,058 | ,039 | ,846 | ,002 | ,039 | ,054 |  |  |
|  |  | Orden 5 | Lineal | 32,528 | 1 | 32,528 | ,345 | ,564 | ,018 | ,345 | ,086 |  |  |
| Error(Ejerc\*Muscu\*act) | Lineal | Lineal | Lineal | 11943,916 | 19 | 628,627 |  |  |  |  |  |  |  |
|  |  | Cuadrático | Lineal | 6560,843 | 19 | 345,308 |  |  |  |  |  |  |  |
|  |  | Cúbico | Lineal | 14370,375 | 19 | 756,336 |  |  |  |  |  |  |  |
|  |  | Orden 4 | Lineal | 6995,747 | 19 | 368,197 |  |  |  |  |  |  |  |
|  |  | Orden 5 | Lineal | 4875,047 | 19 | 256,581 |  |  |  |  |  |  |  |
|  | Cuadrático | Lineal | Lineal | 8235,978 | 19 | 433,473 |  |  |  |  |  |  |  |
|  |  | Cuadrático | Lineal | 4979,295 | 19 | 262,068 |  |  |  |  |  |  |  |
|  |  | Cúbico | Lineal | 3994,571 | 19 | 210,241 |  |  |  |  |  |  |  |
|  |  | Orden 4 | Lineal | 5414,690 | 19 | 284,984 |  |  |  |  |  |  |  |
|  |  | Orden 5 | Lineal | 1789,270 | 19 | 94,172 |  |  |  |  |  |  |  |
|  |  |  |  |  |  |  |  |  |  |  |  |  |  |
| --- | --- | --- | --- | --- | --- | --- | --- | --- | --- | --- | --- | --- | --- |
| a. Se ha calculado utilizando alpha = ,05 | | | | | | | | | | | |  |  |
|  |  |  |  |  |  |  |  |  |  |  |  |  |  |

Modelo lineal general

Pruebas de efectos inter-sujetosPruebas de efectos inter-sujetos, tabla, Medida, MEASURE\_1, Variable transformada, Promedio, 1 capas, 1 niveles de cabeceras de columna y 1 niveles de cabeceras de fila, tabla con 9 columnas y 7 filas

| |  |  |  | | --- | --- | --- | | Medida: | MEASURE\_1 | MEASURE\_1 | | Variable transformada: | Promedio | Promedio | | | | | | | | | |
|  |  |  |  |  |  |  |  |  |
| --- | --- | --- | --- | --- | --- | --- | --- | --- |
| Origen | Tipo III de suma de cuadrados | gl | Media cuadrática | F | Sig. | Eta parcial al cuadrado | Parámetro sin centralidad | Potencia observadaa |
| Intersección | 15372174,694 | 1 | 15372174,694 | 383,456 | ,000 | ,953 | 383,456 | 1,000 |
| Error | 761681,769 | 19 | 40088,514 |  |  |  |  |  |
|  |  |  |  |  |  |  |  |  |
| --- | --- | --- | --- | --- | --- | --- | --- | --- |
| a. Se ha calculado utilizando alpha = ,05 | | | | | | | | |
|  |  |  |  |  |  |  |  |  |

1. Ejerc

EstimacionesEstimaciones, tabla, Medida, MEASURE\_1, 1 capas, 2 niveles de cabeceras de columna y 1 niveles de cabeceras de fila, tabla con 5 columnas y 7 filas

| |  |  |  | | --- | --- | --- | | Medida: | MEASURE\_1 | MEASURE\_1 | | | | | |
|  |  |  |  |  |
| --- | --- | --- | --- | --- |
| Ejerc | Media | Desv. Error | Intervalo de confianza al 95% | |
|  |  |  | Límite inferior | Límite superior |
| 1 | 119,047 | 6,225 | 106,017 | 132,077 |
| 2 | 126,874 | 6,201 | 113,896 | 139,852 |
| 3 | 192,431 | 11,858 | 167,611 | 217,250 |
|  |  |  |  |  |

1. Ejerc

Comparaciones por parejasComparaciones por parejas, tabla, Medida, MEASURE\_1, 1 capas, 2 niveles de cabeceras de columna y 2 niveles de cabeceras de fila, tabla con 7 columnas y 13 filas

| |  |  |  | | --- | --- | --- | | Medida: | MEASURE\_1 | MEASURE\_1 | | | | | | | |
|  |  |  |  |  |  |  |
| --- | --- | --- | --- | --- | --- | --- |
| (I) Ejerc | (J) Ejerc | Diferencia de medias (I-J) | Desv. Error | Sig.b | 95% de intervalo de confianza para diferenciab | |
|  |  |  |  |  | Límite inferior | Límite superior |
| 1 | 2 | -7,828 | 5,208 | ,448 | -21,498 | 5,843 |
|  | 3 | -73,384\* | 8,155 | ,000 | -94,792 | -51,976 |
| 2 | 1 | 7,828 | 5,208 | ,448 | -5,843 | 21,498 |
|  | 3 | -65,557\* | 7,664 | ,000 | -85,675 | -45,439 |
| 3 | 1 | 73,384\* | 8,155 | ,000 | 51,976 | 94,792 |
|  | 2 | 65,557\* | 7,664 | ,000 | 45,439 | 85,675 |
|  |  |  |  |  |  |  |
| --- | --- | --- | --- | --- | --- | --- |
| Se basa en medias marginales estimadas | | | | | | |
| \*. La diferencia de medias es significativa en el nivel ,05. | | | | | | |
| b. Ajuste para varias comparaciones: Bonferroni. | | | | | | |
|  |  |  |  |  |  |  |

1. Ejerc

Pruebas multivariantePruebas multivariante, tabla, 1 niveles de cabeceras de columna y 1 niveles de cabeceras de fila, tabla con 9 columnas y 9 filas

|  |  |  |  |  |  |  |  |  |
| --- | --- | --- | --- | --- | --- | --- | --- | --- |
|  | Valor | F | gl de hipótesis | gl de error | Sig. | Eta parcial al cuadrado | Parámetro sin centralidad | Potencia observadab |
| Traza de Pillai | ,820 | 41,099a | 2,000 | 18,000 | ,000 | ,820 | 82,198 | 1,000 |
| Lambda de Wilks | ,180 | 41,099a | 2,000 | 18,000 | ,000 | ,820 | 82,198 | 1,000 |
| Traza de Hotelling | 4,567 | 41,099a | 2,000 | 18,000 | ,000 | ,820 | 82,198 | 1,000 |
| Raíz mayor de Roy | 4,567 | 41,099a | 2,000 | 18,000 | ,000 | ,820 | 82,198 | 1,000 |
|  |  |  |  |  |  |  |  |  |
| --- | --- | --- | --- | --- | --- | --- | --- | --- |
| Cada F prueba el efecto multivariante de Ejerc. Estas pruebas se basan en las comparaciones por parejas linealmente independientes entre las medias marginales estimadas. | | | | | | | | |
| a. Estadístico exacto | | | | | | | | |
| b. Se ha calculado utilizando alpha = ,05 | | | | | | | | |
|  |  |  |  |  |  |  |  |  |

2. Muscu

EstimacionesEstimaciones, tabla, Medida, MEASURE\_1, 1 capas, 2 niveles de cabeceras de columna y 1 niveles de cabeceras de fila, tabla con 5 columnas y 10 filas

| |  |  |  | | --- | --- | --- | | Medida: | MEASURE\_1 | MEASURE\_1 | | | | | |
|  |  |  |  |  |
| --- | --- | --- | --- | --- |
| Muscu | Media | Desv. Error | Intervalo de confianza al 95% | |
|  |  |  | Límite inferior | Límite superior |
| 1 | 115,225 | 9,328 | 95,701 | 134,749 |
| 2 | 95,463 | 8,633 | 77,395 | 113,531 |
| 3 | 89,257 | 4,979 | 78,836 | 99,677 |
| 4 | 209,777 | 14,934 | 178,519 | 241,035 |
| 5 | 211,560 | 16,279 | 177,487 | 245,633 |
| 6 | 155,422 | 9,389 | 135,770 | 175,074 |
|  |  |  |  |  |

2. Muscu

Comparaciones por parejasComparaciones por parejas, tabla, Medida, MEASURE\_1, 1 capas, 2 niveles de cabeceras de columna y 2 niveles de cabeceras de fila, tabla con 7 columnas y 37 filas

| |  |  |  | | --- | --- | --- | | Medida: | MEASURE\_1 | MEASURE\_1 | | | | | | | |
|  |  |  |  |  |  |  |
| --- | --- | --- | --- | --- | --- | --- |
| (I) Muscu | (J) Muscu | Diferencia de medias (I-J) | Desv. Error | Sig.b | 95% de intervalo de confianza para diferenciab | |
|  |  |  |  |  | Límite inferior | Límite superior |
| 1 | 2 | 19,762 | 8,868 | ,572 | -9,981 | 49,504 |
|  | 3 | 25,968 | 8,440 | ,093 | -2,341 | 54,278 |
|  | 4 | -94,552\* | 13,823 | ,000 | -140,916 | -48,187 |
|  | 5 | -96,335\* | 12,931 | ,000 | -139,705 | -52,965 |
|  | 6 | -40,197\* | 9,273 | ,005 | -71,299 | -9,095 |
| 2 | 1 | -19,762 | 8,868 | ,572 | -49,504 | 9,981 |
|  | 3 | 6,207 | 9,677 | 1,000 | -26,249 | 38,662 |
|  | 4 | -114,313\* | 11,679 | ,000 | -153,485 | -75,141 |
|  | 5 | -116,097\* | 16,094 | ,000 | -170,077 | -62,117 |
|  | 6 | -59,958\* | 11,671 | ,001 | -99,104 | -20,813 |
| 3 | 1 | -25,968 | 8,440 | ,093 | -54,278 | 2,341 |
|  | 2 | -6,207 | 9,677 | 1,000 | -38,662 | 26,249 |
|  | 4 | -120,520\* | 15,583 | ,000 | -172,787 | -68,253 |
|  | 5 | -122,303\* | 13,802 | ,000 | -168,597 | -76,010 |
|  | 6 | -66,165\* | 8,390 | ,000 | -94,306 | -38,024 |
| 4 | 1 | 94,552\* | 13,823 | ,000 | 48,187 | 140,916 |
|  | 2 | 114,313\* | 11,679 | ,000 | 75,141 | 153,485 |
|  | 3 | 120,520\* | 15,583 | ,000 | 68,253 | 172,787 |
|  | 5 | -1,783 | 19,078 | 1,000 | -65,771 | 62,204 |
|  | 6 | 54,355 | 16,934 | ,069 | -2,441 | 111,151 |
| 5 | 1 | 96,335\* | 12,931 | ,000 | 52,965 | 139,705 |
|  | 2 | 116,097\* | 16,094 | ,000 | 62,117 | 170,077 |
|  | 3 | 122,303\* | 13,802 | ,000 | 76,010 | 168,597 |
|  | 4 | 1,783 | 19,078 | 1,000 | -62,204 | 65,771 |
|  | 6 | 56,138\* | 14,033 | ,011 | 9,072 | 103,205 |
| 6 | 1 | 40,197\* | 9,273 | ,005 | 9,095 | 71,299 |
|  | 2 | 59,958\* | 11,671 | ,001 | 20,813 | 99,104 |
|  | 3 | 66,165\* | 8,390 | ,000 | 38,024 | 94,306 |
|  | 4 | -54,355 | 16,934 | ,069 | -111,151 | 2,441 |
|  | 5 | -56,138\* | 14,033 | ,011 | -103,205 | -9,072 |
|  |  |  |  |  |  |  |
| --- | --- | --- | --- | --- | --- | --- |
| Se basa en medias marginales estimadas | | | | | | |
| \*. La diferencia de medias es significativa en el nivel ,05. | | | | | | |
| b. Ajuste para varias comparaciones: Bonferroni. | | | | | | |
|  |  |  |  |  |  |  |

2. Muscu

Pruebas multivariantePruebas multivariante, tabla, 1 niveles de cabeceras de columna y 1 niveles de cabeceras de fila, tabla con 9 columnas y 9 filas

|  |  |  |  |  |  |  |  |  |
| --- | --- | --- | --- | --- | --- | --- | --- | --- |
|  | Valor | F | gl de hipótesis | gl de error | Sig. | Eta parcial al cuadrado | Parámetro sin centralidad | Potencia observadab |
| Traza de Pillai | ,916 | 32,733a | 5,000 | 15,000 | ,000 | ,916 | 163,666 | 1,000 |
| Lambda de Wilks | ,084 | 32,733a | 5,000 | 15,000 | ,000 | ,916 | 163,666 | 1,000 |
| Traza de Hotelling | 10,911 | 32,733a | 5,000 | 15,000 | ,000 | ,916 | 163,666 | 1,000 |
| Raíz mayor de Roy | 10,911 | 32,733a | 5,000 | 15,000 | ,000 | ,916 | 163,666 | 1,000 |
|  |  |  |  |  |  |  |  |  |
| --- | --- | --- | --- | --- | --- | --- | --- | --- |
| Cada F prueba el efecto multivariante de Muscu. Estas pruebas se basan en las comparaciones por parejas linealmente independientes entre las medias marginales estimadas. | | | | | | | | |
| a. Estadístico exacto | | | | | | | | |
| b. Se ha calculado utilizando alpha = ,05 | | | | | | | | |
|  |  |  |  |  |  |  |  |  |

3. act

EstimacionesEstimaciones, tabla, Medida, MEASURE\_1, 1 capas, 2 niveles de cabeceras de columna y 1 niveles de cabeceras de fila, tabla con 5 columnas y 6 filas

| |  |  |  | | --- | --- | --- | | Medida: | MEASURE\_1 | MEASURE\_1 | | | | | |
|  |  |  |  |  |
| --- | --- | --- | --- | --- |
| act | Media | Desv. Error | Intervalo de confianza al 95% | |
|  |  |  | Límite inferior | Límite superior |
| 1 | 170,356 | 10,148 | 149,115 | 191,596 |
| 2 | 121,879 | 5,078 | 111,250 | 132,507 |
|  |  |  |  |  |

3. act

Comparaciones por parejasComparaciones por parejas, tabla, Medida, MEASURE\_1, 1 capas, 2 niveles de cabeceras de columna y 2 niveles de cabeceras de fila, tabla con 7 columnas y 9 filas

| |  |  |  | | --- | --- | --- | | Medida: | MEASURE\_1 | MEASURE\_1 | | | | | | | |
|  |  |  |  |  |  |  |
| --- | --- | --- | --- | --- | --- | --- |
| (I) act | (J) act | Diferencia de medias (I-J) | Desv. Error | Sig.b | 95% de intervalo de confianza para diferenciab | |
|  |  |  |  |  | Límite inferior | Límite superior |
| 1 | 2 | 48,477\* | 5,902 | ,000 | 36,124 | 60,829 |
| 2 | 1 | -48,477\* | 5,902 | ,000 | -60,829 | -36,124 |
|  |  |  |  |  |  |  |
| --- | --- | --- | --- | --- | --- | --- |
| Se basa en medias marginales estimadas | | | | | | |
| \*. La diferencia de medias es significativa en el nivel ,05. | | | | | | |
| b. Ajuste para varias comparaciones: Bonferroni. | | | | | | |
|  |  |  |  |  |  |  |

3. act

Pruebas multivariantePruebas multivariante, tabla, 1 niveles de cabeceras de columna y 1 niveles de cabeceras de fila, tabla con 9 columnas y 9 filas

|  |  |  |  |  |  |  |  |  |
| --- | --- | --- | --- | --- | --- | --- | --- | --- |
|  | Valor | F | gl de hipótesis | gl de error | Sig. | Eta parcial al cuadrado | Parámetro sin centralidad | Potencia observadab |
| Traza de Pillai | ,780 | 67,471a | 1,000 | 19,000 | ,000 | ,780 | 67,471 | 1,000 |
| Lambda de Wilks | ,220 | 67,471a | 1,000 | 19,000 | ,000 | ,780 | 67,471 | 1,000 |
| Traza de Hotelling | 3,551 | 67,471a | 1,000 | 19,000 | ,000 | ,780 | 67,471 | 1,000 |
| Raíz mayor de Roy | 3,551 | 67,471a | 1,000 | 19,000 | ,000 | ,780 | 67,471 | 1,000 |
|  |  |  |  |  |  |  |  |  |
| --- | --- | --- | --- | --- | --- | --- | --- | --- |
| Cada F prueba el efecto multivariante de act. Estas pruebas se basan en las comparaciones por parejas linealmente independientes entre las medias marginales estimadas. | | | | | | | | |
| a. Estadístico exacto | | | | | | | | |
| b. Se ha calculado utilizando alpha = ,05 | | | | | | | | |
|  |  |  |  |  |  |  |  |  |

4. Ejerc \* Muscu

EstimacionesEstimaciones, tabla, Medida, MEASURE\_1, 1 capas, 2 niveles de cabeceras de columna y 2 niveles de cabeceras de fila, tabla con 6 columnas y 22 filas

| |  |  |  | | --- | --- | --- | | Medida: | MEASURE\_1 | MEASURE\_1 | | | | | | |
|  |  |  |  |  |  |
| --- | --- | --- | --- | --- | --- |
| Ejerc | Muscu | Media | Desv. Error | Intervalo de confianza al 95% | |
|  |  |  |  | Límite inferior | Límite superior |
| 1 | 1 | 88,880 | 8,179 | 71,762 | 105,998 |
|  | 2 | 70,300 | 7,109 | 55,421 | 85,179 |
|  | 3 | 72,010 | 4,259 | 63,096 | 80,924 |
|  | 4 | 154,535 | 10,803 | 131,924 | 177,146 |
|  | 5 | 170,420 | 11,629 | 146,081 | 194,759 |
|  | 6 | 158,135 | 9,925 | 137,363 | 178,907 |
| 2 | 1 | 88,920 | 5,645 | 77,106 | 100,734 |
|  | 2 | 80,555 | 7,413 | 65,039 | 96,071 |
|  | 3 | 75,845 | 5,580 | 64,166 | 87,524 |
|  | 4 | 186,455 | 13,287 | 158,646 | 214,264 |
|  | 5 | 191,160 | 15,664 | 158,374 | 223,946 |
|  | 6 | 138,310 | 7,730 | 122,131 | 154,489 |
| 3 | 1 | 167,875 | 16,968 | 132,361 | 203,389 |
|  | 2 | 135,535 | 13,685 | 106,892 | 164,178 |
|  | 3 | 119,915 | 7,629 | 103,948 | 135,882 |
|  | 4 | 288,340 | 24,427 | 237,214 | 339,466 |
|  | 5 | 273,100 | 24,316 | 222,206 | 323,994 |
|  | 6 | 169,820 | 13,845 | 140,842 | 198,798 |
|  |  |  |  |  |  |

4. Ejerc \* Muscu

Comparaciones por parejasComparaciones por parejas, tabla, Medida, MEASURE\_1, 1 capas, 2 niveles de cabeceras de columna y 3 niveles de cabeceras de fila, tabla con 8 columnas y 97 filas

| |  |  |  | | --- | --- | --- | | Medida: | MEASURE\_1 | MEASURE\_1 | | | | | | | | |
|  |  |  |  |  |  |  |  |
| --- | --- | --- | --- | --- | --- | --- | --- |
| Ejerc | (I) Muscu | (J) Muscu | Diferencia de medias (I-J) | Desv. Error | Sig.b | 95% de intervalo de confianza para diferenciab | |
|  |  |  |  |  |  | Límite inferior | Límite superior |
| 1 | 1 | 2 | 18,580 | 7,681 | ,386 | -7,181 | 44,341 |
|  |  | 3 | 16,870 | 6,953 | ,381 | -6,451 | 40,191 |
|  |  | 4 | -65,655\* | 9,156 | ,000 | -96,365 | -34,945 |
|  |  | 5 | -81,540\* | 8,500 | ,000 | -110,051 | -53,029 |
|  |  | 6 | -69,255\* | 9,227 | ,000 | -100,202 | -38,308 |
|  | 2 | 1 | -18,580 | 7,681 | ,386 | -44,341 | 7,181 |
|  |  | 3 | -1,710 | 7,177 | 1,000 | -25,783 | 22,363 |
|  |  | 4 | -84,235\* | 9,173 | ,000 | -115,001 | -53,469 |
|  |  | 5 | -100,120\* | 12,344 | ,000 | -141,521 | -58,719 |
|  |  | 6 | -87,835\* | 11,583 | ,000 | -126,684 | -48,986 |
|  | 3 | 1 | -16,870 | 6,953 | ,381 | -40,191 | 6,451 |
|  |  | 2 | 1,710 | 7,177 | 1,000 | -22,363 | 25,783 |
|  |  | 4 | -82,525\* | 11,047 | ,000 | -119,578 | -45,472 |
|  |  | 5 | -98,410\* | 9,375 | ,000 | -129,854 | -66,966 |
|  |  | 6 | -86,125\* | 8,408 | ,000 | -114,327 | -57,923 |
|  | 4 | 1 | 65,655\* | 9,156 | ,000 | 34,945 | 96,365 |
|  |  | 2 | 84,235\* | 9,173 | ,000 | 53,469 | 115,001 |
|  |  | 3 | 82,525\* | 11,047 | ,000 | 45,472 | 119,578 |
|  |  | 5 | -15,885 | 12,132 | 1,000 | -56,576 | 24,806 |
|  |  | 6 | -3,600 | 13,789 | 1,000 | -49,850 | 42,650 |
|  | 5 | 1 | 81,540\* | 8,500 | ,000 | 53,029 | 110,051 |
|  |  | 2 | 100,120\* | 12,344 | ,000 | 58,719 | 141,521 |
|  |  | 3 | 98,410\* | 9,375 | ,000 | 66,966 | 129,854 |
|  |  | 4 | 15,885 | 12,132 | 1,000 | -24,806 | 56,576 |
|  |  | 6 | 12,285 | 11,491 | 1,000 | -26,255 | 50,825 |
|  | 6 | 1 | 69,255\* | 9,227 | ,000 | 38,308 | 100,202 |
|  |  | 2 | 87,835\* | 11,583 | ,000 | 48,986 | 126,684 |
|  |  | 3 | 86,125\* | 8,408 | ,000 | 57,923 | 114,327 |
|  |  | 4 | 3,600 | 13,789 | 1,000 | -42,650 | 49,850 |
|  |  | 5 | -12,285 | 11,491 | 1,000 | -50,825 | 26,255 |
| 2 | 1 | 2 | 8,365 | 7,661 | 1,000 | -17,332 | 34,062 |
|  |  | 3 | 13,075 | 6,344 | ,799 | -8,202 | 34,352 |
|  |  | 4 | -97,535\* | 11,707 | ,000 | -136,802 | -58,268 |
|  |  | 5 | -102,240\* | 14,448 | ,000 | -150,698 | -53,782 |
|  |  | 6 | -49,390\* | 7,269 | ,000 | -73,769 | -25,011 |
|  | 2 | 1 | -8,365 | 7,661 | 1,000 | -34,062 | 17,332 |
|  |  | 3 | 4,710 | 9,356 | 1,000 | -26,669 | 36,089 |
|  |  | 4 | -105,900\* | 10,770 | ,000 | -142,023 | -69,777 |
|  |  | 5 | -110,605\* | 16,481 | ,000 | -165,884 | -55,326 |
|  |  | 6 | -57,755\* | 10,259 | ,000 | -92,165 | -23,345 |
|  | 3 | 1 | -13,075 | 6,344 | ,799 | -34,352 | 8,202 |
|  |  | 2 | -4,710 | 9,356 | 1,000 | -36,089 | 26,669 |
|  |  | 4 | -110,610\* | 13,302 | ,000 | -155,225 | -65,995 |
|  |  | 5 | -115,315\* | 12,036 | ,000 | -155,683 | -74,947 |
|  |  | 6 | -62,465\* | 7,335 | ,000 | -87,068 | -37,862 |
|  | 4 | 1 | 97,535\* | 11,707 | ,000 | 58,268 | 136,802 |
|  |  | 2 | 105,900\* | 10,770 | ,000 | 69,777 | 142,023 |
|  |  | 3 | 110,610\* | 13,302 | ,000 | 65,995 | 155,225 |
|  |  | 5 | -4,705 | 18,607 | 1,000 | -67,115 | 57,705 |
|  |  | 6 | 48,145 | 14,836 | ,064 | -1,614 | 97,904 |
|  | 5 | 1 | 102,240\* | 14,448 | ,000 | 53,782 | 150,698 |
|  |  | 2 | 110,605\* | 16,481 | ,000 | 55,326 | 165,884 |
|  |  | 3 | 115,315\* | 12,036 | ,000 | 74,947 | 155,683 |
|  |  | 4 | 4,705 | 18,607 | 1,000 | -57,705 | 67,115 |
|  |  | 6 | 52,850\* | 13,914 | ,018 | 6,180 | 99,520 |
|  | 6 | 1 | 49,390\* | 7,269 | ,000 | 25,011 | 73,769 |
|  |  | 2 | 57,755\* | 10,259 | ,000 | 23,345 | 92,165 |
|  |  | 3 | 62,465\* | 7,335 | ,000 | 37,862 | 87,068 |
|  |  | 4 | -48,145 | 14,836 | ,064 | -97,904 | 1,614 |
|  |  | 5 | -52,850\* | 13,914 | ,018 | -99,520 | -6,180 |
| 3 | 1 | 2 | 32,340 | 15,293 | ,718 | -18,952 | 83,632 |
|  |  | 3 | 47,960 | 15,970 | ,110 | -5,604 | 101,524 |
|  |  | 4 | -120,465\* | 24,710 | ,002 | -203,342 | -37,588 |
|  |  | 5 | -105,225\* | 19,514 | ,001 | -170,674 | -39,776 |
|  |  | 6 | -1,945 | 16,230 | 1,000 | -56,381 | 52,491 |
|  | 2 | 1 | -32,340 | 15,293 | ,718 | -83,632 | 18,952 |
|  |  | 3 | 15,620 | 14,983 | 1,000 | -34,632 | 65,872 |
|  |  | 4 | -152,805\* | 19,786 | ,000 | -219,169 | -86,441 |
|  |  | 5 | -137,565\* | 22,522 | ,000 | -213,105 | -62,025 |
|  |  | 6 | -34,285 | 18,010 | 1,000 | -94,691 | 26,121 |
|  | 3 | 1 | -47,960 | 15,970 | ,110 | -101,524 | 5,604 |
|  |  | 2 | -15,620 | 14,983 | 1,000 | -65,872 | 34,632 |
|  |  | 4 | -168,425\* | 24,499 | ,000 | -250,596 | -86,254 |
|  |  | 5 | -153,185\* | 22,562 | ,000 | -228,857 | -77,513 |
|  |  | 6 | -49,905\* | 13,395 | ,021 | -94,832 | -4,978 |
|  | 4 | 1 | 120,465\* | 24,710 | ,002 | 37,588 | 203,342 |
|  |  | 2 | 152,805\* | 19,786 | ,000 | 86,441 | 219,169 |
|  |  | 3 | 168,425\* | 24,499 | ,000 | 86,254 | 250,596 |
|  |  | 5 | 15,240 | 28,700 | 1,000 | -81,022 | 111,502 |
|  |  | 6 | 118,520\* | 26,187 | ,003 | 30,687 | 206,353 |
|  | 5 | 1 | 105,225\* | 19,514 | ,001 | 39,776 | 170,674 |
|  |  | 2 | 137,565\* | 22,522 | ,000 | 62,025 | 213,105 |
|  |  | 3 | 153,185\* | 22,562 | ,000 | 77,513 | 228,857 |
|  |  | 4 | -15,240 | 28,700 | 1,000 | -111,502 | 81,022 |
|  |  | 6 | 103,280\* | 20,648 | ,001 | 34,024 | 172,536 |
|  | 6 | 1 | 1,945 | 16,230 | 1,000 | -52,491 | 56,381 |
|  |  | 2 | 34,285 | 18,010 | 1,000 | -26,121 | 94,691 |
|  |  | 3 | 49,905\* | 13,395 | ,021 | 4,978 | 94,832 |
|  |  | 4 | -118,520\* | 26,187 | ,003 | -206,353 | -30,687 |
|  |  | 5 | -103,280\* | 20,648 | ,001 | -172,536 | -34,024 |
|  |  |  |  |  |  |  |  |
| --- | --- | --- | --- | --- | --- | --- | --- |
| Se basa en medias marginales estimadas | | | | | | | |
| \*. La diferencia de medias es significativa en el nivel ,05. | | | | | | | |
| b. Ajuste para varias comparaciones: Bonferroni. | | | | | | | |
|  |  |  |  |  |  |  |  |

4. Ejerc \* Muscu

Pruebas multivariantePruebas multivariante, tabla, 1 niveles de cabeceras de columna y 2 niveles de cabeceras de fila, tabla con 10 columnas y 17 filas

|  |  |  |  |  |  |  |  |  |  |
| --- | --- | --- | --- | --- | --- | --- | --- | --- | --- |
| Ejerc | | Valor | F | gl de hipótesis | gl de error | Sig. | Eta parcial al cuadrado | Parámetro sin centralidad | Potencia observadab |
| 1 | Traza de Pillai | ,940 | 46,740a | 5,000 | 15,000 | ,000 | ,940 | 233,699 | 1,000 |
|  | Lambda de Wilks | ,060 | 46,740a | 5,000 | 15,000 | ,000 | ,940 | 233,699 | 1,000 |
|  | Traza de Hotelling | 15,580 | 46,740a | 5,000 | 15,000 | ,000 | ,940 | 233,699 | 1,000 |
|  | Raíz mayor de Roy | 15,580 | 46,740a | 5,000 | 15,000 | ,000 | ,940 | 233,699 | 1,000 |
| 2 | Traza de Pillai | ,938 | 45,370a | 5,000 | 15,000 | ,000 | ,938 | 226,848 | 1,000 |
|  | Lambda de Wilks | ,062 | 45,370a | 5,000 | 15,000 | ,000 | ,938 | 226,848 | 1,000 |
|  | Traza de Hotelling | 15,123 | 45,370a | 5,000 | 15,000 | ,000 | ,938 | 226,848 | 1,000 |
|  | Raíz mayor de Roy | 15,123 | 45,370a | 5,000 | 15,000 | ,000 | ,938 | 226,848 | 1,000 |
| 3 | Traza de Pillai | ,842 | 15,949a | 5,000 | 15,000 | ,000 | ,842 | 79,747 | 1,000 |
|  | Lambda de Wilks | ,158 | 15,949a | 5,000 | 15,000 | ,000 | ,842 | 79,747 | 1,000 |
|  | Traza de Hotelling | 5,316 | 15,949a | 5,000 | 15,000 | ,000 | ,842 | 79,747 | 1,000 |
|  | Raíz mayor de Roy | 5,316 | 15,949a | 5,000 | 15,000 | ,000 | ,842 | 79,747 | 1,000 |
|  |  |  |  |  |  |  |  |  |  |
| --- | --- | --- | --- | --- | --- | --- | --- | --- | --- |
| Cada F prueba los efectos simples multivariantes de Muscu dentro de cada combinación de niveles de los otros efectos mostrados. Estas pruebas se basan en las comparaciones por parejas linealmente independientes entre las medias marginales estimadas. | | | | | | | | | |
| a. Estadístico exacto | | | | | | | | | |
| b. Se ha calculado utilizando alpha = ,05 | | | | | | | | | |
|  |  |  |  |  |  |  |  |  |  |

4. Ejerc \* Muscu

Comparaciones por parejasComparaciones por parejas, tabla, Medida, MEASURE\_1, 1 capas, 2 niveles de cabeceras de columna y 3 niveles de cabeceras de fila, tabla con 8 columnas y 43 filas

| |  |  |  | | --- | --- | --- | | Medida: | MEASURE\_1 | MEASURE\_1 | | | | | | | | |
|  |  |  |  |  |  |  |  |
| --- | --- | --- | --- | --- | --- | --- | --- |
| Muscu | (I) Ejerc | (J) Ejerc | Diferencia de medias (I-J) | Desv. Error | Sig.b | 95% de intervalo de confianza para diferenciab | |
|  |  |  |  |  |  | Límite inferior | Límite superior |
| 1 | 1 | 2 | -,040 | 5,083 | 1,000 | -13,384 | 13,304 |
|  |  | 3 | -78,995\* | 12,873 | ,000 | -112,788 | -45,202 |
|  | 2 | 1 | ,040 | 5,083 | 1,000 | -13,304 | 13,384 |
|  |  | 3 | -78,955\* | 13,613 | ,000 | -114,690 | -43,220 |
|  | 3 | 1 | 78,995\* | 12,873 | ,000 | 45,202 | 112,788 |
|  |  | 2 | 78,955\* | 13,613 | ,000 | 43,220 | 114,690 |
| 2 | 1 | 2 | -10,255 | 5,276 | ,201 | -24,104 | 3,594 |
|  |  | 3 | -65,235\* | 10,031 | ,000 | -91,568 | -38,902 |
|  | 2 | 1 | 10,255 | 5,276 | ,201 | -3,594 | 24,104 |
|  |  | 3 | -54,980\* | 8,896 | ,000 | -78,334 | -31,626 |
|  | 3 | 1 | 65,235\* | 10,031 | ,000 | 38,902 | 91,568 |
|  |  | 2 | 54,980\* | 8,896 | ,000 | 31,626 | 78,334 |
| 3 | 1 | 2 | -3,835 | 4,710 | 1,000 | -16,199 | 8,529 |
|  |  | 3 | -47,905\* | 6,656 | ,000 | -65,377 | -30,433 |
|  | 2 | 1 | 3,835 | 4,710 | 1,000 | -8,529 | 16,199 |
|  |  | 3 | -44,070\* | 5,731 | ,000 | -59,115 | -29,025 |
|  | 3 | 1 | 47,905\* | 6,656 | ,000 | 30,433 | 65,377 |
|  |  | 2 | 44,070\* | 5,731 | ,000 | 29,025 | 59,115 |
| 4 | 1 | 2 | -31,920\* | 9,845 | ,013 | -57,765 | -6,075 |
|  |  | 3 | -133,805\* | 19,074 | ,000 | -183,877 | -83,733 |
|  | 2 | 1 | 31,920\* | 9,845 | ,013 | 6,075 | 57,765 |
|  |  | 3 | -101,885\* | 14,201 | ,000 | -139,165 | -64,605 |
|  | 3 | 1 | 133,805\* | 19,074 | ,000 | 83,733 | 183,877 |
|  |  | 2 | 101,885\* | 14,201 | ,000 | 64,605 | 139,165 |
| 5 | 1 | 2 | -20,740 | 10,587 | ,195 | -48,532 | 7,052 |
|  |  | 3 | -102,680\* | 15,747 | ,000 | -144,017 | -61,343 |
|  | 2 | 1 | 20,740 | 10,587 | ,195 | -7,052 | 48,532 |
|  |  | 3 | -81,940\* | 13,051 | ,000 | -116,200 | -47,680 |
|  | 3 | 1 | 102,680\* | 15,747 | ,000 | 61,343 | 144,017 |
|  |  | 2 | 81,940\* | 13,051 | ,000 | 47,680 | 116,200 |
| 6 | 1 | 2 | 19,825 | 8,483 | ,092 | -2,445 | 42,095 |
|  |  | 3 | -11,685 | 9,577 | ,712 | -36,824 | 13,454 |
|  | 2 | 1 | -19,825 | 8,483 | ,092 | -42,095 | 2,445 |
|  |  | 3 | -31,510\* | 9,627 | ,012 | -56,783 | -6,237 |
|  | 3 | 1 | 11,685 | 9,577 | ,712 | -13,454 | 36,824 |
|  |  | 2 | 31,510\* | 9,627 | ,012 | 6,237 | 56,783 |
|  |  |  |  |  |  |  |  |
| --- | --- | --- | --- | --- | --- | --- | --- |
| Se basa en medias marginales estimadas | | | | | | | |
| \*. La diferencia de medias es significativa en el nivel ,05. | | | | | | | |
| b. Ajuste para varias comparaciones: Bonferroni. | | | | | | | |
|  |  |  |  |  |  |  |  |

4. Ejerc \* Muscu

Pruebas multivariantePruebas multivariante, tabla, 1 niveles de cabeceras de columna y 2 niveles de cabeceras de fila, tabla con 10 columnas y 29 filas

|  |  |  |  |  |  |  |  |  |  |
| --- | --- | --- | --- | --- | --- | --- | --- | --- | --- |
| Muscu | | Valor | F | gl de hipótesis | gl de error | Sig. | Eta parcial al cuadrado | Parámetro sin centralidad | Potencia observadab |
| 1 | Traza de Pillai | ,665 | 17,876a | 2,000 | 18,000 | ,000 | ,665 | 35,752 | ,999 |
|  | Lambda de Wilks | ,335 | 17,876a | 2,000 | 18,000 | ,000 | ,665 | 35,752 | ,999 |
|  | Traza de Hotelling | 1,986 | 17,876a | 2,000 | 18,000 | ,000 | ,665 | 35,752 | ,999 |
|  | Raíz mayor de Roy | 1,986 | 17,876a | 2,000 | 18,000 | ,000 | ,665 | 35,752 | ,999 |
| 2 | Traza de Pillai | ,697 | 20,747a | 2,000 | 18,000 | ,000 | ,697 | 41,493 | 1,000 |
|  | Lambda de Wilks | ,303 | 20,747a | 2,000 | 18,000 | ,000 | ,697 | 41,493 | 1,000 |
|  | Traza de Hotelling | 2,305 | 20,747a | 2,000 | 18,000 | ,000 | ,697 | 41,493 | 1,000 |
|  | Raíz mayor de Roy | 2,305 | 20,747a | 2,000 | 18,000 | ,000 | ,697 | 41,493 | 1,000 |
| 3 | Traza de Pillai | ,773 | 30,716a | 2,000 | 18,000 | ,000 | ,773 | 61,431 | 1,000 |
|  | Lambda de Wilks | ,227 | 30,716a | 2,000 | 18,000 | ,000 | ,773 | 61,431 | 1,000 |
|  | Traza de Hotelling | 3,413 | 30,716a | 2,000 | 18,000 | ,000 | ,773 | 61,431 | 1,000 |
|  | Raíz mayor de Roy | 3,413 | 30,716a | 2,000 | 18,000 | ,000 | ,773 | 61,431 | 1,000 |
| 4 | Traza de Pillai | ,740 | 25,614a | 2,000 | 18,000 | ,000 | ,740 | 51,229 | 1,000 |
|  | Lambda de Wilks | ,260 | 25,614a | 2,000 | 18,000 | ,000 | ,740 | 51,229 | 1,000 |
|  | Traza de Hotelling | 2,846 | 25,614a | 2,000 | 18,000 | ,000 | ,740 | 51,229 | 1,000 |
|  | Raíz mayor de Roy | 2,846 | 25,614a | 2,000 | 18,000 | ,000 | ,740 | 51,229 | 1,000 |
| 5 | Traza de Pillai | ,712 | 22,289a | 2,000 | 18,000 | ,000 | ,712 | 44,578 | 1,000 |
|  | Lambda de Wilks | ,288 | 22,289a | 2,000 | 18,000 | ,000 | ,712 | 44,578 | 1,000 |
|  | Traza de Hotelling | 2,477 | 22,289a | 2,000 | 18,000 | ,000 | ,712 | 44,578 | 1,000 |
|  | Raíz mayor de Roy | 2,477 | 22,289a | 2,000 | 18,000 | ,000 | ,712 | 44,578 | 1,000 |
| 6 | Traza de Pillai | ,380 | 5,527a | 2,000 | 18,000 | ,013 | ,380 | 11,055 | ,786 |
|  | Lambda de Wilks | ,620 | 5,527a | 2,000 | 18,000 | ,013 | ,380 | 11,055 | ,786 |
|  | Traza de Hotelling | ,614 | 5,527a | 2,000 | 18,000 | ,013 | ,380 | 11,055 | ,786 |
|  | Raíz mayor de Roy | ,614 | 5,527a | 2,000 | 18,000 | ,013 | ,380 | 11,055 | ,786 |
|  |  |  |  |  |  |  |  |  |  |
| --- | --- | --- | --- | --- | --- | --- | --- | --- | --- |
| Cada F prueba los efectos simples multivariantes de Ejerc dentro de cada combinación de niveles de los otros efectos mostrados. Estas pruebas se basan en las comparaciones por parejas linealmente independientes entre las medias marginales estimadas. | | | | | | | | | |
| a. Estadístico exacto | | | | | | | | | |
| b. Se ha calculado utilizando alpha = ,05 | | | | | | | | | |
|  |  |  |  |  |  |  |  |  |  |

5. Ejerc \* act

EstimacionesEstimaciones, tabla, Medida, MEASURE\_1, 1 capas, 2 niveles de cabeceras de columna y 2 niveles de cabeceras de fila, tabla con 6 columnas y 10 filas

| |  |  |  | | --- | --- | --- | | Medida: | MEASURE\_1 | MEASURE\_1 | | | | | | |
|  |  |  |  |  |  |
| --- | --- | --- | --- | --- | --- |
| Ejerc | act | Media | Desv. Error | Intervalo de confianza al 95% | |
|  |  |  |  | Límite inferior | Límite superior |
| 1 | 1 | 153,167 | 9,481 | 133,323 | 173,010 |
|  | 2 | 84,927 | 3,595 | 77,403 | 92,451 |
| 2 | 1 | 144,083 | 8,740 | 125,791 | 162,376 |
|  | 2 | 109,665 | 4,486 | 100,276 | 119,054 |
| 3 | 1 | 213,817 | 14,314 | 183,858 | 243,775 |
|  | 2 | 171,045 | 9,787 | 150,561 | 191,529 |
|  |  |  |  |  |  |

5. Ejerc \* act

Comparaciones por parejasComparaciones por parejas, tabla, Medida, MEASURE\_1, 1 capas, 2 niveles de cabeceras de columna y 3 niveles de cabeceras de fila, tabla con 8 columnas y 13 filas

| |  |  |  | | --- | --- | --- | | Medida: | MEASURE\_1 | MEASURE\_1 | | | | | | | | |
|  |  |  |  |  |  |  |  |
| --- | --- | --- | --- | --- | --- | --- | --- |
| Ejerc | (I) act | (J) act | Diferencia de medias (I-J) | Desv. Error | Sig.b | 95% de intervalo de confianza para diferenciab | |
|  |  |  |  |  |  | Límite inferior | Límite superior |
| 1 | 1 | 2 | 68,240\* | 7,113 | ,000 | 53,352 | 83,128 |
|  | 2 | 1 | -68,240\* | 7,113 | ,000 | -83,128 | -53,352 |
| 2 | 1 | 2 | 34,418\* | 6,263 | ,000 | 21,311 | 47,526 |
|  | 2 | 1 | -34,418\* | 6,263 | ,000 | -47,526 | -21,311 |
| 3 | 1 | 2 | 42,772\* | 6,233 | ,000 | 29,726 | 55,817 |
|  | 2 | 1 | -42,772\* | 6,233 | ,000 | -55,817 | -29,726 |
|  |  |  |  |  |  |  |  |
| --- | --- | --- | --- | --- | --- | --- | --- |
| Se basa en medias marginales estimadas | | | | | | | |
| \*. La diferencia de medias es significativa en el nivel ,05. | | | | | | | |
| b. Ajuste para varias comparaciones: Bonferroni. | | | | | | | |
|  |  |  |  |  |  |  |  |

5. Ejerc \* act

Pruebas multivariantePruebas multivariante, tabla, 1 niveles de cabeceras de columna y 2 niveles de cabeceras de fila, tabla con 10 columnas y 17 filas

|  |  |  |  |  |  |  |  |  |  |
| --- | --- | --- | --- | --- | --- | --- | --- | --- | --- |
| Ejerc | | Valor | F | gl de hipótesis | gl de error | Sig. | Eta parcial al cuadrado | Parámetro sin centralidad | Potencia observadab |
| 1 | Traza de Pillai | ,829 | 92,032a | 1,000 | 19,000 | ,000 | ,829 | 92,032 | 1,000 |
|  | Lambda de Wilks | ,171 | 92,032a | 1,000 | 19,000 | ,000 | ,829 | 92,032 | 1,000 |
|  | Traza de Hotelling | 4,844 | 92,032a | 1,000 | 19,000 | ,000 | ,829 | 92,032 | 1,000 |
|  | Raíz mayor de Roy | 4,844 | 92,032a | 1,000 | 19,000 | ,000 | ,829 | 92,032 | 1,000 |
| 2 | Traza de Pillai | ,614 | 30,204a | 1,000 | 19,000 | ,000 | ,614 | 30,204 | ,999 |
|  | Lambda de Wilks | ,386 | 30,204a | 1,000 | 19,000 | ,000 | ,614 | 30,204 | ,999 |
|  | Traza de Hotelling | 1,590 | 30,204a | 1,000 | 19,000 | ,000 | ,614 | 30,204 | ,999 |
|  | Raíz mayor de Roy | 1,590 | 30,204a | 1,000 | 19,000 | ,000 | ,614 | 30,204 | ,999 |
| 3 | Traza de Pillai | ,713 | 47,090a | 1,000 | 19,000 | ,000 | ,713 | 47,090 | 1,000 |
|  | Lambda de Wilks | ,287 | 47,090a | 1,000 | 19,000 | ,000 | ,713 | 47,090 | 1,000 |
|  | Traza de Hotelling | 2,478 | 47,090a | 1,000 | 19,000 | ,000 | ,713 | 47,090 | 1,000 |
|  | Raíz mayor de Roy | 2,478 | 47,090a | 1,000 | 19,000 | ,000 | ,713 | 47,090 | 1,000 |
|  |  |  |  |  |  |  |  |  |  |
| --- | --- | --- | --- | --- | --- | --- | --- | --- | --- |
| Cada F prueba los efectos simples multivariantes de act dentro de cada combinación de niveles de los otros efectos mostrados. Estas pruebas se basan en las comparaciones por parejas linealmente independientes entre las medias marginales estimadas. | | | | | | | | | |
| a. Estadístico exacto | | | | | | | | | |
| b. Se ha calculado utilizando alpha = ,05 | | | | | | | | | |
|  |  |  |  |  |  |  |  |  |  |

5. Ejerc \* act

Comparaciones por parejasComparaciones por parejas, tabla, Medida, MEASURE\_1, 1 capas, 2 niveles de cabeceras de columna y 3 niveles de cabeceras de fila, tabla con 8 columnas y 19 filas

| |  |  |  | | --- | --- | --- | | Medida: | MEASURE\_1 | MEASURE\_1 | | | | | | | | |
|  |  |  |  |  |  |  |  |
| --- | --- | --- | --- | --- | --- | --- | --- |
| act | (I) Ejerc | (J) Ejerc | Diferencia de medias (I-J) | Desv. Error | Sig.b | 95% de intervalo de confianza para diferenciab | |
|  |  |  |  |  |  | Límite inferior | Límite superior |
| 1 | 1 | 2 | 9,083 | 7,104 | ,649 | -9,565 | 27,731 |
|  |  | 3 | -60,650\* | 8,822 | ,000 | -83,810 | -37,490 |
|  | 2 | 1 | -9,083 | 7,104 | ,649 | -27,731 | 9,565 |
|  |  | 3 | -69,733\* | 7,634 | ,000 | -89,774 | -49,693 |
|  | 3 | 1 | 60,650\* | 8,822 | ,000 | 37,490 | 83,810 |
|  |  | 2 | 69,733\* | 7,634 | ,000 | 49,693 | 89,774 |
| 2 | 1 | 2 | -24,738\* | 4,204 | ,000 | -35,775 | -13,701 |
|  |  | 3 | -86,118\* | 8,386 | ,000 | -108,131 | -64,105 |
|  | 2 | 1 | 24,738\* | 4,204 | ,000 | 13,701 | 35,775 |
|  |  | 3 | -61,380\* | 8,149 | ,000 | -82,773 | -39,987 |
|  | 3 | 1 | 86,118\* | 8,386 | ,000 | 64,105 | 108,131 |
|  |  | 2 | 61,380\* | 8,149 | ,000 | 39,987 | 82,773 |
|  |  |  |  |  |  |  |  |
| --- | --- | --- | --- | --- | --- | --- | --- |
| Se basa en medias marginales estimadas | | | | | | | |
| \*. La diferencia de medias es significativa en el nivel ,05. | | | | | | | |
| b. Ajuste para varias comparaciones: Bonferroni. | | | | | | | |
|  |  |  |  |  |  |  |  |

5. Ejerc \* act

Pruebas multivariantePruebas multivariante, tabla, 1 niveles de cabeceras de columna y 2 niveles de cabeceras de fila, tabla con 10 columnas y 13 filas

|  |  |  |  |  |  |  |  |  |  |
| --- | --- | --- | --- | --- | --- | --- | --- | --- | --- |
| act | | Valor | F | gl de hipótesis | gl de error | Sig. | Eta parcial al cuadrado | Parámetro sin centralidad | Potencia observadab |
| 1 | Traza de Pillai | ,818 | 40,427a | 2,000 | 18,000 | ,000 | ,818 | 80,854 | 1,000 |
|  | Lambda de Wilks | ,182 | 40,427a | 2,000 | 18,000 | ,000 | ,818 | 80,854 | 1,000 |
|  | Traza de Hotelling | 4,492 | 40,427a | 2,000 | 18,000 | ,000 | ,818 | 80,854 | 1,000 |
|  | Raíz mayor de Roy | 4,492 | 40,427a | 2,000 | 18,000 | ,000 | ,818 | 80,854 | 1,000 |
| 2 | Traza de Pillai | ,857 | 53,883a | 2,000 | 18,000 | ,000 | ,857 | 107,766 | 1,000 |
|  | Lambda de Wilks | ,143 | 53,883a | 2,000 | 18,000 | ,000 | ,857 | 107,766 | 1,000 |
|  | Traza de Hotelling | 5,987 | 53,883a | 2,000 | 18,000 | ,000 | ,857 | 107,766 | 1,000 |
|  | Raíz mayor de Roy | 5,987 | 53,883a | 2,000 | 18,000 | ,000 | ,857 | 107,766 | 1,000 |
|  |  |  |  |  |  |  |  |  |  |
| --- | --- | --- | --- | --- | --- | --- | --- | --- | --- |
| Cada F prueba los efectos simples multivariantes de Ejerc dentro de cada combinación de niveles de los otros efectos mostrados. Estas pruebas se basan en las comparaciones por parejas linealmente independientes entre las medias marginales estimadas. | | | | | | | | | |
| a. Estadístico exacto | | | | | | | | | |
| b. Se ha calculado utilizando alpha = ,05 | | | | | | | | | |
|  |  |  |  |  |  |  |  |  |  |

6. Muscu \* act

EstimacionesEstimaciones, tabla, Medida, MEASURE\_1, 1 capas, 2 niveles de cabeceras de columna y 2 niveles de cabeceras de fila, tabla con 6 columnas y 16 filas

| |  |  |  | | --- | --- | --- | | Medida: | MEASURE\_1 | MEASURE\_1 | | | | | | |
|  |  |  |  |  |  |
| --- | --- | --- | --- | --- | --- |
| Muscu | act | Media | Desv. Error | Intervalo de confianza al 95% | |
|  |  |  |  | Límite inferior | Límite superior |
| 1 | 1 | 139,367 | 12,513 | 113,177 | 165,556 |
|  | 2 | 91,083 | 6,620 | 77,227 | 104,939 |
| 2 | 1 | 124,127 | 13,189 | 96,522 | 151,731 |
|  | 2 | 66,800 | 4,784 | 56,787 | 76,813 |
| 3 | 1 | 106,410 | 6,775 | 92,229 | 120,591 |
|  | 2 | 72,103 | 3,916 | 63,906 | 80,301 |
| 4 | 1 | 240,110 | 17,827 | 202,798 | 277,422 |
|  | 2 | 179,443 | 12,317 | 153,663 | 205,223 |
| 5 | 1 | 237,277 | 19,374 | 196,727 | 277,826 |
|  | 2 | 185,843 | 13,862 | 156,830 | 214,857 |
| 6 | 1 | 174,843 | 11,405 | 150,972 | 198,714 |
|  | 2 | 136,000 | 8,093 | 119,061 | 152,939 |
|  |  |  |  |  |  |

6. Muscu \* act

Comparaciones por parejasComparaciones por parejas, tabla, Medida, MEASURE\_1, 1 capas, 2 niveles de cabeceras de columna y 3 niveles de cabeceras de fila, tabla con 8 columnas y 19 filas

| |  |  |  | | --- | --- | --- | | Medida: | MEASURE\_1 | MEASURE\_1 | | | | | | | | |
|  |  |  |  |  |  |  |  |
| --- | --- | --- | --- | --- | --- | --- | --- |
| Muscu | (I) act | (J) act | Diferencia de medias (I-J) | Desv. Error | Sig.b | 95% de intervalo de confianza para diferenciab | |
|  |  |  |  |  |  | Límite inferior | Límite superior |
| 1 | 1 | 2 | 48,283\* | 7,263 | ,000 | 33,082 | 63,485 |
|  | 2 | 1 | -48,283\* | 7,263 | ,000 | -63,485 | -33,082 |
| 2 | 1 | 2 | 57,327\* | 9,777 | ,000 | 36,863 | 77,790 |
|  | 2 | 1 | -57,327\* | 9,777 | ,000 | -77,790 | -36,863 |
| 3 | 1 | 2 | 34,307\* | 4,831 | ,000 | 24,196 | 44,418 |
|  | 2 | 1 | -34,307\* | 4,831 | ,000 | -44,418 | -24,196 |
| 4 | 1 | 2 | 60,667\* | 6,845 | ,000 | 46,340 | 74,993 |
|  | 2 | 1 | -60,667\* | 6,845 | ,000 | -74,993 | -46,340 |
| 5 | 1 | 2 | 51,433\* | 8,655 | ,000 | 33,318 | 69,548 |
|  | 2 | 1 | -51,433\* | 8,655 | ,000 | -69,548 | -33,318 |
| 6 | 1 | 2 | 38,843\* | 6,206 | ,000 | 25,854 | 51,833 |
|  | 2 | 1 | -38,843\* | 6,206 | ,000 | -51,833 | -25,854 |
|  |  |  |  |  |  |  |  |
| --- | --- | --- | --- | --- | --- | --- | --- |
| Se basa en medias marginales estimadas | | | | | | | |
| \*. La diferencia de medias es significativa en el nivel ,05. | | | | | | | |
| b. Ajuste para varias comparaciones: Bonferroni. | | | | | | | |
|  |  |  |  |  |  |  |  |

6. Muscu \* act

Pruebas multivariantePruebas multivariante, tabla, 1 niveles de cabeceras de columna y 2 niveles de cabeceras de fila, tabla con 10 columnas y 29 filas

|  |  |  |  |  |  |  |  |  |  |
| --- | --- | --- | --- | --- | --- | --- | --- | --- | --- |
| Muscu | | Valor | F | gl de hipótesis | gl de error | Sig. | Eta parcial al cuadrado | Parámetro sin centralidad | Potencia observadab |
| 1 | Traza de Pillai | ,699 | 44,196a | 1,000 | 19,000 | ,000 | ,699 | 44,196 | 1,000 |
|  | Lambda de Wilks | ,301 | 44,196a | 1,000 | 19,000 | ,000 | ,699 | 44,196 | 1,000 |
|  | Traza de Hotelling | 2,326 | 44,196a | 1,000 | 19,000 | ,000 | ,699 | 44,196 | 1,000 |
|  | Raíz mayor de Roy | 2,326 | 44,196a | 1,000 | 19,000 | ,000 | ,699 | 44,196 | 1,000 |
| 2 | Traza de Pillai | ,644 | 34,379a | 1,000 | 19,000 | ,000 | ,644 | 34,379 | 1,000 |
|  | Lambda de Wilks | ,356 | 34,379a | 1,000 | 19,000 | ,000 | ,644 | 34,379 | 1,000 |
|  | Traza de Hotelling | 1,809 | 34,379a | 1,000 | 19,000 | ,000 | ,644 | 34,379 | 1,000 |
|  | Raíz mayor de Roy | 1,809 | 34,379a | 1,000 | 19,000 | ,000 | ,644 | 34,379 | 1,000 |
| 3 | Traza de Pillai | ,726 | 50,434a | 1,000 | 19,000 | ,000 | ,726 | 50,434 | 1,000 |
|  | Lambda de Wilks | ,274 | 50,434a | 1,000 | 19,000 | ,000 | ,726 | 50,434 | 1,000 |
|  | Traza de Hotelling | 2,654 | 50,434a | 1,000 | 19,000 | ,000 | ,726 | 50,434 | 1,000 |
|  | Raíz mayor de Roy | 2,654 | 50,434a | 1,000 | 19,000 | ,000 | ,726 | 50,434 | 1,000 |
| 4 | Traza de Pillai | ,805 | 78,557a | 1,000 | 19,000 | ,000 | ,805 | 78,557 | 1,000 |
|  | Lambda de Wilks | ,195 | 78,557a | 1,000 | 19,000 | ,000 | ,805 | 78,557 | 1,000 |
|  | Traza de Hotelling | 4,135 | 78,557a | 1,000 | 19,000 | ,000 | ,805 | 78,557 | 1,000 |
|  | Raíz mayor de Roy | 4,135 | 78,557a | 1,000 | 19,000 | ,000 | ,805 | 78,557 | 1,000 |
| 5 | Traza de Pillai | ,650 | 35,315a | 1,000 | 19,000 | ,000 | ,650 | 35,315 | 1,000 |
|  | Lambda de Wilks | ,350 | 35,315a | 1,000 | 19,000 | ,000 | ,650 | 35,315 | 1,000 |
|  | Traza de Hotelling | 1,859 | 35,315a | 1,000 | 19,000 | ,000 | ,650 | 35,315 | 1,000 |
|  | Raíz mayor de Roy | 1,859 | 35,315a | 1,000 | 19,000 | ,000 | ,650 | 35,315 | 1,000 |
| 6 | Traza de Pillai | ,673 | 39,174a | 1,000 | 19,000 | ,000 | ,673 | 39,174 | 1,000 |
|  | Lambda de Wilks | ,327 | 39,174a | 1,000 | 19,000 | ,000 | ,673 | 39,174 | 1,000 |
|  | Traza de Hotelling | 2,062 | 39,174a | 1,000 | 19,000 | ,000 | ,673 | 39,174 | 1,000 |
|  | Raíz mayor de Roy | 2,062 | 39,174a | 1,000 | 19,000 | ,000 | ,673 | 39,174 | 1,000 |
|  |  |  |  |  |  |  |  |  |  |
| --- | --- | --- | --- | --- | --- | --- | --- | --- | --- |
| Cada F prueba los efectos simples multivariantes de act dentro de cada combinación de niveles de los otros efectos mostrados. Estas pruebas se basan en las comparaciones por parejas linealmente independientes entre las medias marginales estimadas. | | | | | | | | | |
| a. Estadístico exacto | | | | | | | | | |
| b. Se ha calculado utilizando alpha = ,05 | | | | | | | | | |
|  |  |  |  |  |  |  |  |  |  |

6. Muscu \* act

Comparaciones por parejasComparaciones por parejas, tabla, Medida, MEASURE\_1, 1 capas, 2 niveles de cabeceras de columna y 3 niveles de cabeceras de fila, tabla con 8 columnas y 67 filas

| |  |  |  | | --- | --- | --- | | Medida: | MEASURE\_1 | MEASURE\_1 | | | | | | | | |
|  |  |  |  |  |  |  |  |
| --- | --- | --- | --- | --- | --- | --- | --- |
| act | (I) Muscu | (J) Muscu | Diferencia de medias (I-J) | Desv. Error | Sig.b | 95% de intervalo de confianza para diferenciab | |
|  |  |  |  |  |  | Límite inferior | Límite superior |
| 1 | 1 | 2 | 15,240 | 11,771 | 1,000 | -24,239 | 54,719 |
|  |  | 3 | 32,957 | 10,319 | ,072 | -1,652 | 67,565 |
|  |  | 4 | -100,743\* | 16,071 | ,000 | -154,646 | -46,841 |
|  |  | 5 | -97,910\* | 14,666 | ,000 | -147,101 | -48,719 |
|  |  | 6 | -35,477 | 10,601 | ,051 | -71,032 | ,078 |
|  | 2 | 1 | -15,240 | 11,771 | 1,000 | -54,719 | 24,239 |
|  |  | 3 | 17,717 | 13,602 | 1,000 | -27,906 | 63,340 |
|  |  | 4 | -115,983\* | 13,535 | ,000 | -161,381 | -70,586 |
|  |  | 5 | -113,150\* | 19,491 | ,000 | -178,525 | -47,775 |
|  |  | 6 | -50,717\* | 15,042 | ,048 | -101,167 | -,267 |
|  | 3 | 1 | -32,957 | 10,319 | ,072 | -67,565 | 1,652 |
|  |  | 2 | -17,717 | 13,602 | 1,000 | -63,340 | 27,906 |
|  |  | 4 | -133,700\* | 17,249 | ,000 | -191,553 | -75,847 |
|  |  | 5 | -130,867\* | 15,406 | ,000 | -182,539 | -79,195 |
|  |  | 6 | -68,433\* | 9,726 | ,000 | -101,054 | -35,813 |
|  | 4 | 1 | 100,743\* | 16,071 | ,000 | 46,841 | 154,646 |
|  |  | 2 | 115,983\* | 13,535 | ,000 | 70,586 | 161,381 |
|  |  | 3 | 133,700\* | 17,249 | ,000 | 75,847 | 191,553 |
|  |  | 5 | 2,833 | 21,559 | 1,000 | -69,475 | 75,141 |
|  |  | 6 | 65,267\* | 18,829 | ,039 | 2,113 | 128,421 |
|  | 5 | 1 | 97,910\* | 14,666 | ,000 | 48,719 | 147,101 |
|  |  | 2 | 113,150\* | 19,491 | ,000 | 47,775 | 178,525 |
|  |  | 3 | 130,867\* | 15,406 | ,000 | 79,195 | 182,539 |
|  |  | 4 | -2,833 | 21,559 | 1,000 | -75,141 | 69,475 |
|  |  | 6 | 62,433\* | 15,375 | ,010 | 10,864 | 114,003 |
|  | 6 | 1 | 35,477 | 10,601 | ,051 | -,078 | 71,032 |
|  |  | 2 | 50,717\* | 15,042 | ,048 | ,267 | 101,167 |
|  |  | 3 | 68,433\* | 9,726 | ,000 | 35,813 | 101,054 |
|  |  | 4 | -65,267\* | 18,829 | ,039 | -128,421 | -2,113 |
|  |  | 5 | -62,433\* | 15,375 | ,010 | -114,003 | -10,864 |
| 2 | 1 | 2 | 24,283\* | 6,641 | ,025 | 2,010 | 46,556 |
|  |  | 3 | 18,980 | 6,915 | ,193 | -4,214 | 42,174 |
|  |  | 4 | -88,360\* | 12,133 | ,000 | -129,055 | -47,665 |
|  |  | 5 | -94,760\* | 12,139 | ,000 | -135,475 | -54,045 |
|  |  | 6 | -44,917\* | 8,664 | ,001 | -73,977 | -15,856 |
|  | 2 | 1 | -24,283\* | 6,641 | ,025 | -46,556 | -2,010 |
|  |  | 3 | -5,303 | 6,176 | 1,000 | -26,017 | 15,411 |
|  |  | 4 | -112,643\* | 11,094 | ,000 | -149,852 | -75,434 |
|  |  | 5 | -119,043\* | 14,154 | ,000 | -166,517 | -71,569 |
|  |  | 6 | -69,200\* | 9,216 | ,000 | -100,111 | -38,289 |
|  | 3 | 1 | -18,980 | 6,915 | ,193 | -42,174 | 4,214 |
|  |  | 2 | 5,303 | 6,176 | 1,000 | -15,411 | 26,017 |
|  |  | 4 | -107,340\* | 14,056 | ,000 | -154,485 | -60,195 |
|  |  | 5 | -113,740\* | 12,827 | ,000 | -156,762 | -70,718 |
|  |  | 6 | -63,897\* | 7,382 | ,000 | -88,655 | -39,138 |
|  | 4 | 1 | 88,360\* | 12,133 | ,000 | 47,665 | 129,055 |
|  |  | 2 | 112,643\* | 11,094 | ,000 | 75,434 | 149,852 |
|  |  | 3 | 107,340\* | 14,056 | ,000 | 60,195 | 154,485 |
|  |  | 5 | -6,400 | 16,941 | 1,000 | -63,219 | 50,419 |
|  |  | 6 | 43,443 | 15,222 | ,152 | -7,611 | 94,498 |
|  | 5 | 1 | 94,760\* | 12,139 | ,000 | 54,045 | 135,475 |
|  |  | 2 | 119,043\* | 14,154 | ,000 | 71,569 | 166,517 |
|  |  | 3 | 113,740\* | 12,827 | ,000 | 70,718 | 156,762 |
|  |  | 4 | 6,400 | 16,941 | 1,000 | -50,419 | 63,219 |
|  |  | 6 | 49,843\* | 13,298 | ,020 | 5,241 | 94,446 |
|  | 6 | 1 | 44,917\* | 8,664 | ,001 | 15,856 | 73,977 |
|  |  | 2 | 69,200\* | 9,216 | ,000 | 38,289 | 100,111 |
|  |  | 3 | 63,897\* | 7,382 | ,000 | 39,138 | 88,655 |
|  |  | 4 | -43,443 | 15,222 | ,152 | -94,498 | 7,611 |
|  |  | 5 | -49,843\* | 13,298 | ,020 | -94,446 | -5,241 |
|  |  |  |  |  |  |  |  |
| --- | --- | --- | --- | --- | --- | --- | --- |
| Se basa en medias marginales estimadas | | | | | | | |
| \*. La diferencia de medias es significativa en el nivel ,05. | | | | | | | |
| b. Ajuste para varias comparaciones: Bonferroni. | | | | | | | |
|  |  |  |  |  |  |  |  |

6. Muscu \* act

Pruebas multivariantePruebas multivariante, tabla, 1 niveles de cabeceras de columna y 2 niveles de cabeceras de fila, tabla con 10 columnas y 13 filas

|  |  |  |  |  |  |  |  |  |  |
| --- | --- | --- | --- | --- | --- | --- | --- | --- | --- |
| act | | Valor | F | gl de hipótesis | gl de error | Sig. | Eta parcial al cuadrado | Parámetro sin centralidad | Potencia observadab |
| 1 | Traza de Pillai | ,902 | 27,550a | 5,000 | 15,000 | ,000 | ,902 | 137,751 | 1,000 |
|  | Lambda de Wilks | ,098 | 27,550a | 5,000 | 15,000 | ,000 | ,902 | 137,751 | 1,000 |
|  | Traza de Hotelling | 9,183 | 27,550a | 5,000 | 15,000 | ,000 | ,902 | 137,751 | 1,000 |
|  | Raíz mayor de Roy | 9,183 | 27,550a | 5,000 | 15,000 | ,000 | ,902 | 137,751 | 1,000 |
| 2 | Traza de Pillai | ,919 | 33,952a | 5,000 | 15,000 | ,000 | ,919 | 169,762 | 1,000 |
|  | Lambda de Wilks | ,081 | 33,952a | 5,000 | 15,000 | ,000 | ,919 | 169,762 | 1,000 |
|  | Traza de Hotelling | 11,317 | 33,952a | 5,000 | 15,000 | ,000 | ,919 | 169,762 | 1,000 |
|  | Raíz mayor de Roy | 11,317 | 33,952a | 5,000 | 15,000 | ,000 | ,919 | 169,762 | 1,000 |
|  |  |  |  |  |  |  |  |  |  |
| --- | --- | --- | --- | --- | --- | --- | --- | --- | --- |
| Cada F prueba los efectos simples multivariantes de Muscu dentro de cada combinación de niveles de los otros efectos mostrados. Estas pruebas se basan en las comparaciones por parejas linealmente independientes entre las medias marginales estimadas. | | | | | | | | | |
| a. Estadístico exacto | | | | | | | | | |
| b. Se ha calculado utilizando alpha = ,05 | | | | | | | | | |
|  |  |  |  |  |  |  |  |  |  |

7. Ejerc \* Muscu \* act

EstimacionesEstimaciones, tabla, Medida, MEASURE\_1, 1 capas, 2 niveles de cabeceras de columna y 3 niveles de cabeceras de fila, tabla con 7 columnas y 40 filas

| |  |  |  | | --- | --- | --- | | Medida: | MEASURE\_1 | MEASURE\_1 | | | | | | | |
|  |  |  |  |  |  |  |
| --- | --- | --- | --- | --- | --- | --- |
| Ejerc | Muscu | act | Media | Desv. Error | Intervalo de confianza al 95% | |
|  |  |  |  |  | Límite inferior | Límite superior |
| 1 | 1 | 1 | 117,170 | 12,771 | 90,441 | 143,899 |
|  |  | 2 | 60,590 | 4,981 | 50,165 | 71,015 |
|  | 2 | 1 | 97,610 | 11,278 | 74,005 | 121,215 |
|  |  | 2 | 42,990 | 5,789 | 30,873 | 55,107 |
|  | 3 | 1 | 92,170 | 6,316 | 78,951 | 105,389 |
|  |  | 2 | 51,850 | 3,151 | 45,255 | 58,445 |
|  | 4 | 1 | 197,340 | 14,882 | 166,192 | 228,488 |
|  |  | 2 | 111,730 | 7,313 | 96,424 | 127,036 |
|  | 5 | 1 | 216,710 | 16,820 | 181,505 | 251,915 |
|  |  | 2 | 124,130 | 7,618 | 108,185 | 140,075 |
|  | 6 | 1 | 198,000 | 13,163 | 170,450 | 225,550 |
|  |  | 2 | 118,270 | 7,883 | 101,772 | 134,768 |
| 2 | 1 | 1 | 106,250 | 7,709 | 90,114 | 122,386 |
|  |  | 2 | 71,590 | 4,298 | 62,593 | 80,587 |
|  | 2 | 1 | 106,090 | 11,485 | 82,052 | 130,128 |
|  |  | 2 | 55,020 | 4,353 | 45,910 | 64,130 |
|  | 3 | 1 | 88,770 | 6,684 | 74,781 | 102,759 |
|  |  | 2 | 62,920 | 5,355 | 51,711 | 74,129 |
|  | 4 | 1 | 208,180 | 16,345 | 173,970 | 242,390 |
|  |  | 2 | 164,730 | 10,933 | 141,847 | 187,613 |
|  | 5 | 1 | 206,820 | 18,897 | 167,268 | 246,372 |
|  |  | 2 | 175,500 | 14,037 | 146,119 | 204,881 |
|  | 6 | 1 | 148,390 | 9,546 | 128,410 | 168,370 |
|  |  | 2 | 128,230 | 7,703 | 112,107 | 144,353 |
| 3 | 1 | 1 | 194,680 | 20,253 | 152,290 | 237,070 |
|  |  | 2 | 141,070 | 14,152 | 111,450 | 170,690 |
|  | 2 | 1 | 168,680 | 19,108 | 128,687 | 208,673 |
|  |  | 2 | 102,390 | 8,777 | 84,020 | 120,760 |
|  | 3 | 1 | 138,290 | 10,414 | 116,493 | 160,087 |
|  |  | 2 | 101,540 | 6,627 | 87,670 | 115,410 |
|  | 4 | 1 | 314,810 | 27,321 | 257,627 | 371,993 |
|  |  | 2 | 261,870 | 22,177 | 215,453 | 308,287 |
|  | 5 | 1 | 288,300 | 26,427 | 232,989 | 343,611 |
|  |  | 2 | 257,900 | 23,728 | 208,237 | 307,563 |
|  | 6 | 1 | 178,140 | 15,356 | 145,999 | 210,281 |
|  |  | 2 | 161,500 | 12,890 | 134,521 | 188,479 |
|  |  |  |  |  |  |  |

7. Ejerc \* Muscu \* act

Comparaciones por parejasComparaciones por parejas, tabla, Medida, MEASURE\_1, 1 capas, 2 niveles de cabeceras de columna y 4 niveles de cabeceras de fila, tabla con 9 columnas y 43 filas

| |  |  |  | | --- | --- | --- | | Medida: | MEASURE\_1 | MEASURE\_1 | | | | | | | | | |  |
|  |  |  |  |  |  |  |  |  |  |
| --- | --- | --- | --- | --- | --- | --- | --- | --- | --- |
| Ejerc | Muscu | (I) act | (J) act | Diferencia de medias (I-J) | Desv. Error | Sig.b | 95% de intervalo de confianza para diferenciab | |  |
|  |  |  |  |  |  |  | Límite inferior | Límite superior |  |
| 1 | 1 | 1 | 2 | 56,580\* | 10,403 | ,000 | 34,807 | 78,353 |  |
|  |  | 2 | 1 | -56,580\* | 10,403 | ,000 | -78,353 | -34,807 |  |
|  | 2 | 1 | 2 | 54,620\* | 10,922 | ,000 | 31,760 | 77,480 |  |
|  |  | 2 | 1 | -54,620\* | 10,922 | ,000 | -77,480 | -31,760 |  |
|  | 3 | 1 | 2 | 40,320\* | 5,204 | ,000 | 29,427 | 51,213 |  |
|  |  | 2 | 1 | -40,320\* | 5,204 | ,000 | -51,213 | -29,427 |  |
|  | 4 | 1 | 2 | 85,610\* | 9,114 | ,000 | 66,534 | 104,686 |  |
|  |  | 2 | 1 | -85,610\* | 9,114 | ,000 | -104,686 | -66,534 |  |
|  | 5 | 1 | 2 | 92,580\* | 11,875 | ,000 | 67,726 | 117,434 |  |
|  |  | 2 | 1 | -92,580\* | 11,875 | ,000 | -117,434 | -67,726 |  |
|  | 6 | 1 | 2 | 79,730\* | 8,763 | ,000 | 61,388 | 98,072 |  |
|  |  | 2 | 1 | -79,730\* | 8,763 | ,000 | -98,072 | -61,388 |  |
| 2 | 1 | 1 | 2 | 34,660\* | 5,327 | ,000 | 23,511 | 45,809 |  |
|  |  | 2 | 1 | -34,660\* | 5,327 | ,000 | -45,809 | -23,511 |  |
|  | 2 | 1 | 2 | 51,070\* | 9,048 | ,000 | 32,132 | 70,008 |  |
|  |  | 2 | 1 | -51,070\* | 9,048 | ,000 | -70,008 | -32,132 |  |
|  | 3 | 1 | 2 | 25,850\* | 4,707 | ,000 | 15,999 | 35,701 |  |
|  |  | 2 | 1 | -25,850\* | 4,707 | ,000 | -35,701 | -15,999 |  |
|  | 4 | 1 | 2 | 43,450\* | 8,198 | ,000 | 26,291 | 60,609 |  |
|  |  | 2 | 1 | -43,450\* | 8,198 | ,000 | -60,609 | -26,291 |  |
|  | 5 | 1 | 2 | 31,320\* | 11,261 | ,012 | 7,749 | 54,891 |  |
|  |  | 2 | 1 | -31,320\* | 11,261 | ,012 | -54,891 | -7,749 |  |
|  | 6 | 1 | 2 | 20,160\* | 7,868 | ,019 | 3,691 | 36,629 |  |
|  |  | 2 | 1 | -20,160\* | 7,868 | ,019 | -36,629 | -3,691 |  |
| 3 | 1 | 1 | 2 | 53,610\* | 8,324 | ,000 | 36,189 | 71,031 |  |
|  |  | 2 | 1 | -53,610\* | 8,324 | ,000 | -71,031 | -36,189 |  |
|  | 2 | 1 | 2 | 66,290\* | 11,626 | ,000 | 41,957 | 90,623 |  |
|  |  | 2 | 1 | -66,290\* | 11,626 | ,000 | -90,623 | -41,957 |  |
|  | 3 | 1 | 2 | 36,750\* | 8,482 | ,000 | 18,996 | 54,504 |  |
|  |  | 2 | 1 | -36,750\* | 8,482 | ,000 | -54,504 | -18,996 |  |
|  | 4 | 1 | 2 | 52,940\* | 9,475 | ,000 | 33,109 | 72,771 |  |
|  |  | 2 | 1 | -52,940\* | 9,475 | ,000 | -72,771 | -33,109 |  |
|  | 5 | 1 | 2 | 30,400\* | 12,559 | ,026 | 4,113 | 56,687 |  |
|  |  | 2 | 1 | -30,400\* | 12,559 | ,026 | -56,687 | -4,113 |  |
|  | 6 | 1 | 2 | 16,640\* | 6,101 | ,013 | 3,871 | 29,409 |  |
|  |  | 2 | 1 | -16,640\* | 6,101 | ,013 | -29,409 | -3,871 |  |
|  |  |  |  |  |  |  |  |  |  |
| --- | --- | --- | --- | --- | --- | --- | --- | --- | --- |
| Se basa en medias marginales estimadas | | | | | | | | |  |
| \*. La diferencia de medias es significativa en el nivel ,05. | | | | | | | | |  |
| b. Ajuste para varias comparaciones: Bonferroni. | | | | | | | | |  |
|  |  |  |  |  |  |  |  |  |  |

7. Ejerc \* Muscu \* act

Pruebas multivariantePruebas multivariante, tabla, 1 niveles de cabeceras de columna y 3 niveles de cabeceras de fila, tabla con 11 columnas y 77 filas

|  |  |  |  |  |  |  |  |  |  |  |
| --- | --- | --- | --- | --- | --- | --- | --- | --- | --- | --- |
| Ejerc | Muscu | | Valor | F | gl de hipótesis | gl de error | Sig. | Eta parcial al cuadrado | Parámetro sin centralidad | Potencia observadab |
| 1 | 1 | Traza de Pillai | ,609 | 29,581a | 1,000 | 19,000 | ,000 | ,609 | 29,581 | ,999 |
|  |  | Lambda de Wilks | ,391 | 29,581a | 1,000 | 19,000 | ,000 | ,609 | 29,581 | ,999 |
|  |  | Traza de Hotelling | 1,557 | 29,581a | 1,000 | 19,000 | ,000 | ,609 | 29,581 | ,999 |
|  |  | Raíz mayor de Roy | 1,557 | 29,581a | 1,000 | 19,000 | ,000 | ,609 | 29,581 | ,999 |
|  | 2 | Traza de Pillai | ,568 | 25,010a | 1,000 | 19,000 | ,000 | ,568 | 25,010 | ,997 |
|  |  | Lambda de Wilks | ,432 | 25,010a | 1,000 | 19,000 | ,000 | ,568 | 25,010 | ,997 |
|  |  | Traza de Hotelling | 1,316 | 25,010a | 1,000 | 19,000 | ,000 | ,568 | 25,010 | ,997 |
|  |  | Raíz mayor de Roy | 1,316 | 25,010a | 1,000 | 19,000 | ,000 | ,568 | 25,010 | ,997 |
|  | 3 | Traza de Pillai | ,760 | 60,023a | 1,000 | 19,000 | ,000 | ,760 | 60,023 | 1,000 |
|  |  | Lambda de Wilks | ,240 | 60,023a | 1,000 | 19,000 | ,000 | ,760 | 60,023 | 1,000 |
|  |  | Traza de Hotelling | 3,159 | 60,023a | 1,000 | 19,000 | ,000 | ,760 | 60,023 | 1,000 |
|  |  | Raíz mayor de Roy | 3,159 | 60,023a | 1,000 | 19,000 | ,000 | ,760 | 60,023 | 1,000 |
|  | 4 | Traza de Pillai | ,823 | 88,235a | 1,000 | 19,000 | ,000 | ,823 | 88,235 | 1,000 |
|  |  | Lambda de Wilks | ,177 | 88,235a | 1,000 | 19,000 | ,000 | ,823 | 88,235 | 1,000 |
|  |  | Traza de Hotelling | 4,644 | 88,235a | 1,000 | 19,000 | ,000 | ,823 | 88,235 | 1,000 |
|  |  | Raíz mayor de Roy | 4,644 | 88,235a | 1,000 | 19,000 | ,000 | ,823 | 88,235 | 1,000 |
|  | 5 | Traza de Pillai | ,762 | 60,782a | 1,000 | 19,000 | ,000 | ,762 | 60,782 | 1,000 |
|  |  | Lambda de Wilks | ,238 | 60,782a | 1,000 | 19,000 | ,000 | ,762 | 60,782 | 1,000 |
|  |  | Traza de Hotelling | 3,199 | 60,782a | 1,000 | 19,000 | ,000 | ,762 | 60,782 | 1,000 |
|  |  | Raíz mayor de Roy | 3,199 | 60,782a | 1,000 | 19,000 | ,000 | ,762 | 60,782 | 1,000 |
|  | 6 | Traza de Pillai | ,813 | 82,776a | 1,000 | 19,000 | ,000 | ,813 | 82,776 | 1,000 |
|  |  | Lambda de Wilks | ,187 | 82,776a | 1,000 | 19,000 | ,000 | ,813 | 82,776 | 1,000 |
|  |  | Traza de Hotelling | 4,357 | 82,776a | 1,000 | 19,000 | ,000 | ,813 | 82,776 | 1,000 |
|  |  | Raíz mayor de Roy | 4,357 | 82,776a | 1,000 | 19,000 | ,000 | ,813 | 82,776 | 1,000 |
| 2 | 1 | Traza de Pillai | ,690 | 42,335a | 1,000 | 19,000 | ,000 | ,690 | 42,335 | 1,000 |
|  |  | Lambda de Wilks | ,310 | 42,335a | 1,000 | 19,000 | ,000 | ,690 | 42,335 | 1,000 |
|  |  | Traza de Hotelling | 2,228 | 42,335a | 1,000 | 19,000 | ,000 | ,690 | 42,335 | 1,000 |
|  |  | Raíz mayor de Roy | 2,228 | 42,335a | 1,000 | 19,000 | ,000 | ,690 | 42,335 | 1,000 |
|  | 2 | Traza de Pillai | ,626 | 31,856a | 1,000 | 19,000 | ,000 | ,626 | 31,856 | 1,000 |
|  |  | Lambda de Wilks | ,374 | 31,856a | 1,000 | 19,000 | ,000 | ,626 | 31,856 | 1,000 |
|  |  | Traza de Hotelling | 1,677 | 31,856a | 1,000 | 19,000 | ,000 | ,626 | 31,856 | 1,000 |
|  |  | Raíz mayor de Roy | 1,677 | 31,856a | 1,000 | 19,000 | ,000 | ,626 | 31,856 | 1,000 |
|  | 3 | Traza de Pillai | ,614 | 30,163a | 1,000 | 19,000 | ,000 | ,614 | 30,163 | ,999 |
|  |  | Lambda de Wilks | ,386 | 30,163a | 1,000 | 19,000 | ,000 | ,614 | 30,163 | ,999 |
|  |  | Traza de Hotelling | 1,588 | 30,163a | 1,000 | 19,000 | ,000 | ,614 | 30,163 | ,999 |
|  |  | Raíz mayor de Roy | 1,588 | 30,163a | 1,000 | 19,000 | ,000 | ,614 | 30,163 | ,999 |
|  | 4 | Traza de Pillai | ,597 | 28,090a | 1,000 | 19,000 | ,000 | ,597 | 28,090 | ,999 |
|  |  | Lambda de Wilks | ,403 | 28,090a | 1,000 | 19,000 | ,000 | ,597 | 28,090 | ,999 |
|  |  | Traza de Hotelling | 1,478 | 28,090a | 1,000 | 19,000 | ,000 | ,597 | 28,090 | ,999 |
|  |  | Raíz mayor de Roy | 1,478 | 28,090a | 1,000 | 19,000 | ,000 | ,597 | 28,090 | ,999 |
|  | 5 | Traza de Pillai | ,289 | 7,735a | 1,000 | 19,000 | ,012 | ,289 | 7,735 | ,751 |
|  |  | Lambda de Wilks | ,711 | 7,735a | 1,000 | 19,000 | ,012 | ,289 | 7,735 | ,751 |
|  |  | Traza de Hotelling | ,407 | 7,735a | 1,000 | 19,000 | ,012 | ,289 | 7,735 | ,751 |
|  |  | Raíz mayor de Roy | ,407 | 7,735a | 1,000 | 19,000 | ,012 | ,289 | 7,735 | ,751 |
|  | 6 | Traza de Pillai | ,257 | 6,564a | 1,000 | 19,000 | ,019 | ,257 | 6,564 | ,681 |
|  |  | Lambda de Wilks | ,743 | 6,564a | 1,000 | 19,000 | ,019 | ,257 | 6,564 | ,681 |
|  |  | Traza de Hotelling | ,345 | 6,564a | 1,000 | 19,000 | ,019 | ,257 | 6,564 | ,681 |
|  |  | Raíz mayor de Roy | ,345 | 6,564a | 1,000 | 19,000 | ,019 | ,257 | 6,564 | ,681 |
| 3 | 1 | Traza de Pillai | ,686 | 41,484a | 1,000 | 19,000 | ,000 | ,686 | 41,484 | 1,000 |
|  |  | Lambda de Wilks | ,314 | 41,484a | 1,000 | 19,000 | ,000 | ,686 | 41,484 | 1,000 |
|  |  | Traza de Hotelling | 2,183 | 41,484a | 1,000 | 19,000 | ,000 | ,686 | 41,484 | 1,000 |
|  |  | Raíz mayor de Roy | 2,183 | 41,484a | 1,000 | 19,000 | ,000 | ,686 | 41,484 | 1,000 |
|  | 2 | Traza de Pillai | ,631 | 32,512a | 1,000 | 19,000 | ,000 | ,631 | 32,512 | 1,000 |
|  |  | Lambda de Wilks | ,369 | 32,512a | 1,000 | 19,000 | ,000 | ,631 | 32,512 | 1,000 |
|  |  | Traza de Hotelling | 1,711 | 32,512a | 1,000 | 19,000 | ,000 | ,631 | 32,512 | 1,000 |
|  |  | Raíz mayor de Roy | 1,711 | 32,512a | 1,000 | 19,000 | ,000 | ,631 | 32,512 | 1,000 |
|  | 3 | Traza de Pillai | ,497 | 18,770a | 1,000 | 19,000 | ,000 | ,497 | 18,770 | ,984 |
|  |  | Lambda de Wilks | ,503 | 18,770a | 1,000 | 19,000 | ,000 | ,497 | 18,770 | ,984 |
|  |  | Traza de Hotelling | ,988 | 18,770a | 1,000 | 19,000 | ,000 | ,497 | 18,770 | ,984 |
|  |  | Raíz mayor de Roy | ,988 | 18,770a | 1,000 | 19,000 | ,000 | ,497 | 18,770 | ,984 |
|  | 4 | Traza de Pillai | ,622 | 31,221a | 1,000 | 19,000 | ,000 | ,622 | 31,221 | 1,000 |
|  |  | Lambda de Wilks | ,378 | 31,221a | 1,000 | 19,000 | ,000 | ,622 | 31,221 | 1,000 |
|  |  | Traza de Hotelling | 1,643 | 31,221a | 1,000 | 19,000 | ,000 | ,622 | 31,221 | 1,000 |
|  |  | Raíz mayor de Roy | 1,643 | 31,221a | 1,000 | 19,000 | ,000 | ,622 | 31,221 | 1,000 |
|  | 5 | Traza de Pillai | ,236 | 5,859a | 1,000 | 19,000 | ,026 | ,236 | 5,859 | ,632 |
|  |  | Lambda de Wilks | ,764 | 5,859a | 1,000 | 19,000 | ,026 | ,236 | 5,859 | ,632 |
|  |  | Traza de Hotelling | ,308 | 5,859a | 1,000 | 19,000 | ,026 | ,236 | 5,859 | ,632 |
|  |  | Raíz mayor de Roy | ,308 | 5,859a | 1,000 | 19,000 | ,026 | ,236 | 5,859 | ,632 |
|  | 6 | Traza de Pillai | ,281 | 7,440a | 1,000 | 19,000 | ,013 | ,281 | 7,440 | ,735 |
|  |  | Lambda de Wilks | ,719 | 7,440a | 1,000 | 19,000 | ,013 | ,281 | 7,440 | ,735 |
|  |  | Traza de Hotelling | ,392 | 7,440a | 1,000 | 19,000 | ,013 | ,281 | 7,440 | ,735 |
|  |  | Raíz mayor de Roy | ,392 | 7,440a | 1,000 | 19,000 | ,013 | ,281 | 7,440 | ,735 |
|  |  |  |  |  |  |  |  |  |  |  |
| --- | --- | --- | --- | --- | --- | --- | --- | --- | --- | --- |
| Cada F prueba los efectos simples multivariantes de act dentro de cada combinación de niveles de los otros efectos mostrados. Estas pruebas se basan en las comparaciones por parejas linealmente independientes entre las medias marginales estimadas. | | | | | | | | | | |
| a. Estadístico exacto | | | | | | | | | | |
| b. Se ha calculado utilizando alpha = ,05 | | | | | | | | | | |
|  |  |  |  |  |  |  |  |  |  |  |

7. Ejerc \* Muscu \* act

Comparaciones por parejasComparaciones por parejas, tabla, Medida, MEASURE\_1, 1 capas, 2 niveles de cabeceras de columna y 4 niveles de cabeceras de fila, tabla con 9 columnas y 79 filas

| |  |  |  | | --- | --- | --- | | Medida: | MEASURE\_1 | MEASURE\_1 | | | | | | | | | |  |
|  |  |  |  |  |  |  |  |  |  |
| --- | --- | --- | --- | --- | --- | --- | --- | --- | --- |
| Muscu | act | (I) Ejerc | (J) Ejerc | Diferencia de medias (I-J) | Desv. Error | Sig.b | 95% de intervalo de confianza para diferenciab | |  |
|  |  |  |  |  |  |  | Límite inferior | Límite superior |  |
| 1 | 1 | 1 | 2 | 10,920 | 8,517 | ,646 | -11,439 | 33,279 |  |
|  |  |  | 3 | -77,510\* | 13,319 | ,000 | -112,473 | -42,547 |  |
|  |  | 2 | 1 | -10,920 | 8,517 | ,646 | -33,279 | 11,439 |  |
|  |  |  | 3 | -88,430\* | 15,460 | ,000 | -129,013 | -47,847 |  |
|  |  | 3 | 1 | 77,510\* | 13,319 | ,000 | 42,547 | 112,473 |  |
|  |  |  | 2 | 88,430\* | 15,460 | ,000 | 47,847 | 129,013 |  |
|  | 2 | 1 | 2 | -11,000\* | 3,316 | ,011 | -19,705 | -2,295 |  |
|  |  |  | 3 | -80,480\* | 12,985 | ,000 | -114,567 | -46,393 |  |
|  |  | 2 | 1 | 11,000\* | 3,316 | ,011 | 2,295 | 19,705 |  |
|  |  |  | 3 | -69,480\* | 12,515 | ,000 | -102,333 | -36,627 |  |
|  |  | 3 | 1 | 80,480\* | 12,985 | ,000 | 46,393 | 114,567 |  |
|  |  |  | 2 | 69,480\* | 12,515 | ,000 | 36,627 | 102,333 |  |
| 2 | 1 | 1 | 2 | -8,480 | 7,989 | ,905 | -29,452 | 12,492 |  |
|  |  |  | 3 | -71,070\* | 11,472 | ,000 | -101,186 | -40,954 |  |
|  |  | 2 | 1 | 8,480 | 7,989 | ,905 | -12,492 | 29,452 |  |
|  |  |  | 3 | -62,590\* | 10,564 | ,000 | -90,320 | -34,860 |  |
|  |  | 3 | 1 | 71,070\* | 11,472 | ,000 | 40,954 | 101,186 |  |
|  |  |  | 2 | 62,590\* | 10,564 | ,000 | 34,860 | 90,320 |  |
|  | 2 | 1 | 2 | -12,030 | 5,227 | ,099 | -25,750 | 1,690 |  |
|  |  |  | 3 | -59,400\* | 9,757 | ,000 | -85,013 | -33,787 |  |
|  |  | 2 | 1 | 12,030 | 5,227 | ,099 | -1,690 | 25,750 |  |
|  |  |  | 3 | -47,370\* | 7,745 | ,000 | -67,701 | -27,039 |  |
|  |  | 3 | 1 | 59,400\* | 9,757 | ,000 | 33,787 | 85,013 |  |
|  |  |  | 2 | 47,370\* | 7,745 | ,000 | 27,039 | 67,701 |  |
| 3 | 1 | 1 | 2 | 3,400 | 5,693 | 1,000 | -11,546 | 18,346 |  |
|  |  |  | 3 | -46,120\* | 8,850 | ,000 | -69,351 | -22,889 |  |
|  |  | 2 | 1 | -3,400 | 5,693 | 1,000 | -18,346 | 11,546 |  |
|  |  |  | 3 | -49,520\* | 7,428 | ,000 | -69,019 | -30,021 |  |
|  |  | 3 | 1 | 46,120\* | 8,850 | ,000 | 22,889 | 69,351 |  |
|  |  |  | 2 | 49,520\* | 7,428 | ,000 | 30,021 | 69,019 |  |
|  | 2 | 1 | 2 | -11,070 | 5,095 | ,128 | -24,444 | 2,304 |  |
|  |  |  | 3 | -49,690\* | 5,847 | ,000 | -65,038 | -34,342 |  |
|  |  | 2 | 1 | 11,070 | 5,095 | ,128 | -2,304 | 24,444 |  |
|  |  |  | 3 | -38,620\* | 7,028 | ,000 | -57,068 | -20,172 |  |
|  |  | 3 | 1 | 49,690\* | 5,847 | ,000 | 34,342 | 65,038 |  |
|  |  |  | 2 | 38,620\* | 7,028 | ,000 | 20,172 | 57,068 |  |
| 4 | 1 | 1 | 2 | -10,840 | 13,186 | 1,000 | -45,456 | 23,776 |  |
|  |  |  | 3 | -117,470\* | 21,699 | ,000 | -174,433 | -60,507 |  |
|  |  | 2 | 1 | 10,840 | 13,186 | 1,000 | -23,776 | 45,456 |  |
|  |  |  | 3 | -106,630\* | 14,151 | ,000 | -143,778 | -69,482 |  |
|  |  | 3 | 1 | 117,470\* | 21,699 | ,000 | 60,507 | 174,433 |  |
|  |  |  | 2 | 106,630\* | 14,151 | ,000 | 69,482 | 143,778 |  |
|  | 2 | 1 | 2 | -53,000\* | 8,039 | ,000 | -74,104 | -31,896 |  |
|  |  |  | 3 | -150,140\* | 18,424 | ,000 | -198,505 | -101,775 |  |
|  |  | 2 | 1 | 53,000\* | 8,039 | ,000 | 31,896 | 74,104 |  |
|  |  |  | 3 | -97,140\* | 15,000 | ,000 | -136,517 | -57,763 |  |
|  |  | 3 | 1 | 150,140\* | 18,424 | ,000 | 101,775 | 198,505 |  |
|  |  |  | 2 | 97,140\* | 15,000 | ,000 | 57,763 | 136,517 |  |
| 5 | 1 | 1 | 2 | 9,890 | 13,540 | 1,000 | -25,654 | 45,434 |  |
|  |  |  | 3 | -71,590\* | 17,137 | ,002 | -116,577 | -26,603 |  |
|  |  | 2 | 1 | -9,890 | 13,540 | 1,000 | -45,434 | 25,654 |  |
|  |  |  | 3 | -81,480\* | 12,652 | ,000 | -114,693 | -48,267 |  |
|  |  | 3 | 1 | 71,590\* | 17,137 | ,002 | 26,603 | 116,577 |  |
|  |  |  | 2 | 81,480\* | 12,652 | ,000 | 48,267 | 114,693 |  |
|  | 2 | 1 | 2 | -51,370\* | 9,814 | ,000 | -77,132 | -25,608 |  |
|  |  |  | 3 | -133,770\* | 18,631 | ,000 | -182,679 | -84,861 |  |
|  |  | 2 | 1 | 51,370\* | 9,814 | ,000 | 25,608 | 77,132 |  |
|  |  |  | 3 | -82,400\* | 16,778 | ,000 | -126,443 | -38,357 |  |
|  |  | 3 | 1 | 133,770\* | 18,631 | ,000 | 84,861 | 182,679 |  |
|  |  |  | 2 | 82,400\* | 16,778 | ,000 | 38,357 | 126,443 |  |
| 6 | 1 | 1 | 2 | 49,610\* | 10,480 | ,000 | 22,099 | 77,121 |  |
|  |  |  | 3 | 19,860 | 11,160 | ,273 | -9,436 | 49,156 |  |
|  |  | 2 | 1 | -49,610\* | 10,480 | ,000 | -77,121 | -22,099 |  |
|  |  |  | 3 | -29,750\* | 9,775 | ,020 | -55,409 | -4,091 |  |
|  |  | 3 | 1 | -19,860 | 11,160 | ,273 | -49,156 | 9,436 |  |
|  |  |  | 2 | 29,750\* | 9,775 | ,020 | 4,091 | 55,409 |  |
|  | 2 | 1 | 2 | -9,960 | 8,937 | ,837 | -33,421 | 13,501 |  |
|  |  |  | 3 | -43,230\* | 9,678 | ,001 | -68,635 | -17,825 |  |
|  |  | 2 | 1 | 9,960 | 8,937 | ,837 | -13,501 | 33,421 |  |
|  |  |  | 3 | -33,270\* | 9,993 | ,011 | -59,502 | -7,038 |  |
|  |  | 3 | 1 | 43,230\* | 9,678 | ,001 | 17,825 | 68,635 |  |
|  |  |  | 2 | 33,270\* | 9,993 | ,011 | 7,038 | 59,502 |  |
|  |  |  |  |  |  |  |  |  |  |
| --- | --- | --- | --- | --- | --- | --- | --- | --- | --- |
| Se basa en medias marginales estimadas | | | | | | | | |  |
| \*. La diferencia de medias es significativa en el nivel ,05. | | | | | | | | |  |
| b. Ajuste para varias comparaciones: Bonferroni. | | | | | | | | |  |
|  |  |  |  |  |  |  |  |  |  |

7. Ejerc \* Muscu \* act

Pruebas multivariantePruebas multivariante, tabla, 1 niveles de cabeceras de columna y 3 niveles de cabeceras de fila, tabla con 11 columnas y 53 filas

|  |  |  |  |  |  |  |  |  |  |  |
| --- | --- | --- | --- | --- | --- | --- | --- | --- | --- | --- |
| Muscu | act | | Valor | F | gl de hipótesis | gl de error | Sig. | Eta parcial al cuadrado | Parámetro sin centralidad | Potencia observadab |
| 1 | 1 | Traza de Pillai | ,657 | 17,202a | 2,000 | 18,000 | ,000 | ,657 | 34,404 | ,999 |
|  |  | Lambda de Wilks | ,343 | 17,202a | 2,000 | 18,000 | ,000 | ,657 | 34,404 | ,999 |
|  |  | Traza de Hotelling | 1,911 | 17,202a | 2,000 | 18,000 | ,000 | ,657 | 34,404 | ,999 |
|  |  | Raíz mayor de Roy | 1,911 | 17,202a | 2,000 | 18,000 | ,000 | ,657 | 34,404 | ,999 |
|  | 2 | Traza de Pillai | ,685 | 19,607a | 2,000 | 18,000 | ,000 | ,685 | 39,215 | 1,000 |
|  |  | Lambda de Wilks | ,315 | 19,607a | 2,000 | 18,000 | ,000 | ,685 | 39,215 | 1,000 |
|  |  | Traza de Hotelling | 2,179 | 19,607a | 2,000 | 18,000 | ,000 | ,685 | 39,215 | 1,000 |
|  |  | Raíz mayor de Roy | 2,179 | 19,607a | 2,000 | 18,000 | ,000 | ,685 | 39,215 | 1,000 |
| 2 | 1 | Traza de Pillai | ,690 | 20,060a | 2,000 | 18,000 | ,000 | ,690 | 40,120 | 1,000 |
|  |  | Lambda de Wilks | ,310 | 20,060a | 2,000 | 18,000 | ,000 | ,690 | 40,120 | 1,000 |
|  |  | Traza de Hotelling | 2,229 | 20,060a | 2,000 | 18,000 | ,000 | ,690 | 40,120 | 1,000 |
|  |  | Raíz mayor de Roy | 2,229 | 20,060a | 2,000 | 18,000 | ,000 | ,690 | 40,120 | 1,000 |
|  | 2 | Traza de Pillai | ,680 | 19,111a | 2,000 | 18,000 | ,000 | ,680 | 38,222 | 1,000 |
|  |  | Lambda de Wilks | ,320 | 19,111a | 2,000 | 18,000 | ,000 | ,680 | 38,222 | 1,000 |
|  |  | Traza de Hotelling | 2,123 | 19,111a | 2,000 | 18,000 | ,000 | ,680 | 38,222 | 1,000 |
|  |  | Raíz mayor de Roy | 2,123 | 19,111a | 2,000 | 18,000 | ,000 | ,680 | 38,222 | 1,000 |
| 3 | 1 | Traza de Pillai | ,701 | 21,061a | 2,000 | 18,000 | ,000 | ,701 | 42,123 | 1,000 |
|  |  | Lambda de Wilks | ,299 | 21,061a | 2,000 | 18,000 | ,000 | ,701 | 42,123 | 1,000 |
|  |  | Traza de Hotelling | 2,340 | 21,061a | 2,000 | 18,000 | ,000 | ,701 | 42,123 | 1,000 |
|  |  | Raíz mayor de Roy | 2,340 | 21,061a | 2,000 | 18,000 | ,000 | ,701 | 42,123 | 1,000 |
|  | 2 | Traza de Pillai | ,793 | 34,413a | 2,000 | 18,000 | ,000 | ,793 | 68,825 | 1,000 |
|  |  | Lambda de Wilks | ,207 | 34,413a | 2,000 | 18,000 | ,000 | ,793 | 68,825 | 1,000 |
|  |  | Traza de Hotelling | 3,824 | 34,413a | 2,000 | 18,000 | ,000 | ,793 | 68,825 | 1,000 |
|  |  | Raíz mayor de Roy | 3,824 | 34,413a | 2,000 | 18,000 | ,000 | ,793 | 68,825 | 1,000 |
| 4 | 1 | Traza de Pillai | ,754 | 27,544a | 2,000 | 18,000 | ,000 | ,754 | 55,089 | 1,000 |
|  |  | Lambda de Wilks | ,246 | 27,544a | 2,000 | 18,000 | ,000 | ,754 | 55,089 | 1,000 |
|  |  | Traza de Hotelling | 3,060 | 27,544a | 2,000 | 18,000 | ,000 | ,754 | 55,089 | 1,000 |
|  |  | Raíz mayor de Roy | 3,060 | 27,544a | 2,000 | 18,000 | ,000 | ,754 | 55,089 | 1,000 |
|  | 2 | Traza de Pillai | ,788 | 33,530a | 2,000 | 18,000 | ,000 | ,788 | 67,059 | 1,000 |
|  |  | Lambda de Wilks | ,212 | 33,530a | 2,000 | 18,000 | ,000 | ,788 | 67,059 | 1,000 |
|  |  | Traza de Hotelling | 3,726 | 33,530a | 2,000 | 18,000 | ,000 | ,788 | 67,059 | 1,000 |
|  |  | Raíz mayor de Roy | 3,726 | 33,530a | 2,000 | 18,000 | ,000 | ,788 | 67,059 | 1,000 |
| 5 | 1 | Traza de Pillai | ,686 | 19,666a | 2,000 | 18,000 | ,000 | ,686 | 39,332 | 1,000 |
|  |  | Lambda de Wilks | ,314 | 19,666a | 2,000 | 18,000 | ,000 | ,686 | 39,332 | 1,000 |
|  |  | Traza de Hotelling | 2,185 | 19,666a | 2,000 | 18,000 | ,000 | ,686 | 39,332 | 1,000 |
|  |  | Raíz mayor de Roy | 2,185 | 19,666a | 2,000 | 18,000 | ,000 | ,686 | 39,332 | 1,000 |
|  | 2 | Traza de Pillai | ,749 | 26,906a | 2,000 | 18,000 | ,000 | ,749 | 53,813 | 1,000 |
|  |  | Lambda de Wilks | ,251 | 26,906a | 2,000 | 18,000 | ,000 | ,749 | 53,813 | 1,000 |
|  |  | Traza de Hotelling | 2,990 | 26,906a | 2,000 | 18,000 | ,000 | ,749 | 53,813 | 1,000 |
|  |  | Raíz mayor de Roy | 2,990 | 26,906a | 2,000 | 18,000 | ,000 | ,749 | 53,813 | 1,000 |
| 6 | 1 | Traza de Pillai | ,559 | 11,391a | 2,000 | 18,000 | ,001 | ,559 | 22,781 | ,981 |
|  |  | Lambda de Wilks | ,441 | 11,391a | 2,000 | 18,000 | ,001 | ,559 | 22,781 | ,981 |
|  |  | Traza de Hotelling | 1,266 | 11,391a | 2,000 | 18,000 | ,001 | ,559 | 22,781 | ,981 |
|  |  | Raíz mayor de Roy | 1,266 | 11,391a | 2,000 | 18,000 | ,001 | ,559 | 22,781 | ,981 |
|  | 2 | Traza de Pillai | ,522 | 9,811a | 2,000 | 18,000 | ,001 | ,522 | 19,623 | ,962 |
|  |  | Lambda de Wilks | ,478 | 9,811a | 2,000 | 18,000 | ,001 | ,522 | 19,623 | ,962 |
|  |  | Traza de Hotelling | 1,090 | 9,811a | 2,000 | 18,000 | ,001 | ,522 | 19,623 | ,962 |
|  |  | Raíz mayor de Roy | 1,090 | 9,811a | 2,000 | 18,000 | ,001 | ,522 | 19,623 | ,962 |
|  |  |  |  |  |  |  |  |  |  |  |
| --- | --- | --- | --- | --- | --- | --- | --- | --- | --- | --- |
| Cada F prueba los efectos simples multivariantes de Ejerc dentro de cada combinación de niveles de los otros efectos mostrados. Estas pruebas se basan en las comparaciones por parejas linealmente independientes entre las medias marginales estimadas. | | | | | | | | | | |
| a. Estadístico exacto | | | | | | | | | | |
| b. Se ha calculado utilizando alpha = ,05 | | | | | | | | | | |
|  |  |  |  |  |  |  |  |  |  |  |

7. Ejerc \* Muscu \* act

Comparaciones por parejasComparaciones por parejas, tabla, Medida, MEASURE\_1, 1 capas, 2 niveles de cabeceras de columna y 4 niveles de cabeceras de fila, tabla con 9 columnas y 187 filas

| |  |  |  | | --- | --- | --- | | Medida: | MEASURE\_1 | MEASURE\_1 | | | | | | | | | |  |
|  |  |  |  |  |  |  |  |  |  |
| --- | --- | --- | --- | --- | --- | --- | --- | --- | --- |
| Ejerc | act | (I) Muscu | (J) Muscu | Diferencia de medias (I-J) | Desv. Error | Sig.b | 95% de intervalo de confianza para diferenciab | |  |
|  |  |  |  |  |  |  | Límite inferior | Límite superior |  |
| 1 | 1 | 1 | 2 | 19,560 | 10,024 | ,989 | -14,061 | 53,181 |  |
|  |  |  | 3 | 25,000 | 9,842 | ,300 | -8,009 | 58,009 |  |
|  |  |  | 4 | -80,170\* | 13,495 | ,000 | -125,432 | -34,908 |  |
|  |  |  | 5 | -99,540\* | 12,802 | ,000 | -142,477 | -56,603 |  |
|  |  |  | 6 | -80,830\* | 11,624 | ,000 | -119,816 | -41,844 |  |
|  |  | 2 | 1 | -19,560 | 10,024 | ,989 | -53,181 | 14,061 |  |
|  |  |  | 3 | 5,440 | 11,086 | 1,000 | -31,742 | 42,622 |  |
|  |  |  | 4 | -99,730\* | 12,676 | ,000 | -142,245 | -57,215 |  |
|  |  |  | 5 | -119,100\* | 17,105 | ,000 | -176,472 | -61,728 |  |
|  |  |  | 6 | -100,390\* | 15,369 | ,000 | -151,939 | -48,841 |  |
|  |  | 3 | 1 | -25,000 | 9,842 | ,300 | -58,009 | 8,009 |  |
|  |  |  | 2 | -5,440 | 11,086 | 1,000 | -42,622 | 31,742 |  |
|  |  |  | 4 | -105,170\* | 14,813 | ,000 | -154,854 | -55,486 |  |
|  |  |  | 5 | -124,540\* | 12,986 | ,000 | -168,095 | -80,985 |  |
|  |  |  | 6 | -105,830\* | 10,892 | ,000 | -142,361 | -69,299 |  |
|  |  | 4 | 1 | 80,170\* | 13,495 | ,000 | 34,908 | 125,432 |  |
|  |  |  | 2 | 99,730\* | 12,676 | ,000 | 57,215 | 142,245 |  |
|  |  |  | 3 | 105,170\* | 14,813 | ,000 | 55,486 | 154,854 |  |
|  |  |  | 5 | -19,370 | 16,198 | 1,000 | -73,699 | 34,959 |  |
|  |  |  | 6 | -,660 | 18,005 | 1,000 | -61,049 | 59,729 |  |
|  |  | 5 | 1 | 99,540\* | 12,802 | ,000 | 56,603 | 142,477 |  |
|  |  |  | 2 | 119,100\* | 17,105 | ,000 | 61,728 | 176,472 |  |
|  |  |  | 3 | 124,540\* | 12,986 | ,000 | 80,985 | 168,095 |  |
|  |  |  | 4 | 19,370 | 16,198 | 1,000 | -34,959 | 73,699 |  |
|  |  |  | 6 | 18,710 | 15,062 | 1,000 | -31,810 | 69,230 |  |
|  |  | 6 | 1 | 80,830\* | 11,624 | ,000 | 41,844 | 119,816 |  |
|  |  |  | 2 | 100,390\* | 15,369 | ,000 | 48,841 | 151,939 |  |
|  |  |  | 3 | 105,830\* | 10,892 | ,000 | 69,299 | 142,361 |  |
|  |  |  | 4 | ,660 | 18,005 | 1,000 | -59,729 | 61,049 |  |
|  |  |  | 5 | -18,710 | 15,062 | 1,000 | -69,230 | 31,810 |  |
|  | 2 | 1 | 2 | 17,600 | 7,878 | ,565 | -8,824 | 44,024 |  |
|  |  |  | 3 | 8,740 | 5,417 | 1,000 | -9,427 | 26,907 |  |
|  |  |  | 4 | -51,140\* | 7,095 | ,000 | -74,936 | -27,344 |  |
|  |  |  | 5 | -63,540\* | 6,952 | ,000 | -86,856 | -40,224 |  |
|  |  |  | 6 | -57,680\* | 7,982 | ,000 | -84,451 | -30,909 |  |
|  |  | 2 | 1 | -17,600 | 7,878 | ,565 | -44,024 | 8,824 |  |
|  |  |  | 3 | -8,860 | 5,488 | 1,000 | -27,267 | 9,547 |  |
|  |  |  | 4 | -68,740\* | 8,522 | ,000 | -97,323 | -40,157 |  |
|  |  |  | 5 | -81,140\* | 10,207 | ,000 | -115,375 | -46,905 |  |
|  |  |  | 6 | -75,280\* | 9,748 | ,000 | -107,975 | -42,585 |  |
|  |  | 3 | 1 | -8,740 | 5,417 | 1,000 | -26,907 | 9,427 |  |
|  |  |  | 2 | 8,860 | 5,488 | 1,000 | -9,547 | 27,267 |  |
|  |  |  | 4 | -59,880\* | 7,998 | ,000 | -86,707 | -33,053 |  |
|  |  |  | 5 | -72,280\* | 7,050 | ,000 | -95,925 | -48,635 |  |
|  |  |  | 6 | -66,420\* | 6,633 | ,000 | -88,666 | -44,174 |  |
|  |  | 4 | 1 | 51,140\* | 7,095 | ,000 | 27,344 | 74,936 |  |
|  |  |  | 2 | 68,740\* | 8,522 | ,000 | 40,157 | 97,323 |  |
|  |  |  | 3 | 59,880\* | 7,998 | ,000 | 33,053 | 86,707 |  |
|  |  |  | 5 | -12,400 | 8,824 | 1,000 | -41,997 | 17,197 |  |
|  |  |  | 6 | -6,540 | 10,596 | 1,000 | -42,080 | 29,000 |  |
|  |  | 5 | 1 | 63,540\* | 6,952 | ,000 | 40,224 | 86,856 |  |
|  |  |  | 2 | 81,140\* | 10,207 | ,000 | 46,905 | 115,375 |  |
|  |  |  | 3 | 72,280\* | 7,050 | ,000 | 48,635 | 95,925 |  |
|  |  |  | 4 | 12,400 | 8,824 | 1,000 | -17,197 | 41,997 |  |
|  |  |  | 6 | 5,860 | 9,090 | 1,000 | -24,627 | 36,347 |  |
|  |  | 6 | 1 | 57,680\* | 7,982 | ,000 | 30,909 | 84,451 |  |
|  |  |  | 2 | 75,280\* | 9,748 | ,000 | 42,585 | 107,975 |  |
|  |  |  | 3 | 66,420\* | 6,633 | ,000 | 44,174 | 88,666 |  |
|  |  |  | 4 | 6,540 | 10,596 | 1,000 | -29,000 | 42,080 |  |
|  |  |  | 5 | -5,860 | 9,090 | 1,000 | -36,347 | 24,627 |  |
| 2 | 1 | 1 | 2 | ,160 | 10,325 | 1,000 | -34,470 | 34,790 |  |
|  |  |  | 3 | 17,480 | 6,682 | ,255 | -4,933 | 39,893 |  |
|  |  |  | 4 | -101,930\* | 13,495 | ,000 | -147,194 | -56,666 |  |
|  |  |  | 5 | -100,570\* | 15,939 | ,000 | -154,030 | -47,110 |  |
|  |  |  | 6 | -42,140\* | 8,372 | ,001 | -70,219 | -14,061 |  |
|  |  | 2 | 1 | -,160 | 10,325 | 1,000 | -34,790 | 34,470 |  |
|  |  |  | 3 | 17,320 | 12,425 | 1,000 | -24,353 | 58,993 |  |
|  |  |  | 4 | -102,090\* | 12,535 | ,000 | -144,133 | -60,047 |  |
|  |  |  | 5 | -100,730\* | 19,770 | ,001 | -167,040 | -34,420 |  |
|  |  |  | 6 | -42,300 | 13,639 | ,088 | -88,045 | 3,445 |  |
|  |  | 3 | 1 | -17,480 | 6,682 | ,255 | -39,893 | 4,933 |  |
|  |  |  | 2 | -17,320 | 12,425 | 1,000 | -58,993 | 24,353 |  |
|  |  |  | 4 | -119,410\* | 15,181 | ,000 | -170,327 | -68,493 |  |
|  |  |  | 5 | -118,050\* | 14,376 | ,000 | -166,267 | -69,833 |  |
|  |  |  | 6 | -59,620\* | 7,517 | ,000 | -84,833 | -34,407 |  |
|  |  | 4 | 1 | 101,930\* | 13,495 | ,000 | 56,666 | 147,194 |  |
|  |  |  | 2 | 102,090\* | 12,535 | ,000 | 60,047 | 144,133 |  |
|  |  |  | 3 | 119,410\* | 15,181 | ,000 | 68,493 | 170,327 |  |
|  |  |  | 5 | 1,360 | 20,540 | 1,000 | -67,532 | 70,252 |  |
|  |  |  | 6 | 59,790\* | 16,436 | ,026 | 4,662 | 114,918 |  |
|  |  | 5 | 1 | 100,570\* | 15,939 | ,000 | 47,110 | 154,030 |  |
|  |  |  | 2 | 100,730\* | 19,770 | ,001 | 34,420 | 167,040 |  |
|  |  |  | 3 | 118,050\* | 14,376 | ,000 | 69,833 | 166,267 |  |
|  |  |  | 4 | -1,360 | 20,540 | 1,000 | -70,252 | 67,532 |  |
|  |  |  | 6 | 58,430\* | 15,062 | ,015 | 7,913 | 108,947 |  |
|  |  | 6 | 1 | 42,140\* | 8,372 | ,001 | 14,061 | 70,219 |  |
|  |  |  | 2 | 42,300 | 13,639 | ,088 | -3,445 | 88,045 |  |
|  |  |  | 3 | 59,620\* | 7,517 | ,000 | 34,407 | 84,833 |  |
|  |  |  | 4 | -59,790\* | 16,436 | ,026 | -114,918 | -4,662 |  |
|  |  |  | 5 | -58,430\* | 15,062 | ,015 | -108,947 | -7,913 |  |
|  | 2 | 1 | 2 | 16,570 | 5,899 | ,168 | -3,216 | 36,356 |  |
|  |  |  | 3 | 8,670 | 6,718 | 1,000 | -13,864 | 31,204 |  |
|  |  |  | 4 | -93,140\* | 10,549 | ,000 | -128,523 | -57,757 |  |
|  |  |  | 5 | -103,910\* | 14,356 | ,000 | -152,061 | -55,759 |  |
|  |  |  | 6 | -56,640\* | 7,774 | ,000 | -82,713 | -30,567 |  |
|  |  | 2 | 1 | -16,570 | 5,899 | ,168 | -36,356 | 3,216 |  |
|  |  |  | 3 | -7,900 | 7,471 | 1,000 | -32,959 | 17,159 |  |
|  |  |  | 4 | -109,710\* | 10,130 | ,000 | -143,685 | -75,735 |  |
|  |  |  | 5 | -120,480\* | 14,911 | ,000 | -170,491 | -70,469 |  |
|  |  |  | 6 | -73,210\* | 8,735 | ,000 | -102,508 | -43,912 |  |
|  |  | 3 | 1 | -8,670 | 6,718 | 1,000 | -31,204 | 13,864 |  |
|  |  |  | 2 | 7,900 | 7,471 | 1,000 | -17,159 | 32,959 |  |
|  |  |  | 4 | -101,810\* | 12,031 | ,000 | -142,162 | -61,458 |  |
|  |  |  | 5 | -112,580\* | 11,496 | ,000 | -151,137 | -74,023 |  |
|  |  |  | 6 | -65,310\* | 8,761 | ,000 | -94,694 | -35,926 |  |
|  |  | 4 | 1 | 93,140\* | 10,549 | ,000 | 57,757 | 128,523 |  |
|  |  |  | 2 | 109,710\* | 10,130 | ,000 | 75,735 | 143,685 |  |
|  |  |  | 3 | 101,810\* | 12,031 | ,000 | 61,458 | 142,162 |  |
|  |  |  | 5 | -10,770 | 17,206 | 1,000 | -68,481 | 46,941 |  |
|  |  |  | 6 | 36,500 | 13,559 | ,217 | -8,977 | 81,977 |  |
|  |  | 5 | 1 | 103,910\* | 14,356 | ,000 | 55,759 | 152,061 |  |
|  |  |  | 2 | 120,480\* | 14,911 | ,000 | 70,469 | 170,491 |  |
|  |  |  | 3 | 112,580\* | 11,496 | ,000 | 74,023 | 151,137 |  |
|  |  |  | 4 | 10,770 | 17,206 | 1,000 | -46,941 | 68,481 |  |
|  |  |  | 6 | 47,270\* | 13,391 | ,034 | 2,355 | 92,185 |  |
|  |  | 6 | 1 | 56,640\* | 7,774 | ,000 | 30,567 | 82,713 |  |
|  |  |  | 2 | 73,210\* | 8,735 | ,000 | 43,912 | 102,508 |  |
|  |  |  | 3 | 65,310\* | 8,761 | ,000 | 35,926 | 94,694 |  |
|  |  |  | 4 | -36,500 | 13,559 | ,217 | -81,977 | 8,977 |  |
|  |  |  | 5 | -47,270\* | 13,391 | ,034 | -92,185 | -2,355 |  |
| 3 | 1 | 1 | 2 | 26,000 | 18,940 | 1,000 | -37,526 | 89,526 |  |
|  |  |  | 3 | 56,390 | 18,835 | ,112 | -6,782 | 119,562 |  |
|  |  |  | 4 | -120,130\* | 27,222 | ,004 | -211,434 | -28,826 |  |
|  |  |  | 5 | -93,620\* | 21,475 | ,005 | -165,648 | -21,592 |  |
|  |  |  | 6 | 16,540 | 18,241 | 1,000 | -44,642 | 77,722 |  |
|  |  | 2 | 1 | -26,000 | 18,940 | 1,000 | -89,526 | 37,526 |  |
|  |  |  | 3 | 30,390 | 20,085 | 1,000 | -36,975 | 97,755 |  |
|  |  |  | 4 | -146,130\* | 22,240 | ,000 | -220,722 | -71,538 |  |
|  |  |  | 5 | -119,620\* | 26,318 | ,003 | -207,893 | -31,347 |  |
|  |  |  | 6 | -9,460 | 21,780 | 1,000 | -82,510 | 63,590 |  |
|  |  | 3 | 1 | -56,390 | 18,835 | ,112 | -119,562 | 6,782 |  |
|  |  |  | 2 | -30,390 | 20,085 | 1,000 | -97,755 | 36,975 |  |
|  |  |  | 4 | -176,520\* | 25,347 | ,000 | -261,536 | -91,504 |  |
|  |  |  | 5 | -150,010\* | 24,097 | ,000 | -230,834 | -69,186 |  |
|  |  |  | 6 | -39,850 | 15,680 | ,299 | -92,442 | 12,742 |  |
|  |  | 4 | 1 | 120,130\* | 27,222 | ,004 | 28,826 | 211,434 |  |
|  |  |  | 2 | 146,130\* | 22,240 | ,000 | 71,538 | 220,722 |  |
|  |  |  | 3 | 176,520\* | 25,347 | ,000 | 91,504 | 261,536 |  |
|  |  |  | 5 | 26,510 | 31,437 | 1,000 | -78,931 | 131,951 |  |
|  |  |  | 6 | 136,670\* | 27,483 | ,001 | 44,490 | 228,850 |  |
|  |  | 5 | 1 | 93,620\* | 21,475 | ,005 | 21,592 | 165,648 |  |
|  |  |  | 2 | 119,620\* | 26,318 | ,003 | 31,347 | 207,893 |  |
|  |  |  | 3 | 150,010\* | 24,097 | ,000 | 69,186 | 230,834 |  |
|  |  |  | 4 | -26,510 | 31,437 | 1,000 | -131,951 | 78,931 |  |
|  |  |  | 6 | 110,160\* | 22,007 | ,001 | 36,349 | 183,971 |  |
|  |  | 6 | 1 | -16,540 | 18,241 | 1,000 | -77,722 | 44,642 |  |
|  |  |  | 2 | 9,460 | 21,780 | 1,000 | -63,590 | 82,510 |  |
|  |  |  | 3 | 39,850 | 15,680 | ,299 | -12,742 | 92,442 |  |
|  |  |  | 4 | -136,670\* | 27,483 | ,001 | -228,850 | -44,490 |  |
|  |  |  | 5 | -110,160\* | 22,007 | ,001 | -183,971 | -36,349 |  |
|  | 2 | 1 | 2 | 38,680 | 12,110 | ,072 | -1,939 | 79,299 |  |
|  |  |  | 3 | 39,530 | 14,102 | ,170 | -7,769 | 86,829 |  |
|  |  |  | 4 | -120,800\* | 23,330 | ,001 | -199,051 | -42,549 |  |
|  |  |  | 5 | -116,830\* | 20,022 | ,000 | -183,986 | -49,674 |  |
|  |  |  | 6 | -20,430 | 15,067 | 1,000 | -70,967 | 30,107 |  |
|  |  | 2 | 1 | -38,680 | 12,110 | ,072 | -79,299 | 1,939 |  |
|  |  |  | 3 | ,850 | 10,982 | 1,000 | -35,985 | 37,685 |  |
|  |  |  | 4 | -159,480\* | 19,425 | ,000 | -224,632 | -94,328 |  |
|  |  |  | 5 | -155,510\* | 21,841 | ,000 | -228,765 | -82,255 |  |
|  |  |  | 6 | -59,110\* | 15,371 | ,016 | -110,665 | -7,555 |  |
|  |  | 3 | 1 | -39,530 | 14,102 | ,170 | -86,829 | 7,769 |  |
|  |  |  | 2 | -,850 | 10,982 | 1,000 | -37,685 | 35,985 |  |
|  |  |  | 4 | -160,330\* | 24,353 | ,000 | -242,010 | -78,650 |  |
|  |  |  | 5 | -156,360\* | 23,311 | ,000 | -234,547 | -78,173 |  |
|  |  |  | 6 | -59,960\* | 11,674 | ,001 | -99,113 | -20,807 |  |
|  |  | 4 | 1 | 120,800\* | 23,330 | ,001 | 42,549 | 199,051 |  |
|  |  |  | 2 | 159,480\* | 19,425 | ,000 | 94,328 | 224,632 |  |
|  |  |  | 3 | 160,330\* | 24,353 | ,000 | 78,650 | 242,010 |  |
|  |  |  | 5 | 3,970 | 26,707 | 1,000 | -85,607 | 93,547 |  |
|  |  |  | 6 | 100,370\* | 25,306 | ,012 | 15,492 | 185,248 |  |
|  |  | 5 | 1 | 116,830\* | 20,022 | ,000 | 49,674 | 183,986 |  |
|  |  |  | 2 | 155,510\* | 21,841 | ,000 | 82,255 | 228,765 |  |
|  |  |  | 3 | 156,360\* | 23,311 | ,000 | 78,173 | 234,547 |  |
|  |  |  | 4 | -3,970 | 26,707 | 1,000 | -93,547 | 85,607 |  |
|  |  |  | 6 | 96,400\* | 21,206 | ,003 | 25,274 | 167,526 |  |
|  |  | 6 | 1 | 20,430 | 15,067 | 1,000 | -30,107 | 70,967 |  |
|  |  |  | 2 | 59,110\* | 15,371 | ,016 | 7,555 | 110,665 |  |
|  |  |  | 3 | 59,960\* | 11,674 | ,001 | 20,807 | 99,113 |  |
|  |  |  | 4 | -100,370\* | 25,306 | ,012 | -185,248 | -15,492 |  |
|  |  |  | 5 | -96,400\* | 21,206 | ,003 | -167,526 | -25,274 |  |
|  |  |  |  |  |  |  |  |  |  |
| --- | --- | --- | --- | --- | --- | --- | --- | --- | --- |
| Se basa en medias marginales estimadas | | | | | | | | |  |
| \*. La diferencia de medias es significativa en el nivel ,05. | | | | | | | | |  |
| b. Ajuste para varias comparaciones: Bonferroni. | | | | | | | | |  |
|  |  |  |  |  |  |  |  |  |  |

7. Ejerc \* Muscu \* act

Pruebas multivariantePruebas multivariante, tabla, 1 niveles de cabeceras de columna y 3 niveles de cabeceras de fila, tabla con 11 columnas y 13 filas

|  |  |  |  |  |  |  |  |  |  |  |
| --- | --- | --- | --- | --- | --- | --- | --- | --- | --- | --- |
| Ejerc | act | | Valor | F | gl de hipótesis | gl de error | Sig. | Eta parcial al cuadrado | Parámetro sin centralidad | Potencia observadab |
| 1 | 1 | Traza de Pillai | ,919 | 34,221a | 5,000 | 15,000 | ,000 | ,919 | 171,104 | 1,000 |
|  |  | Lambda de Wilks | ,081 | 34,221a | 5,000 | 15,000 | ,000 | ,919 | 171,104 | 1,000 |
|  |  | Traza de Hotelling | 11,407 | 34,221a | 5,000 | 15,000 | ,000 | ,919 | 171,104 | 1,000 |
|  |  | Raíz mayor de Roy | 11,407 | 34,221a | 5,000 | 15,000 | ,000 | ,919 | 171,104 | 1,000 |
|  | 2 | Traza de Pillai | ,931 | 40,730a | 5,000 | 15,000 | ,000 | ,931 | 203,652 | 1,000 |
|  |  | Lambda de Wilks | ,069 | 40,730a | 5,000 | 15,000 | ,000 | ,931 | 203,652 | 1,000 |
|  |  | Traza de Hotelling | 13,577 | 40,730a | 5,000 | 15,000 | ,000 | ,931 | 203,652 | 1,000 |
|  |  | Raíz mayor de Roy | 13,577 | 40,730a | 5,000 | 15,000 | ,000 | ,931 | 203,652 | 1,000 |
|  |  |  |  |  |  |  |  |  |  |  |
| --- | --- | --- | --- | --- | --- | --- | --- | --- | --- | --- |
| Cada F prueba los efectos simples multivariantes de Muscu dentro de cada combinación de niveles de los otros efectos mostrados. Estas pruebas se basan en las comparaciones por parejas linealmente independientes entre las medias marginales estimadas. | | | | | | | | | | |
| a. Estadístico exacto | | | | | | | | | | |
| b. Se ha calculado utilizando alpha = ,05 | | | | | | | | | | |
|  |  |  |  |  |  |  |  |  |  |  |

IBM SPSS Informe web

X

Acerca de

|  |
| --- |
| Creado con: IBM SPSS Statistics 26 |
| Fecha de creación: feb 24, 2020 |
| Versión del documento: OriginalCopia guardada |
| Fecha guardada:  feb 24, 2020 |

Controles de navegación

|  |
| --- |
| Contenido - Abre y cierra la lista de gráficos y tablas en el informe web |
| Siguiente & Anterior - Muestra la tabla o gráfico siguiente o anterior en el informe web |
| Ayuda - Abre la ayuda |

Botones de la barra de herramientas

|  |  |
| --- | --- |
|  | Deshacer - Deshace el último cambio en un documento. |
|  | Editar - Abre la herramienta de Editor para tablas y gráficos. Ciertas opciones de edición sólo están disponibles cuando se está conectado a un servidor de Internet. |
|  | Guardar - Crea una nueva copia del informe web con los cambios guardados. |
|  | Imprimir - Muestra el objeto actual desde la Vista de objeto y todos los objetos de la Vista de página. |
|  | Vista de página - Cambia el informe web para que se muestren todas las tablas y gráficos en una sola página. |
|  | Vista de objeto - Cambia el informe web para que las tablas y gráficos de muestren de uno en uno. |

Conexión con un servidor

:   El estado de la conexión del informe web a un servidor de Internet se muestra en la esquina superior derecha del informe web.
:   No se requiere una conexión a Internet para abrir un informe web. Con la copia guardada del informe web puede ver todos los gráficos y tablas y tener cierta capacidad de edición, aun sin estar conectado a Internet.
:   Si el informe web está conectado a un servidor de Internet, se dispondrá de mejores capacidades de edición para tablas y gráficos.

- Si el autor ha especificado un servidor de Internet al crear el informe web, el informe web intentará conectarse al servidor automáticamente al abrirlo.
- Si el informe web no se conecta a un servidor, pulse en el mensaje de estado del servidor para abrir las herramientas para reintentar la conexión, pruebe con otro servidor o especifique una nueva dirección de servidor.
- Para obtener más información sobre cómo añadir los controles mejorados al servidor de Internet, vaya a https://developer.ibm.com/predictiveanalytics.
- Si especifica una nueva conexión de servidor, el formato preferido es http://xxx.xxx.xxx.xxx:xxxx.

Edición de tablas

|  |  |
| --- | --- |
| Parte de esta funcionalidad sólo está disponible cuando se conecta a un servidor de Internet. | |
|  | Crear un gráfico - Crea un gráfico a partir de las celdas seleccionadas en la tabla. |
|  | Girar y ordenar - Transponer, ordenar y girar la tabla. |
|  | Color de fondo - Color de fondo de las celdas seleccionadas. |
|  | Color y estilo del texto - Color, estilo y tamaño de la letra. |
|  | Formato de numeración - Color, estilo y tamaño de la letra. |

Edición de gráficos

|  |  |
| --- | --- |
| Todo esta funcionalidad sólo está disponible cuando se conecta a un servidor de Internet. | |
|  | Tamaño del gráfico - Cambia la altura y la anchura del gráfico |
|  | Color de fondo - Color de fondo del objeto seleccionado. |
|  | Estilo de línea y borde - Color y grosor de la línea o del borde. |
|  | Color y estilo del texto - Color, estilo y tamaño de la letra. |
|  | Formato de numeración - Color, estilo y tamaño de la letra. |
|  | Propiedades de los ejes - Cambia y escala los títulos y las marcas de los ejes. |
