# Supplementary material for: Electromyographic activity in the gluteus medius, gluteus maximus, biceps femoris, vastus lateralis, vastus medialis and rectus femoris during the Monopodal Squat, Forward Lunge and Lateral Step-Up exercises
Source: PLoS One. 2020 Apr 1;15(4):e0230841. doi: 10.1371/journal.pone.0230841 (PMC7112217; doi:10.1371/journal.pone.0230841)
Supplement: S2 File — (HTM) [file pone.0230841.s002.htm]

xml version="1.0" encoding="UTF-8"?


ANOVA\_3\_6


|  |  |  |
| --- | --- | --- |
| IBM SPSS Informe web - ResultadoANOVArep3x6.spv     ---   Contenido  Anterior  Siguiente  Ayuda |  | Controles inhabilitados por el sistema     --- |

- Registro

  - Registro
- Modelo lineal general

  - Notas
  - Advertencias
  - Factores intra-sujetos
  - Estadísticos descriptivos
  - Pruebas multivariante
  - Prueba de esfericidad de Mauchly
  - Pruebas de efectos intra-sujetos
  - Pruebas de contrastes intra-sujetos
  - Pruebas de efectos inter-sujetos
  - Medias marginales estimadas

    - 1. Ejerc

      - Estimaciones
      - Comparaciones por parejas
      - Pruebas multivariante
    - 2. Muscu

      - Estimaciones
      - Comparaciones por parejas
      - Pruebas multivariante
    - 3. Ejerc \* Muscu

      - Estimaciones
      - Comparaciones por parejas
      - Pruebas multivariante
      - 3. Ejerc \* Muscu

        - Comparaciones por parejas
        - Pruebas multivariante
  - Gráficos de perfil

    - Ejerc \* Muscu
    - Muscu \* Ejerc

- Suprimir

Registro  
Registro - Registro - febrero 24, 2020

DATASET ACTIVATE ConjuntoDatos1.  
GLM STEP\_UP\_PORC\_MVIC\_GMED STEP\_UP\_PORC\_MVIC\_GMAX STEP\_UP\_PORC\_MVIC\_BF STEP\_UP\_PORC\_MVIC\_VL  
    STEP\_UP\_PORC\_MVIC\_VM STEP\_UP\_PORC\_MVIC\_RF LUNGE\_PORC\_MVIC\_GMED LUNGE\_PORC\_MVIC\_GMAX  
    LUNGE\_PORC\_MVIC\_BF LUNGE\_PORC\_MVIC\_VL LUNGE\_PORC\_MVIC\_VM LUNGE\_PORC\_MVIC\_RF SQ\_PORC\_MVIC\_GMED  
    SQ\_PORC\_MVIC\_GMAX SQ\_PORC\_MVIC\_BF SQ\_PORC\_MVIC\_VL SQ\_PORC\_MVIC\_VM SQ\_PORC\_MVIC\_RF  
  /WSFACTOR=Ejerc 3 Polynomial Muscu 6 Polynomial  
  /METHOD=SSTYPE(3)  
  /PLOT=PROFILE(Ejerc\*Muscu) TYPE=BAR ERRORBAR=CI MEANREFERENCE=NO  
  /EMMEANS=TABLES(Ejerc) COMPARE ADJ(BONFERRONI)  
  /EMMEANS=TABLES(Muscu) COMPARE ADJ(BONFERRONI)  
  /EMMEANS=TABLES(Ejerc\*Muscu) COMPARE(Muscu)ADJ(BONFERRONI)  
  /PRINT=DESCRIPTIVE ETASQ OPOWER HOMOGENEITY  
  /CRITERIA=ALPHA(.05)  
  /WSDESIGN=Ejerc Muscu Ejerc\*Muscu.

Modelo lineal general

NotasNotas, tabla, 0 niveles de cabeceras de columna y 2 niveles de cabeceras de fila, tabla con 3 columnas y 14 filas

|  |  |  |
| --- | --- | --- |
| Salida creada | | 20-FEB-2020 18:48:07 |
| Comentarios | |  |
| Entrada | Datos | C:\Users\User\Desktop\BASE\_DATOS\_TFM\_ISA\_PlosOne.sav |
|  | Conjunto de datos activo | ConjuntoDatos1 |
|  | Filtro | <ninguno> |
|  | Ponderación | <ninguno> |
|  | Segmentar archivo | <ninguno> |
|  | N de filas en el archivo de datos de trabajo | 20 |
| Manejo de valores perdidos | Definición de perdidos | Los valores perdidos definidos por el usuario se tratan como perdidos. |
|  | Casos utilizados | Las estadísticas se basan en todos los casos con datos válidos para todas las variables del modelo. |
| Sintaxis | | GLM STEP\_UP\_PORC\_MVIC\_GMED STEP\_UP\_PORC\_MVIC\_GMAX STEP\_UP\_PORC\_MVIC\_BF STEP\_UP\_PORC\_MVIC\_VL  STEP\_UP\_PORC\_MVIC\_VM STEP\_UP\_PORC\_MVIC\_RF LUNGE\_PORC\_MVIC\_GMED LUNGE\_PORC\_MVIC\_GMAX  LUNGE\_PORC\_MVIC\_BF LUNGE\_PORC\_MVIC\_VL LUNGE\_PORC\_MVIC\_VM LUNGE\_PORC\_MVIC\_RF SQ\_PORC\_MVIC\_GMED  SQ\_PORC\_MVIC\_GMAX SQ\_PORC\_MVIC\_BF SQ\_PORC\_MVIC\_VL SQ\_PORC\_MVIC\_VM SQ\_PORC\_MVIC\_RF  /WSFACTOR=Ejerc 3 Polynomial Muscu 6 Polynomial  /METHOD=SSTYPE(3)  /PLOT=PROFILE(Ejerc\*Muscu) TYPE=BAR ERRORBAR=CI MEANREFERENCE=NO  /EMMEANS=TABLES(Ejerc) COMPARE ADJ(BONFERRONI)  /EMMEANS=TABLES(Muscu) COMPARE ADJ(BONFERRONI)  /EMMEANS=TABLES(Ejerc\*Muscu) COMPARE(Muscu)ADJ(BONFERRONI)  /PRINT=DESCRIPTIVE ETASQ OPOWER HOMOGENEITY  /CRITERIA=ALPHA(.05)  /WSDESIGN=Ejerc Muscu Ejerc\*Muscu. |
| Recursos | Tiempo de procesador | 00:00:00,47 |
|  | Tiempo transcurrido | 00:00:00,18 |
|  |  |  |

Modelo lineal general

AdvertenciasAdvertencias, tabla, 0 niveles de cabeceras de columna y 0 niveles de cabeceras de fila, tabla con 1 columnas y 2 filas

|  |
| --- |
| La especificación HOMOGENEITY en el subcomando PRINT se ignorará porque no hay factores inter-sujetos. |
|  |

Modelo lineal general

Factores intra-sujetosFactores intra-sujetos, tabla, Medida, MEASURE\_1, 1 capas, 1 niveles de cabeceras de columna y 2 niveles de cabeceras de fila, tabla con 3 columnas y 21 filas

| |  |  |  | | --- | --- | --- | | Medida: | MEASURE\_1 | MEASURE\_1 | | | |
|  |  |  |
| --- | --- | --- |
| Ejerc | Muscu | Variable dependiente |
| 1 | 1 | STEP\_UP\_PORC\_MVIC\_GMED |
|  | 2 | STEP\_UP\_PORC\_MVIC\_GMAX |
|  | 3 | STEP\_UP\_PORC\_MVIC\_BF |
|  | 4 | STEP\_UP\_PORC\_MVIC\_VL |
|  | 5 | STEP\_UP\_PORC\_MVIC\_VM |
|  | 6 | STEP\_UP\_PORC\_MVIC\_RF |
| 2 | 1 | LUNGE\_PORC\_MVIC\_GMED |
|  | 2 | LUNGE\_PORC\_MVIC\_GMAX |
|  | 3 | LUNGE\_PORC\_MVIC\_BF |
|  | 4 | LUNGE\_PORC\_MVIC\_VL |
|  | 5 | LUNGE\_PORC\_MVIC\_VM |
|  | 6 | LUNGE\_PORC\_MVIC\_RF |
| 3 | 1 | SQ\_PORC\_MVIC\_GMED |
|  | 2 | SQ\_PORC\_MVIC\_GMAX |
|  | 3 | SQ\_PORC\_MVIC\_BF |
|  | 4 | SQ\_PORC\_MVIC\_VL |
|  | 5 | SQ\_PORC\_MVIC\_VM |
|  | 6 | SQ\_PORC\_MVIC\_RF |
|  |  |  |

Modelo lineal general

Estadísticos descriptivosEstadísticos descriptivos, tabla, 1 niveles de cabeceras de columna y 1 niveles de cabeceras de fila, tabla con 4 columnas y 20 filas

|  |  |  |  |
| --- | --- | --- | --- |
|  | Media | Desv. Desviación | N |
| STEP\_UP\_PORC\_MVIC\_GMED | 30,54 | 11,180 | 20 |
| STEP\_UP\_PORC\_MVIC\_GMAX | 27,79 | 14,093 | 20 |
| STEP\_UP\_PORC\_MVIC\_BF | 18,12 | 10,123 | 20 |
| STEP\_UP\_PORC\_MVIC\_VL | 55,47 | 20,230 | 20 |
| STEP\_UP\_PORC\_MVIC\_VM | 52,76 | 22,889 | 20 |
| STEP\_UP\_PORC\_MVIC\_RF | 31,45 | 10,213 | 20 |
| LUNGE\_PORC\_MVIC\_GMED | 30,92 | 9,449 | 20 |
| LUNGE\_PORC\_MVIC\_GMAX | 30,57 | 11,120 | 20 |
| LUNGE\_PORC\_MVIC\_BF | 19,15 | 11,143 | 20 |
| LUNGE\_PORC\_MVIC\_VL | 66,88 | 24,628 | 20 |
| LUNGE\_PORC\_MVIC\_VM | 61,44 | 36,152 | 20 |
| LUNGE\_PORC\_MVIC\_RF | 28,45 | 11,807 | 20 |
| SQ\_PORC\_MVIC\_GMED | 58,37 | 26,763 | 20 |
| SQ\_PORC\_MVIC\_GMAX | 50,88 | 18,949 | 20 |
| SQ\_PORC\_MVIC\_BF | 29,72 | 15,226 | 20 |
| SQ\_PORC\_MVIC\_VL | 100,15 | 33,571 | 20 |
| SQ\_PORC\_MVIC\_VM | 85,02 | 44,504 | 20 |
| SQ\_PORC\_MVIC\_RF | 33,76 | 12,938 | 20 |
|  |  |  |  |

Modelo lineal general

Pruebas multivarianteaPruebas multivariante, tabla, 1 niveles de cabeceras de columna y 2 niveles de cabeceras de fila, tabla con 10 columnas y 17 filas

|  |  |  |  |  |  |  |  |  |  |
| --- | --- | --- | --- | --- | --- | --- | --- | --- | --- |
| Efecto | | Valor | F | gl de hipótesis | gl de error | Sig. | Eta parcial al cuadrado | Parámetro sin centralidad | Potencia observadac |
| Ejerc | Traza de Pillai | ,855 | 53,265b | 2,000 | 18,000 | ,000 | ,855 | 106,530 | 1,000 |
|  | Lambda de Wilks | ,145 | 53,265b | 2,000 | 18,000 | ,000 | ,855 | 106,530 | 1,000 |
|  | Traza de Hotelling | 5,918 | 53,265b | 2,000 | 18,000 | ,000 | ,855 | 106,530 | 1,000 |
|  | Raíz mayor de Roy | 5,918 | 53,265b | 2,000 | 18,000 | ,000 | ,855 | 106,530 | 1,000 |
| Muscu | Traza de Pillai | ,868 | 19,802b | 5,000 | 15,000 | ,000 | ,868 | 99,008 | 1,000 |
|  | Lambda de Wilks | ,132 | 19,802b | 5,000 | 15,000 | ,000 | ,868 | 99,008 | 1,000 |
|  | Traza de Hotelling | 6,601 | 19,802b | 5,000 | 15,000 | ,000 | ,868 | 99,008 | 1,000 |
|  | Raíz mayor de Roy | 6,601 | 19,802b | 5,000 | 15,000 | ,000 | ,868 | 99,008 | 1,000 |
| Ejerc \* Muscu | Traza de Pillai | ,902 | 9,157b | 10,000 | 10,000 | ,001 | ,902 | 91,573 | ,998 |
|  | Lambda de Wilks | ,098 | 9,157b | 10,000 | 10,000 | ,001 | ,902 | 91,573 | ,998 |
|  | Traza de Hotelling | 9,157 | 9,157b | 10,000 | 10,000 | ,001 | ,902 | 91,573 | ,998 |
|  | Raíz mayor de Roy | 9,157 | 9,157b | 10,000 | 10,000 | ,001 | ,902 | 91,573 | ,998 |
|  |  |  |  |  |  |  |  |  |  |
| --- | --- | --- | --- | --- | --- | --- | --- | --- | --- |
| a. Diseño : Intersección   Diseño intra-sujetos: Ejerc + Muscu + Ejerc \* Muscu | | | | | | | | | |
| b. Estadístico exacto | | | | | | | | | |
| c. Se ha calculado utilizando alpha = ,05 | | | | | | | | | |
|  |  |  |  |  |  |  |  |  |  |

Modelo lineal general

Prueba de esfericidad de MauchlyaPrueba de esfericidad de Mauchly, tabla, Medida, MEASURE\_1, 1 capas, 2 niveles de cabeceras de columna y 1 niveles de cabeceras de fila, tabla con 8 columnas y 10 filas

| |  |  |  | | --- | --- | --- | | Medida: | MEASURE\_1 | MEASURE\_1 | | | | | | | | |
|  |  |  |  |  |  |  |  |
| --- | --- | --- | --- | --- | --- | --- | --- |
| Efecto intra-sujetos | W de Mauchly | Aprox. Chi-cuadrado | gl | Sig. | Épsilonb | | |
|  |  |  |  |  | Greenhouse-Geisser | Huynh-Feldt | Límite inferior |
| Ejerc | ,814 | 3,705 | 2 | ,157 | ,843 | ,916 | ,500 |
| Muscu | ,059 | 48,260 | 14 | ,000 | ,493 | ,572 | ,200 |
| Ejerc \* Muscu | ,000 | 135,644 | 54 | ,000 | ,486 | ,673 | ,100 |
|  |  |  |  |  |  |  |  |
| --- | --- | --- | --- | --- | --- | --- | --- |
| Prueba la hipótesis nula de que la matriz de covarianzas de error de las variables dependientes con transformación ortonormalizada es proporcional a una matriz de identidad. | | | | | | | |
| a. Diseño : Intersección   Diseño intra-sujetos: Ejerc + Muscu + Ejerc \* Muscu | | | | | | | |
| b. Se puede utilizar para ajustar los grados de libertad para las pruebas promedio de significación. Las pruebas corregidas se visualizan en la tabla de pruebas de efectos intra-sujetos. | | | | | | | |
|  |  |  |  |  |  |  |  |

Modelo lineal general

Pruebas de efectos intra-sujetosPruebas de efectos intra-sujetos, tabla, Medida, MEASURE\_1, 1 capas, 1 niveles de cabeceras de columna y 2 niveles de cabeceras de fila, tabla con 10 columnas y 28 filas

| |  |  |  | | --- | --- | --- | | Medida: | MEASURE\_1 | MEASURE\_1 | | | | | | | | | | |
|  |  |  |  |  |  |  |  |  |  |
| --- | --- | --- | --- | --- | --- | --- | --- | --- | --- |
| Origen | | Tipo III de suma de cuadrados | gl | Media cuadrática | F | Sig. | Eta parcial al cuadrado | Parámetro sin centralidad | Potencia observadaa |
| Ejerc | Esfericidad asumida | 38974,586 | 2 | 19487,293 | 75,411 | ,000 | ,799 | 150,822 | 1,000 |
|  | Greenhouse-Geisser | 38974,586 | 1,686 | 23112,236 | 75,411 | ,000 | ,799 | 127,167 | 1,000 |
|  | Huynh-Feldt | 38974,586 | 1,832 | 21269,174 | 75,411 | ,000 | ,799 | 138,187 | 1,000 |
|  | Límite inferior | 38974,586 | 1,000 | 38974,586 | 75,411 | ,000 | ,799 | 75,411 | 1,000 |
| Error(Ejerc) | Esfericidad asumida | 9819,743 | 38 | 258,414 |  |  |  |  |  |
|  | Greenhouse-Geisser | 9819,743 | 32,040 | 306,483 |  |  |  |  |  |
|  | Huynh-Feldt | 9819,743 | 34,816 | 282,043 |  |  |  |  |  |
|  | Límite inferior | 9819,743 | 19,000 | 516,829 |  |  |  |  |  |
| Muscu | Esfericidad asumida | 126706,488 | 5 | 25341,298 | 28,022 | ,000 | ,596 | 140,109 | 1,000 |
|  | Greenhouse-Geisser | 126706,488 | 2,463 | 51441,149 | 28,022 | ,000 | ,596 | 69,022 | 1,000 |
|  | Huynh-Feldt | 126706,488 | 2,858 | 44333,655 | 28,022 | ,000 | ,596 | 80,087 | 1,000 |
|  | Límite inferior | 126706,488 | 1,000 | 126706,488 | 28,022 | ,000 | ,596 | 28,022 | ,999 |
| Error(Muscu) | Esfericidad asumida | 85912,390 | 95 | 904,341 |  |  |  |  |  |
|  | Greenhouse-Geisser | 85912,390 | 46,800 | 1835,752 |  |  |  |  |  |
|  | Huynh-Feldt | 85912,390 | 54,302 | 1582,111 |  |  |  |  |  |
|  | Límite inferior | 85912,390 | 19,000 | 4521,705 |  |  |  |  |  |
| Ejerc \* Muscu | Esfericidad asumida | 12214,411 | 10 | 1221,441 | 13,283 | ,000 | ,411 | 132,833 | 1,000 |
|  | Greenhouse-Geisser | 12214,411 | 4,861 | 2512,779 | 13,283 | ,000 | ,411 | 64,569 | 1,000 |
|  | Huynh-Feldt | 12214,411 | 6,734 | 1813,732 | 13,283 | ,000 | ,411 | 89,455 | 1,000 |
|  | Límite inferior | 12214,411 | 1,000 | 12214,411 | 13,283 | ,002 | ,411 | 13,283 | ,933 |
| Error(Ejerc\*Muscu) | Esfericidad asumida | 17471,069 | 190 | 91,953 |  |  |  |  |  |
|  | Greenhouse-Geisser | 17471,069 | 92,357 | 189,168 |  |  |  |  |  |
|  | Huynh-Feldt | 17471,069 | 127,954 | 136,542 |  |  |  |  |  |
|  | Límite inferior | 17471,069 | 19,000 | 919,530 |  |  |  |  |  |
|  |  |  |  |  |  |  |  |  |  |
| --- | --- | --- | --- | --- | --- | --- | --- | --- | --- |
| a. Se ha calculado utilizando alpha = ,05 | | | | | | | | | |
|  |  |  |  |  |  |  |  |  |  |

Modelo lineal general

Pruebas de contrastes intra-sujetosPruebas de contrastes intra-sujetos, tabla, Medida, MEASURE\_1, 1 capas, 1 niveles de cabeceras de columna y 3 niveles de cabeceras de fila, tabla con 11 columnas y 38 filas

| |  |  |  | | --- | --- | --- | | Medida: | MEASURE\_1 | MEASURE\_1 | | | | | | | | | | | |  |
|  |  |  |  |  |  |  |  |  |  |  |  |
| --- | --- | --- | --- | --- | --- | --- | --- | --- | --- | --- | --- |
| Origen | Ejerc | Muscu | Tipo III de suma de cuadrados | gl | Media cuadrática | F | Sig. | Eta parcial al cuadrado | Parámetro sin centralidad | Potencia observadaa |  |
| Ejerc | Lineal |  | 33505,920 | 1 | 33505,920 | 92,217 | ,000 | ,829 | 92,217 | 1,000 |  |
|  | Cuadrático |  | 5468,667 | 1 | 5468,667 | 35,629 | ,000 | ,652 | 35,629 | 1,000 |  |
| Error(Ejerc) | Lineal |  | 6903,440 | 19 | 363,339 |  |  |  |  |  |  |
|  | Cuadrático |  | 2916,303 | 19 | 153,490 |  |  |  |  |  |  |
| Muscu |  | Lineal | 8265,435 | 1 | 8265,435 | 6,637 | ,018 | ,259 | 6,637 | ,686 |  |
|  |  | Cuadrático | 12632,368 | 1 | 12632,368 | 19,131 | ,000 | ,502 | 19,131 | ,986 |  |
|  |  | Cúbico | 70807,938 | 1 | 70807,938 | 58,858 | ,000 | ,756 | 58,858 | 1,000 |  |
|  |  | Orden 4 | 4207,489 | 1 | 4207,489 | 6,426 | ,020 | ,253 | 6,426 | ,672 |  |
|  |  | Orden 5 | 30793,257 | 1 | 30793,257 | 40,613 | ,000 | ,681 | 40,613 | 1,000 |  |
| Error(Muscu) |  | Lineal | 23661,371 | 19 | 1245,335 |  |  |  |  |  |  |
|  |  | Cuadrático | 12546,154 | 19 | 660,324 |  |  |  |  |  |  |
|  |  | Cúbico | 22857,615 | 19 | 1203,032 |  |  |  |  |  |  |
|  |  | Orden 4 | 12441,100 | 19 | 654,795 |  |  |  |  |  |  |
|  |  | Orden 5 | 14406,149 | 19 | 758,218 |  |  |  |  |  |  |
| Ejerc \* Muscu | Lineal | Lineal | 642,185 | 1 | 642,185 | 5,433 | ,031 | ,222 | 5,433 | ,600 |  |
|  |  | Cuadrático | 2004,541 | 1 | 2004,541 | 15,277 | ,001 | ,446 | 15,277 | ,959 |  |
|  |  | Cúbico | 5835,293 | 1 | 5835,293 | 28,515 | ,000 | ,600 | 28,515 | ,999 |  |
|  |  | Orden 4 | 194,964 | 1 | 194,964 | 1,372 | ,256 | ,067 | 1,372 | ,199 |  |
|  |  | Orden 5 | 2672,306 | 1 | 2672,306 | 25,780 | ,000 | ,576 | 25,780 | ,998 |  |
|  | Cuadrático | Lineal | 379,435 | 1 | 379,435 | 5,088 | ,036 | ,211 | 5,088 | ,572 |  |
|  |  | Cuadrático | 14,115 | 1 | 14,115 | ,388 | ,541 | ,020 | ,388 | ,091 |  |
|  |  | Cúbico | 288,594 | 1 | 288,594 | 6,459 | ,020 | ,254 | 6,459 | ,674 |  |
|  |  | Orden 4 | ,093 | 1 | ,093 | ,002 | ,962 | ,000 | ,002 | ,050 |  |
|  |  | Orden 5 | 182,886 | 1 | 182,886 | 7,813 | ,012 | ,291 | 7,813 | ,755 |  |
| Error(Ejerc\*Muscu) | Lineal | Lineal | 2245,951 | 19 | 118,208 |  |  |  |  |  |  |
|  |  | Cuadrático | 2493,019 | 19 | 131,212 |  |  |  |  |  |  |
|  |  | Cúbico | 3888,097 | 19 | 204,637 |  |  |  |  |  |  |
|  |  | Orden 4 | 2700,777 | 19 | 142,146 |  |  |  |  |  |  |
|  |  | Orden 5 | 1969,503 | 19 | 103,658 |  |  |  |  |  |  |
|  | Cuadrático | Lineal | 1416,894 | 19 | 74,573 |  |  |  |  |  |  |
|  |  | Cuadrático | 691,115 | 19 | 36,374 |  |  |  |  |  |  |
|  |  | Cúbico | 848,995 | 19 | 44,684 |  |  |  |  |  |  |
|  |  | Orden 4 | 771,946 | 19 | 40,629 |  |  |  |  |  |  |
|  |  | Orden 5 | 444,772 | 19 | 23,409 |  |  |  |  |  |  |
|  |  |  |  |  |  |  |  |  |  |  |  |
| --- | --- | --- | --- | --- | --- | --- | --- | --- | --- | --- | --- |
| a. Se ha calculado utilizando alpha = ,05 | | | | | | | | | | |  |
|  |  |  |  |  |  |  |  |  |  |  |  |

Modelo lineal general

Pruebas de efectos inter-sujetosPruebas de efectos inter-sujetos, tabla, Medida, MEASURE\_1, Variable transformada, Promedio, 1 capas, 1 niveles de cabeceras de columna y 1 niveles de cabeceras de fila, tabla con 9 columnas y 7 filas

| |  |  |  | | --- | --- | --- | | Medida: | MEASURE\_1 | MEASURE\_1 | | Variable transformada: | Promedio | Promedio | | | | | | | | | |
|  |  |  |  |  |  |  |  |  |
| --- | --- | --- | --- | --- | --- | --- | --- | --- |
| Origen | Tipo III de suma de cuadrados | gl | Media cuadrática | F | Sig. | Eta parcial al cuadrado | Parámetro sin centralidad | Potencia observadaa |
| Intersección | 731613,811 | 1 | 731613,811 | 295,295 | ,000 | ,940 | 295,295 | 1,000 |
| Error | 47073,792 | 19 | 2477,568 |  |  |  |  |  |
|  |  |  |  |  |  |  |  |  |
| --- | --- | --- | --- | --- | --- | --- | --- | --- |
| a. Se ha calculado utilizando alpha = ,05 | | | | | | | | |
|  |  |  |  |  |  |  |  |  |

1. Ejerc

EstimacionesEstimaciones, tabla, Medida, MEASURE\_1, 1 capas, 2 niveles de cabeceras de columna y 1 niveles de cabeceras de fila, tabla con 5 columnas y 7 filas

| |  |  |  | | --- | --- | --- | | Medida: | MEASURE\_1 | MEASURE\_1 | | | | | |
|  |  |  |  |  |
| --- | --- | --- | --- | --- |
| Ejerc | Media | Desv. Error | Intervalo de confianza al 95% | |
|  |  |  | Límite inferior | Límite superior |
| 1 | 36,021 | 2,133 | 31,557 | 40,485 |
| 2 | 39,569 | 2,675 | 33,970 | 45,167 |
| 3 | 59,652 | 3,640 | 52,033 | 67,271 |
|  |  |  |  |  |

1. Ejerc

Comparaciones por parejasComparaciones por parejas, tabla, Medida, MEASURE\_1, 1 capas, 2 niveles de cabeceras de columna y 2 niveles de cabeceras de fila, tabla con 7 columnas y 13 filas

| |  |  |  | | --- | --- | --- | | Medida: | MEASURE\_1 | MEASURE\_1 | | | | | | | |
|  |  |  |  |  |  |  |
| --- | --- | --- | --- | --- | --- | --- |
| (I) Ejerc | (J) Ejerc | Diferencia de medias (I-J) | Desv. Error | Sig.b | 95% de intervalo de confianza para diferenciab | |
|  |  |  |  |  | Límite inferior | Límite superior |
| 1 | 2 | -3,548 | 1,700 | ,152 | -8,010 | ,915 |
|  | 3 | -23,631\* | 2,461 | ,000 | -30,091 | -17,171 |
| 2 | 1 | 3,548 | 1,700 | ,152 | -,915 | 8,010 |
|  | 3 | -20,083\* | 1,994 | ,000 | -25,317 | -14,850 |
| 3 | 1 | 23,631\* | 2,461 | ,000 | 17,171 | 30,091 |
|  | 2 | 20,083\* | 1,994 | ,000 | 14,850 | 25,317 |
|  |  |  |  |  |  |  |
| --- | --- | --- | --- | --- | --- | --- |
| Se basa en medias marginales estimadas | | | | | | |
| \*. La diferencia de medias es significativa en el nivel ,05. | | | | | | |
| b. Ajuste para varias comparaciones: Bonferroni. | | | | | | |
|  |  |  |  |  |  |  |

1. Ejerc

Pruebas multivariantePruebas multivariante, tabla, 1 niveles de cabeceras de columna y 1 niveles de cabeceras de fila, tabla con 9 columnas y 9 filas

|  |  |  |  |  |  |  |  |  |
| --- | --- | --- | --- | --- | --- | --- | --- | --- |
|  | Valor | F | gl de hipótesis | gl de error | Sig. | Eta parcial al cuadrado | Parámetro sin centralidad | Potencia observadab |
| Traza de Pillai | ,855 | 53,265a | 2,000 | 18,000 | ,000 | ,855 | 106,530 | 1,000 |
| Lambda de Wilks | ,145 | 53,265a | 2,000 | 18,000 | ,000 | ,855 | 106,530 | 1,000 |
| Traza de Hotelling | 5,918 | 53,265a | 2,000 | 18,000 | ,000 | ,855 | 106,530 | 1,000 |
| Raíz mayor de Roy | 5,918 | 53,265a | 2,000 | 18,000 | ,000 | ,855 | 106,530 | 1,000 |
|  |  |  |  |  |  |  |  |  |
| --- | --- | --- | --- | --- | --- | --- | --- | --- |
| Cada F prueba el efecto multivariante de Ejerc. Estas pruebas se basan en las comparaciones por parejas linealmente independientes entre las medias marginales estimadas. | | | | | | | | |
| a. Estadístico exacto | | | | | | | | |
| b. Se ha calculado utilizando alpha = ,05 | | | | | | | | |
|  |  |  |  |  |  |  |  |  |

2. Muscu

EstimacionesEstimaciones, tabla, Medida, MEASURE\_1, 1 capas, 2 niveles de cabeceras de columna y 1 niveles de cabeceras de fila, tabla con 5 columnas y 10 filas

| |  |  |  | | --- | --- | --- | | Medida: | MEASURE\_1 | MEASURE\_1 | | | | | |
|  |  |  |  |  |
| --- | --- | --- | --- | --- |
| Muscu | Media | Desv. Error | Intervalo de confianza al 95% | |
|  |  |  | Límite inferior | Límite superior |
| 1 | 39,944 | 3,209 | 33,226 | 46,661 |
| 2 | 36,415 | 2,937 | 30,268 | 42,562 |
| 3 | 22,332 | 2,599 | 16,894 | 27,771 |
| 4 | 74,164 | 5,412 | 62,836 | 85,492 |
| 5 | 66,408 | 7,474 | 50,765 | 82,052 |
| 6 | 31,221 | 2,413 | 26,171 | 36,271 |
|  |  |  |  |  |

2. Muscu

Comparaciones por parejasComparaciones por parejas, tabla, Medida, MEASURE\_1, 1 capas, 2 niveles de cabeceras de columna y 2 niveles de cabeceras de fila, tabla con 7 columnas y 37 filas

| |  |  |  | | --- | --- | --- | | Medida: | MEASURE\_1 | MEASURE\_1 | | | | | | | |
|  |  |  |  |  |  |  |
| --- | --- | --- | --- | --- | --- | --- |
| (I) Muscu | (J) Muscu | Diferencia de medias (I-J) | Desv. Error | Sig.b | 95% de intervalo de confianza para diferenciab | |
|  |  |  |  |  | Límite inferior | Límite superior |
| 1 | 2 | 3,529 | 3,431 | 1,000 | -7,979 | 15,037 |
|  | 3 | 17,611\* | 3,857 | ,003 | 4,673 | 30,549 |
|  | 4 | -34,220\* | 6,480 | ,001 | -55,956 | -12,485 |
|  | 5 | -26,465 | 8,334 | ,075 | -54,416 | 1,487 |
|  | 6 | 8,723 | 4,546 | 1,000 | -6,523 | 23,969 |
| 2 | 1 | -3,529 | 3,431 | 1,000 | -15,037 | 7,979 |
|  | 3 | 14,082\* | 3,054 | ,003 | 3,840 | 24,325 |
|  | 4 | -37,749\* | 5,671 | ,000 | -56,769 | -18,730 |
|  | 5 | -29,993\* | 7,429 | ,011 | -54,910 | -5,077 |
|  | 6 | 5,194 | 3,068 | 1,000 | -5,095 | 15,483 |
| 3 | 1 | -17,611\* | 3,857 | ,003 | -30,549 | -4,673 |
|  | 2 | -14,082\* | 3,054 | ,003 | -24,325 | -3,840 |
|  | 4 | -51,832\* | 5,571 | ,000 | -70,518 | -33,146 |
|  | 5 | -44,076\* | 6,324 | ,000 | -65,286 | -22,866 |
|  | 6 | -8,889\* | 2,447 | ,027 | -17,097 | -,681 |
| 4 | 1 | 34,220\* | 6,480 | ,001 | 12,485 | 55,956 |
|  | 2 | 37,749\* | 5,671 | ,000 | 18,730 | 56,769 |
|  | 3 | 51,832\* | 5,571 | ,000 | 33,146 | 70,518 |
|  | 5 | 7,756 | 6,080 | 1,000 | -12,636 | 28,148 |
|  | 6 | 42,943\* | 5,095 | ,000 | 25,855 | 60,031 |
| 5 | 1 | 26,465 | 8,334 | ,075 | -1,487 | 54,416 |
|  | 2 | 29,993\* | 7,429 | ,011 | 5,077 | 54,910 |
|  | 3 | 44,076\* | 6,324 | ,000 | 22,866 | 65,286 |
|  | 4 | -7,756 | 6,080 | 1,000 | -28,148 | 12,636 |
|  | 6 | 35,187\* | 6,885 | ,001 | 12,096 | 58,279 |
| 6 | 1 | -8,723 | 4,546 | 1,000 | -23,969 | 6,523 |
|  | 2 | -5,194 | 3,068 | 1,000 | -15,483 | 5,095 |
|  | 3 | 8,889\* | 2,447 | ,027 | ,681 | 17,097 |
|  | 4 | -42,943\* | 5,095 | ,000 | -60,031 | -25,855 |
|  | 5 | -35,187\* | 6,885 | ,001 | -58,279 | -12,096 |
|  |  |  |  |  |  |  |
| --- | --- | --- | --- | --- | --- | --- |
| Se basa en medias marginales estimadas | | | | | | |
| \*. La diferencia de medias es significativa en el nivel ,05. | | | | | | |
| b. Ajuste para varias comparaciones: Bonferroni. | | | | | | |
|  |  |  |  |  |  |  |

2. Muscu

Pruebas multivariantePruebas multivariante, tabla, 1 niveles de cabeceras de columna y 1 niveles de cabeceras de fila, tabla con 9 columnas y 9 filas

|  |  |  |  |  |  |  |  |  |
| --- | --- | --- | --- | --- | --- | --- | --- | --- |
|  | Valor | F | gl de hipótesis | gl de error | Sig. | Eta parcial al cuadrado | Parámetro sin centralidad | Potencia observadab |
| Traza de Pillai | ,868 | 19,802a | 5,000 | 15,000 | ,000 | ,868 | 99,008 | 1,000 |
| Lambda de Wilks | ,132 | 19,802a | 5,000 | 15,000 | ,000 | ,868 | 99,008 | 1,000 |
| Traza de Hotelling | 6,601 | 19,802a | 5,000 | 15,000 | ,000 | ,868 | 99,008 | 1,000 |
| Raíz mayor de Roy | 6,601 | 19,802a | 5,000 | 15,000 | ,000 | ,868 | 99,008 | 1,000 |
|  |  |  |  |  |  |  |  |  |
| --- | --- | --- | --- | --- | --- | --- | --- | --- |
| Cada F prueba el efecto multivariante de Muscu. Estas pruebas se basan en las comparaciones por parejas linealmente independientes entre las medias marginales estimadas. | | | | | | | | |
| a. Estadístico exacto | | | | | | | | |
| b. Se ha calculado utilizando alpha = ,05 | | | | | | | | |
|  |  |  |  |  |  |  |  |  |

3. Ejerc \* Muscu

EstimacionesEstimaciones, tabla, Medida, MEASURE\_1, 1 capas, 2 niveles de cabeceras de columna y 2 niveles de cabeceras de fila, tabla con 6 columnas y 22 filas

| |  |  |  | | --- | --- | --- | | Medida: | MEASURE\_1 | MEASURE\_1 | | | | | | |
|  |  |  |  |  |  |
| --- | --- | --- | --- | --- | --- |
| Ejerc | Muscu | Media | Desv. Error | Intervalo de confianza al 95% | |
|  |  |  |  | Límite inferior | Límite superior |
| 1 | 1 | 30,537 | 2,500 | 25,305 | 35,770 |
|  | 2 | 27,786 | 3,151 | 21,190 | 34,381 |
|  | 3 | 18,122 | 2,264 | 13,384 | 22,860 |
|  | 4 | 55,467 | 4,524 | 45,999 | 64,934 |
|  | 5 | 52,763 | 5,118 | 42,051 | 63,476 |
|  | 6 | 31,451 | 2,284 | 26,671 | 36,231 |
| 2 | 1 | 30,921 | 2,113 | 26,499 | 35,343 |
|  | 2 | 30,574 | 2,486 | 25,370 | 35,779 |
|  | 3 | 19,154 | 2,492 | 13,939 | 24,369 |
|  | 4 | 66,875 | 5,507 | 55,349 | 78,402 |
|  | 5 | 61,437 | 8,084 | 44,518 | 78,357 |
|  | 6 | 28,450 | 2,640 | 22,924 | 33,976 |
| 3 | 1 | 58,372 | 5,984 | 45,847 | 70,897 |
|  | 2 | 50,884 | 4,237 | 42,016 | 59,752 |
|  | 3 | 29,721 | 3,405 | 22,595 | 36,847 |
|  | 4 | 100,150 | 7,507 | 84,438 | 115,862 |
|  | 5 | 85,024 | 9,951 | 64,195 | 105,852 |
|  | 6 | 33,762 | 2,893 | 27,707 | 39,817 |
|  |  |  |  |  |  |

3. Ejerc \* Muscu

Comparaciones por parejasComparaciones por parejas, tabla, Medida, MEASURE\_1, 1 capas, 2 niveles de cabeceras de columna y 3 niveles de cabeceras de fila, tabla con 8 columnas y 97 filas

| |  |  |  | | --- | --- | --- | | Medida: | MEASURE\_1 | MEASURE\_1 | | | | | | | | |
|  |  |  |  |  |  |  |  |
| --- | --- | --- | --- | --- | --- | --- | --- |
| Ejerc | (I) Muscu | (J) Muscu | Diferencia de medias (I-J) | Desv. Error | Sig.b | 95% de intervalo de confianza para diferenciab | |
|  |  |  |  |  |  | Límite inferior | Límite superior |
| 1 | 1 | 2 | 2,752 | 2,842 | 1,000 | -6,779 | 12,283 |
|  |  | 3 | 12,416\* | 3,174 | ,014 | 1,771 | 23,060 |
|  |  | 4 | -24,929\* | 5,067 | ,001 | -41,924 | -7,934 |
|  |  | 5 | -22,226\* | 5,787 | ,017 | -41,635 | -2,816 |
|  |  | 6 | -,913 | 3,747 | 1,000 | -13,480 | 11,653 |
|  | 2 | 1 | -2,752 | 2,842 | 1,000 | -12,283 | 6,779 |
|  |  | 3 | 9,664 | 2,911 | ,054 | -,098 | 19,426 |
|  |  | 4 | -27,681\* | 5,072 | ,000 | -44,692 | -10,670 |
|  |  | 5 | -24,978\* | 5,724 | ,005 | -44,176 | -5,780 |
|  |  | 6 | -3,665 | 3,404 | 1,000 | -15,083 | 7,753 |
|  | 3 | 1 | -12,416\* | 3,174 | ,014 | -23,060 | -1,771 |
|  |  | 2 | -9,664 | 2,911 | ,054 | -19,426 | ,098 |
|  |  | 4 | -37,345\* | 4,608 | ,000 | -52,800 | -21,889 |
|  |  | 5 | -34,641\* | 4,404 | ,000 | -49,412 | -19,871 |
|  |  | 6 | -13,329\* | 2,447 | ,000 | -21,537 | -5,121 |
|  | 4 | 1 | 24,929\* | 5,067 | ,001 | 7,934 | 41,924 |
|  |  | 2 | 27,681\* | 5,072 | ,000 | 10,670 | 44,692 |
|  |  | 3 | 37,345\* | 4,608 | ,000 | 21,889 | 52,800 |
|  |  | 5 | 2,703 | 4,586 | 1,000 | -12,677 | 18,084 |
|  |  | 6 | 24,016\* | 4,099 | ,000 | 10,267 | 37,765 |
|  | 5 | 1 | 22,226\* | 5,787 | ,017 | 2,816 | 41,635 |
|  |  | 2 | 24,978\* | 5,724 | ,005 | 5,780 | 44,176 |
|  |  | 3 | 34,641\* | 4,404 | ,000 | 19,871 | 49,412 |
|  |  | 4 | -2,703 | 4,586 | 1,000 | -18,084 | 12,677 |
|  |  | 6 | 21,312\* | 4,578 | ,003 | 5,959 | 36,666 |
|  | 6 | 1 | ,913 | 3,747 | 1,000 | -11,653 | 13,480 |
|  |  | 2 | 3,665 | 3,404 | 1,000 | -7,753 | 15,083 |
|  |  | 3 | 13,329\* | 2,447 | ,000 | 5,121 | 21,537 |
|  |  | 4 | -24,016\* | 4,099 | ,000 | -37,765 | -10,267 |
|  |  | 5 | -21,312\* | 4,578 | ,003 | -36,666 | -5,959 |
| 2 | 1 | 2 | ,347 | 3,033 | 1,000 | -9,825 | 10,519 |
|  |  | 3 | 11,767\* | 3,412 | ,040 | ,324 | 23,211 |
|  |  | 4 | -35,954\* | 6,349 | ,000 | -57,249 | -14,659 |
|  |  | 5 | -30,516\* | 8,976 | ,045 | -60,620 | -,412 |
|  |  | 6 | 2,471 | 3,684 | 1,000 | -9,885 | 14,828 |
|  | 2 | 1 | -,347 | 3,033 | 1,000 | -10,519 | 9,825 |
|  |  | 3 | 11,421\* | 2,636 | ,005 | 2,581 | 20,260 |
|  |  | 4 | -36,301\* | 5,783 | ,000 | -55,696 | -16,906 |
|  |  | 5 | -30,863\* | 8,198 | ,020 | -58,359 | -3,366 |
|  |  | 6 | 2,125 | 2,561 | 1,000 | -6,467 | 10,716 |
|  | 3 | 1 | -11,767\* | 3,412 | ,040 | -23,211 | -,324 |
|  |  | 2 | -11,421\* | 2,636 | ,005 | -20,260 | -2,581 |
|  |  | 4 | -47,722\* | 5,197 | ,000 | -65,152 | -30,291 |
|  |  | 5 | -42,283\* | 6,525 | ,000 | -64,168 | -20,399 |
|  |  | 6 | -9,296\* | 2,338 | ,012 | -17,137 | -1,456 |
|  | 4 | 1 | 35,954\* | 6,349 | ,000 | 14,659 | 57,249 |
|  |  | 2 | 36,301\* | 5,783 | ,000 | 16,906 | 55,696 |
|  |  | 3 | 47,722\* | 5,197 | ,000 | 30,291 | 65,152 |
|  |  | 5 | 5,438 | 6,124 | 1,000 | -15,100 | 25,977 |
|  |  | 6 | 38,426\* | 4,824 | ,000 | 22,246 | 54,605 |
|  | 5 | 1 | 30,516\* | 8,976 | ,045 | ,412 | 60,620 |
|  |  | 2 | 30,863\* | 8,198 | ,020 | 3,366 | 58,359 |
|  |  | 3 | 42,283\* | 6,525 | ,000 | 20,399 | 64,168 |
|  |  | 4 | -5,438 | 6,124 | 1,000 | -25,977 | 15,100 |
|  |  | 6 | 32,987\* | 7,363 | ,004 | 8,293 | 57,682 |
|  | 6 | 1 | -2,471 | 3,684 | 1,000 | -14,828 | 9,885 |
|  |  | 2 | -2,125 | 2,561 | 1,000 | -10,716 | 6,467 |
|  |  | 3 | 9,296\* | 2,338 | ,012 | 1,456 | 17,137 |
|  |  | 4 | -38,426\* | 4,824 | ,000 | -54,605 | -22,246 |
|  |  | 5 | -32,987\* | 7,363 | ,004 | -57,682 | -8,293 |
| 3 | 1 | 2 | 7,488 | 6,123 | 1,000 | -13,049 | 28,025 |
|  |  | 3 | 28,651\* | 6,070 | ,002 | 8,291 | 49,011 |
|  |  | 4 | -41,778\* | 9,388 | ,004 | -73,267 | -10,289 |
|  |  | 5 | -26,652 | 11,057 | ,393 | -63,737 | 10,434 |
|  |  | 6 | 24,610\* | 7,148 | ,041 | ,636 | 48,584 |
|  | 2 | 1 | -7,488 | 6,123 | 1,000 | -28,025 | 13,049 |
|  |  | 3 | 21,162\* | 4,643 | ,003 | 5,590 | 36,735 |
|  |  | 4 | -49,266\* | 7,708 | ,000 | -75,119 | -23,414 |
|  |  | 5 | -34,140\* | 9,363 | ,026 | -65,544 | -2,736 |
|  |  | 6 | 17,122\* | 4,746 | ,028 | 1,202 | 33,042 |
|  | 3 | 1 | -28,651\* | 6,070 | ,002 | -49,011 | -8,291 |
|  |  | 2 | -21,162\* | 4,643 | ,003 | -36,735 | -5,590 |
|  |  | 4 | -70,429\* | 7,937 | ,000 | -97,049 | -43,808 |
|  |  | 5 | -55,303\* | 8,926 | ,000 | -85,240 | -25,365 |
|  |  | 6 | -4,041 | 3,259 | 1,000 | -14,972 | 6,891 |
|  | 4 | 1 | 41,778\* | 9,388 | ,004 | 10,289 | 73,267 |
|  |  | 2 | 49,266\* | 7,708 | ,000 | 23,414 | 75,119 |
|  |  | 3 | 70,429\* | 7,937 | ,000 | 43,808 | 97,049 |
|  |  | 5 | 15,126 | 8,477 | 1,000 | -13,306 | 43,558 |
|  |  | 6 | 66,388\* | 7,588 | ,000 | 40,937 | 91,839 |
|  | 5 | 1 | 26,652 | 11,057 | ,393 | -10,434 | 63,737 |
|  |  | 2 | 34,140\* | 9,363 | ,026 | 2,736 | 65,544 |
|  |  | 3 | 55,303\* | 8,926 | ,000 | 25,365 | 85,240 |
|  |  | 4 | -15,126 | 8,477 | 1,000 | -43,558 | 13,306 |
|  |  | 6 | 51,262\* | 9,375 | ,000 | 19,817 | 82,707 |
|  | 6 | 1 | -24,610\* | 7,148 | ,041 | -48,584 | -,636 |
|  |  | 2 | -17,122\* | 4,746 | ,028 | -33,042 | -1,202 |
|  |  | 3 | 4,041 | 3,259 | 1,000 | -6,891 | 14,972 |
|  |  | 4 | -66,388\* | 7,588 | ,000 | -91,839 | -40,937 |
|  |  | 5 | -51,262\* | 9,375 | ,000 | -82,707 | -19,817 |
|  |  |  |  |  |  |  |  |
| --- | --- | --- | --- | --- | --- | --- | --- |
| Se basa en medias marginales estimadas | | | | | | | |
| \*. La diferencia de medias es significativa en el nivel ,05. | | | | | | | |
| b. Ajuste para varias comparaciones: Bonferroni. | | | | | | | |
|  |  |  |  |  |  |  |  |

3. Ejerc \* Muscu

Pruebas multivariantePruebas multivariante, tabla, 1 niveles de cabeceras de columna y 2 niveles de cabeceras de fila, tabla con 10 columnas y 17 filas

|  |  |  |  |  |  |  |  |  |  |
| --- | --- | --- | --- | --- | --- | --- | --- | --- | --- |
| Ejerc | | Valor | F | gl de hipótesis | gl de error | Sig. | Eta parcial al cuadrado | Parámetro sin centralidad | Potencia observadab |
| 1 | Traza de Pillai | ,849 | 16,890a | 5,000 | 15,000 | ,000 | ,849 | 84,451 | 1,000 |
|  | Lambda de Wilks | ,151 | 16,890a | 5,000 | 15,000 | ,000 | ,849 | 84,451 | 1,000 |
|  | Traza de Hotelling | 5,630 | 16,890a | 5,000 | 15,000 | ,000 | ,849 | 84,451 | 1,000 |
|  | Raíz mayor de Roy | 5,630 | 16,890a | 5,000 | 15,000 | ,000 | ,849 | 84,451 | 1,000 |
| 2 | Traza de Pillai | ,900 | 27,107a | 5,000 | 15,000 | ,000 | ,900 | 135,535 | 1,000 |
|  | Lambda de Wilks | ,100 | 27,107a | 5,000 | 15,000 | ,000 | ,900 | 135,535 | 1,000 |
|  | Traza de Hotelling | 9,036 | 27,107a | 5,000 | 15,000 | ,000 | ,900 | 135,535 | 1,000 |
|  | Raíz mayor de Roy | 9,036 | 27,107a | 5,000 | 15,000 | ,000 | ,900 | 135,535 | 1,000 |
| 3 | Traza de Pillai | ,840 | 15,696a | 5,000 | 15,000 | ,000 | ,840 | 78,479 | 1,000 |
|  | Lambda de Wilks | ,160 | 15,696a | 5,000 | 15,000 | ,000 | ,840 | 78,479 | 1,000 |
|  | Traza de Hotelling | 5,232 | 15,696a | 5,000 | 15,000 | ,000 | ,840 | 78,479 | 1,000 |
|  | Raíz mayor de Roy | 5,232 | 15,696a | 5,000 | 15,000 | ,000 | ,840 | 78,479 | 1,000 |
|  |  |  |  |  |  |  |  |  |  |
| --- | --- | --- | --- | --- | --- | --- | --- | --- | --- |
| Cada F prueba los efectos simples multivariantes de Muscu dentro de cada combinación de niveles de los otros efectos mostrados. Estas pruebas se basan en las comparaciones por parejas linealmente independientes entre las medias marginales estimadas. | | | | | | | | | |
| a. Estadístico exacto | | | | | | | | | |
| b. Se ha calculado utilizando alpha = ,05 | | | | | | | | | |
|  |  |  |  |  |  |  |  |  |  |

3. Ejerc \* Muscu

Comparaciones por parejasComparaciones por parejas, tabla, Medida, MEASURE\_1, 1 capas, 2 niveles de cabeceras de columna y 3 niveles de cabeceras de fila, tabla con 8 columnas y 43 filas

| |  |  |  | | --- | --- | --- | | Medida: | MEASURE\_1 | MEASURE\_1 | | | | | | | | |
|  |  |  |  |  |  |  |  |
| --- | --- | --- | --- | --- | --- | --- | --- |
| Muscu | (I) Ejerc | (J) Ejerc | Diferencia de medias (I-J) | Desv. Error | Sig.b | 95% de intervalo de confianza para diferenciab | |
|  |  |  |  |  |  | Límite inferior | Límite superior |
| 1 | 1 | 2 | -,384 | 1,656 | 1,000 | -4,731 | 3,963 |
|  |  | 3 | -27,835\* | 4,564 | ,000 | -39,816 | -15,854 |
|  | 2 | 1 | ,384 | 1,656 | 1,000 | -3,963 | 4,731 |
|  |  | 3 | -27,451\* | 4,827 | ,000 | -40,122 | -14,780 |
|  | 3 | 1 | 27,835\* | 4,564 | ,000 | 15,854 | 39,816 |
|  |  | 2 | 27,451\* | 4,827 | ,000 | 14,780 | 40,122 |
| 2 | 1 | 2 | -2,789 | 1,964 | ,516 | -7,945 | 2,368 |
|  |  | 3 | -23,098\* | 3,349 | ,000 | -31,889 | -14,307 |
|  | 2 | 1 | 2,789 | 1,964 | ,516 | -2,368 | 7,945 |
|  |  | 3 | -20,309\* | 3,081 | ,000 | -28,398 | -12,221 |
|  | 3 | 1 | 23,098\* | 3,349 | ,000 | 14,307 | 31,889 |
|  |  | 2 | 20,309\* | 3,081 | ,000 | 12,221 | 28,398 |
| 3 | 1 | 2 | -1,032 | 1,300 | 1,000 | -4,445 | 2,381 |
|  |  | 3 | -11,599\* | 1,935 | ,000 | -16,680 | -6,519 |
|  | 2 | 1 | 1,032 | 1,300 | 1,000 | -2,381 | 4,445 |
|  |  | 3 | -10,568\* | 1,602 | ,000 | -14,773 | -6,363 |
|  | 3 | 1 | 11,599\* | 1,935 | ,000 | 6,519 | 16,680 |
|  |  | 2 | 10,568\* | 1,602 | ,000 | 6,363 | 14,773 |
| 4 | 1 | 2 | -11,409\* | 3,388 | ,010 | -20,304 | -2,514 |
|  |  | 3 | -44,683\* | 5,667 | ,000 | -59,561 | -29,806 |
|  | 2 | 1 | 11,409\* | 3,388 | ,010 | 2,514 | 20,304 |
|  |  | 3 | -33,275\* | 3,767 | ,000 | -43,163 | -23,386 |
|  | 3 | 1 | 44,683\* | 5,667 | ,000 | 29,806 | 59,561 |
|  |  | 2 | 33,275\* | 3,767 | ,000 | 23,386 | 43,163 |
| 5 | 1 | 2 | -8,674 | 4,469 | ,202 | -20,406 | 3,059 |
|  |  | 3 | -32,261\* | 5,878 | ,000 | -47,690 | -16,831 |
|  | 2 | 1 | 8,674 | 4,469 | ,202 | -3,059 | 20,406 |
|  |  | 3 | -23,587\* | 3,799 | ,000 | -33,559 | -13,615 |
|  | 3 | 1 | 32,261\* | 5,878 | ,000 | 16,831 | 47,690 |
|  |  | 2 | 23,587\* | 3,799 | ,000 | 13,615 | 33,559 |
| 6 | 1 | 2 | 3,001 | 1,660 | ,260 | -1,357 | 7,359 |
|  |  | 3 | -2,311 | 1,965 | ,762 | -7,469 | 2,847 |
|  | 2 | 1 | -3,001 | 1,660 | ,260 | -7,359 | 1,357 |
|  |  | 3 | -5,312\* | 1,629 | ,012 | -9,589 | -1,035 |
|  | 3 | 1 | 2,311 | 1,965 | ,762 | -2,847 | 7,469 |
|  |  | 2 | 5,312\* | 1,629 | ,012 | 1,035 | 9,589 |
|  |  |  |  |  |  |  |  |
| --- | --- | --- | --- | --- | --- | --- | --- |
| Se basa en medias marginales estimadas | | | | | | | |
| \*. La diferencia de medias es significativa en el nivel ,05. | | | | | | | |
| b. Ajuste para varias comparaciones: Bonferroni. | | | | | | | |
|  |  |  |  |  |  |  |  |

3. Ejerc \* Muscu

Pruebas multivariantePruebas multivariante, tabla, 1 niveles de cabeceras de columna y 2 niveles de cabeceras de fila, tabla con 10 columnas y 29 filas

|  |  |  |  |  |  |  |  |  |  |
| --- | --- | --- | --- | --- | --- | --- | --- | --- | --- |
| Muscu | | Valor | F | gl de hipótesis | gl de error | Sig. | Eta parcial al cuadrado | Parámetro sin centralidad | Potencia observadab |
| 1 | Traza de Pillai | ,662 | 17,626a | 2,000 | 18,000 | ,000 | ,662 | 35,251 | ,999 |
|  | Lambda de Wilks | ,338 | 17,626a | 2,000 | 18,000 | ,000 | ,662 | 35,251 | ,999 |
|  | Traza de Hotelling | 1,958 | 17,626a | 2,000 | 18,000 | ,000 | ,662 | 35,251 | ,999 |
|  | Raíz mayor de Roy | 1,958 | 17,626a | 2,000 | 18,000 | ,000 | ,662 | 35,251 | ,999 |
| 2 | Traza de Pillai | ,726 | 23,843a | 2,000 | 18,000 | ,000 | ,726 | 47,686 | 1,000 |
|  | Lambda de Wilks | ,274 | 23,843a | 2,000 | 18,000 | ,000 | ,726 | 47,686 | 1,000 |
|  | Traza de Hotelling | 2,649 | 23,843a | 2,000 | 18,000 | ,000 | ,726 | 47,686 | 1,000 |
|  | Raíz mayor de Roy | 2,649 | 23,843a | 2,000 | 18,000 | ,000 | ,726 | 47,686 | 1,000 |
| 3 | Traza de Pillai | ,708 | 21,850a | 2,000 | 18,000 | ,000 | ,708 | 43,699 | 1,000 |
|  | Lambda de Wilks | ,292 | 21,850a | 2,000 | 18,000 | ,000 | ,708 | 43,699 | 1,000 |
|  | Traza de Hotelling | 2,428 | 21,850a | 2,000 | 18,000 | ,000 | ,708 | 43,699 | 1,000 |
|  | Raíz mayor de Roy | 2,428 | 21,850a | 2,000 | 18,000 | ,000 | ,708 | 43,699 | 1,000 |
| 4 | Traza de Pillai | ,807 | 37,616a | 2,000 | 18,000 | ,000 | ,807 | 75,231 | 1,000 |
|  | Lambda de Wilks | ,193 | 37,616a | 2,000 | 18,000 | ,000 | ,807 | 75,231 | 1,000 |
|  | Traza de Hotelling | 4,180 | 37,616a | 2,000 | 18,000 | ,000 | ,807 | 75,231 | 1,000 |
|  | Raíz mayor de Roy | 4,180 | 37,616a | 2,000 | 18,000 | ,000 | ,807 | 75,231 | 1,000 |
| 5 | Traza de Pillai | ,690 | 19,999a | 2,000 | 18,000 | ,000 | ,690 | 39,998 | 1,000 |
|  | Lambda de Wilks | ,310 | 19,999a | 2,000 | 18,000 | ,000 | ,690 | 39,998 | 1,000 |
|  | Traza de Hotelling | 2,222 | 19,999a | 2,000 | 18,000 | ,000 | ,690 | 39,998 | 1,000 |
|  | Raíz mayor de Roy | 2,222 | 19,999a | 2,000 | 18,000 | ,000 | ,690 | 39,998 | 1,000 |
| 6 | Traza de Pillai | ,376 | 5,428a | 2,000 | 18,000 | ,014 | ,376 | 10,856 | ,778 |
|  | Lambda de Wilks | ,624 | 5,428a | 2,000 | 18,000 | ,014 | ,376 | 10,856 | ,778 |
|  | Traza de Hotelling | ,603 | 5,428a | 2,000 | 18,000 | ,014 | ,376 | 10,856 | ,778 |
|  | Raíz mayor de Roy | ,603 | 5,428a | 2,000 | 18,000 | ,014 | ,376 | 10,856 | ,778 |
|  |  |  |  |  |  |  |  |  |  |
| --- | --- | --- | --- | --- | --- | --- | --- | --- | --- |
| Cada F prueba los efectos simples multivariantes de Ejerc dentro de cada combinación de niveles de los otros efectos mostrados. Estas pruebas se basan en las comparaciones por parejas linealmente independientes entre las medias marginales estimadas. | | | | | | | | | |
| a. Estadístico exacto | | | | | | | | | |
| b. Se ha calculado utilizando alpha = ,05 | | | | | | | | | |
|  |  |  |  |  |  |  |  |  |  |

Gráficos de perfil

Gráficos de perfil

IBM SPSS Informe web

X

Acerca de

|  |
| --- |
| Creado con: IBM SPSS Statistics 26 |
| Fecha de creación: feb 24, 2020 |
| Versión del documento: OriginalCopia guardada |
| Fecha guardada:  feb 24, 2020 |

Controles de navegación

|  |
| --- |
| Contenido - Abre y cierra la lista de gráficos y tablas en el informe web |
| Siguiente & Anterior - Muestra la tabla o gráfico siguiente o anterior en el informe web |
| Ayuda - Abre la ayuda |

Botones de la barra de herramientas

|  |  |
| --- | --- |
|  | Deshacer - Deshace el último cambio en un documento. |
|  | Editar - Abre la herramienta de Editor para tablas y gráficos. Ciertas opciones de edición sólo están disponibles cuando se está conectado a un servidor de Internet. |
|  | Guardar - Crea una nueva copia del informe web con los cambios guardados. |
|  | Imprimir - Muestra el objeto actual desde la Vista de objeto y todos los objetos de la Vista de página. |
|  | Vista de página - Cambia el informe web para que se muestren todas las tablas y gráficos en una sola página. |
|  | Vista de objeto - Cambia el informe web para que las tablas y gráficos de muestren de uno en uno. |

Conexión con un servidor

:   El estado de la conexión del informe web a un servidor de Internet se muestra en la esquina superior derecha del informe web.
:   No se requiere una conexión a Internet para abrir un informe web. Con la copia guardada del informe web puede ver todos los gráficos y tablas y tener cierta capacidad de edición, aun sin estar conectado a Internet.
:   Si el informe web está conectado a un servidor de Internet, se dispondrá de mejores capacidades de edición para tablas y gráficos.

- Si el autor ha especificado un servidor de Internet al crear el informe web, el informe web intentará conectarse al servidor automáticamente al abrirlo.
- Si el informe web no se conecta a un servidor, pulse en el mensaje de estado del servidor para abrir las herramientas para reintentar la conexión, pruebe con otro servidor o especifique una nueva dirección de servidor.
- Para obtener más información sobre cómo añadir los controles mejorados al servidor de Internet, vaya a https://developer.ibm.com/predictiveanalytics.
- Si especifica una nueva conexión de servidor, el formato preferido es http://xxx.xxx.xxx.xxx:xxxx.

Edición de tablas

|  |  |
| --- | --- |
| Parte de esta funcionalidad sólo está disponible cuando se conecta a un servidor de Internet. | |
|  | Crear un gráfico - Crea un gráfico a partir de las celdas seleccionadas en la tabla. |
|  | Girar y ordenar - Transponer, ordenar y girar la tabla. |
|  | Color de fondo - Color de fondo de las celdas seleccionadas. |
|  | Color y estilo del texto - Color, estilo y tamaño de la letra. |
|  | Formato de numeración - Color, estilo y tamaño de la letra. |

Edición de gráficos

|  |  |
| --- | --- |
| Todo esta funcionalidad sólo está disponible cuando se conecta a un servidor de Internet. | |
|  | Tamaño del gráfico - Cambia la altura y la anchura del gráfico |
|  | Color de fondo - Color de fondo del objeto seleccionado. |
|  | Estilo de línea y borde - Color y grosor de la línea o del borde. |
|  | Color y estilo del texto - Color, estilo y tamaño de la letra. |
|  | Formato de numeración - Color, estilo y tamaño de la letra. |
|  | Propiedades de los ejes - Cambia y escala los títulos y las marcas de los ejes. |

Conexión de servidor

X
  
Conexiones de servidor guardadas  
   
  
  
  
Estado

Añadir un gráfico

Girar y ordenar

Tamaño del gráfico   
  

|  |  |  |
| --- | --- | --- |
|  |  |  |
|  |  |  |
| Bloquear relación de aspecto | | |

Fondo   

|  |  |  |  |  |  |
| --- | --- | --- | --- | --- | --- |
|  | |  | |  | |
|  |  |  |  |  |  |
|  |  |  |  |  |  |
|  |  |  |  |  |  |

Líneas y bordes   

|  |  |  |  |  |  |
| --- | --- | --- | --- | --- | --- |
|  | |  | |  | |
|  |  |  |  |  |  |
|  |  |  |  |  |  |
|  |  |  |  |  |  |

Formato del texto   

|  |  |  |  |  |  |
| --- | --- | --- | --- | --- | --- |
|  | |  | |  | |
|  |  |  |  |  |  |
|  |  |  |  |  |  |
|  |  |  |  |  |  |

  

|  |  |  |
| --- | --- | --- |
|  |  |  |

  

|  |  |  |  |
| --- | --- | --- | --- |
|  |  |  | Familia de fuente  .AppleSystemUIFont .SF NS Mono Al Bayan Al Nile Al Tarikh American Typewriter Andale Mono Apple Braille Apple Chancery Apple Color Emoji Apple SD Gothic Neo Apple Symbols AppleGothic AppleMyungjo Arial Arial Black Arial Hebrew Arial Hebrew Scholar Arial Narrow Arial Rounded MT Bold Arial Unicode MS Avenir Avenir Next Avenir Next Condensed Ayuthaya Baghdad Bangla MN Bangla Sangam MN Baskerville Beirut Big Caslon Bodoni 72 Bodoni 72 Oldstyle Bodoni 72 Smallcaps Bodoni Ornaments Bradley Hand Brush Script MT Chalkboard Chalkboard SE Chalkduster Charter Cochin Comic Sans MS Copperplate Corsiva Hebrew Courier Courier New Damascus DecoType Naskh Devanagari MT Devanagari Sangam MN Dialog DialogInput Didot DIN Alternate DIN Condensed Diwan Kufi Diwan Thuluth Euphemia UCAS Farah Farisi Futura Galvji GB18030 Bitmap Geeza Pro Geneva Georgia Gill Sans Gujarati MT Gujarati Sangam MN Gurmukhi MN Gurmukhi MT Gurmukhi Sangam MN Heiti SC Heiti TC Helvetica Helvetica Neue HelvNeue Roman for IBM Herculanum Hiragino Maru Gothic ProN Hiragino Mincho ProN Hiragino Sans Hiragino Sans GB Hoefler Text Impact InaiMathi ITF Devanagari ITF Devanagari Marathi Kailasa Kannada MN Kannada Sangam MN Kefa Khmer MN Khmer Sangam MN Kohinoor Bangla Kohinoor Devanagari Kohinoor Gujarati Kohinoor Telugu Kokonor Krungthep KufiStandardGK Lao MN Lao Sangam MN Lucida Bright Lucida Grande Lucida Sans Lucida Sans Typewriter Luminari Malayalam MN Malayalam Sangam MN Marker Felt Menlo Microsoft Sans Serif Mishafi Mishafi Gold Monaco Monospaced Mshtakan Mukta Mahee Muna Myanmar MN Myanmar Sangam MN Nadeem New Peninim MT Noteworthy Noto Nastaliq Urdu Noto Sans Javanese Noto Sans Kannada Noto Sans Myanmar Noto Sans Oriya Noto Serif Myanmar Optima Oriya MN Oriya Sangam MN Palatino Papyrus Phosphate PingFang HK PingFang SC PingFang TC Plantagenet Cherokee PT Mono PT Sans PT Sans Caption PT Sans Narrow PT Serif PT Serif Caption Raanana Rockwell Sana SansSerif Sathu Savoye LET Serif Shree Devanagari 714 SignPainter Silom Sinhala MN Sinhala Sangam MN Skia Snell Roundhand Songti SC Songti TC STIXGeneral STIXIntegralsD STIXIntegralsSm STIXIntegralsUp STIXIntegralsUpD STIXIntegralsUpSm STIXNonUnicode STIXSizeFiveSym STIXSizeFourSym STIXSizeOneSym STIXSizeThreeSym STIXSizeTwoSym STIXVariants STSong Sukhumvit Set Symbol Tahoma Tamil MN Tamil Sangam MN Telugu MN Telugu Sangam MN Thonburi Times Times New Roman Trattatello Trebuchet MS Verdana Waseem Webdings Wingdings Wingdings 2 Wingdings 3 Zapf Dingbats Zapfino |

Formato de numeración   
  

|  |  |  |
| --- | --- | --- |
| 0.00 |  |  |

Opciones de eje   
  

|  |  |  |
| --- | --- | --- |
|  |  |  |
|  |  |  |
| Mostrar título del eje | | | |
| Mostrar marcas | | | |
